# Lipid-linked nucleoside triphosphates for enzymatic synthesis of hydrophobic oligonucleotides with enhanced membrane anchoring efficiency†

**DOI:** 10.1039/d2sc06718h

**Published:** 2023-03-20

**Authors:** David Kodr, Erika Kužmová, Radek Pohl, Tomáš Kraus, Michal Hocek

**Affiliations:** a Institute of Organic Chemistry and Biochemistry, Czech Academy of Sciences Flemingovo namesti 2 CZ-16610 Prague 6 Czech Republic tomas.kraus@uochb.cas.cz hocek@uochb.cas.cz; b Department of Organic Chemistry, Faculty of Science, Charles University in Prague Hlavova 8 Prague-2 12843 Czech Republic

## Abstract

We designed and synthesized a series of 2′-deoxyribonucleoside triphosphates (dNTPs) bearing various lipid moieties. Fatty acid- and cholesterol-modified dNTPs proved to be substrates for KOD XL DNA polymerase in primer extension reactions. They were also mutually compatible for simultaneous multiple incorporations into the DNA strand. The methodology of enzymatic synthesis opened a pathway to diverse structurally unique lipid-ON probes containing one or more lipid units. We studied interactions of such probes with the plasma membranes of live cells. Employing a rational design, we found a series of lipid-ONs with enhanced membrane anchoring efficiency. The in-membrane stability of multiply modified ONs was superior to that of commonly studied ON analogues, in which a single cholesterol molecule is typically tethered to the thread end. Notably, some of the probes were detected at the cell surface even after 24 h upon removal of the probe solution. Such an effect was general to several studied cell lines.

## Abstract 2

New lipid-linked 2′-deoxyribonucleoside triphosphates were synthesized and used for enzymatic construction of various lipid–oligonucleotide conjugates capable of efficient anchoring to plasma membranes.

## Graphical abstract

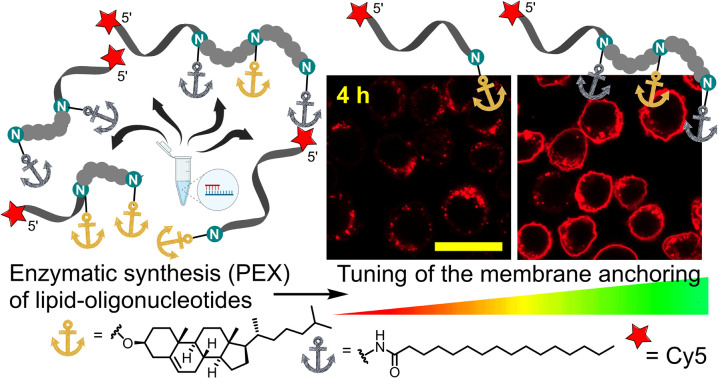

## Introduction

Recently, lipid–oligonucleotide conjugates (LONs) have attracted considerable interest of research groups among scientific fields ranging from biochemistry, chemical biology and medicine to supramolecular chemistry, nanotechnology and materials science.^1–5^ Their unique amphiphilic, programable and self-recognizing properties offer a high degree of versatility. To this date, LONs have been studied for their possible use as agents in antisense oligonucleotide therapy,^6–9^ immunotherapy,^10,11^ drug delivery,^12–14^ liposomal vehicle fusion,^15–17^ or cell adhesion,^18–20^ as well as for the fabrication of membrane biosensors,^21,22^ synthetic membrane channels,^23^ nanopores^24^ and other highly ordered nanomaterials.^2,25^ The common phenomenon in most of the above-mentioned applications is the ability of LONs to interact with phospholipid bilayers by means of noncovalent hydrophobic interactions. In other words, LONs are capable of programable modification of cellular membranes,^5,26,27^ bacterial membranes,^28,29^ or even plasma membranes in living organisms.^30^ Even though the whole process is yet not fully understood, there are several known factors driving the kinetics and dynamics of such interactions affecting the insertion rate, quantity and stability of LONs in the plasma membrane.^27,31,32^

Two major synthetic pathways are commonly employed in LON synthesis. The first approach relies on phosphoramidite building blocks and pre-modified solid supports^33^ allowing insertion of modification anywhere in the sequence. The second approach based on postsynthetic bioconjugation offers a facile access to a broad range of modifications,^34^ but typically only at terminal positions. Some of the LONs, as well as phosphoramidite building blocks are now commercially available. Yet there are some limitations of both approaches, due to the markedly altered nature of LONs. Lipophilic modifications might alter oligonucleotide solubility and decrease their further reactivity, and thus reaction conditions must be often precisely controlled and optimized.^35^ Limitations of solid-phase synthesis of hypermodified oligonucleotides in common with generally high costs of phosphoramidite modified-nucleoside building blocks restrict their exploration and reduce the immense potential of these compounds. As a result, only a few examples of multiply modified LONs were explored and studied,^36–39^ despite the fact that these studies revealed that several lipophilic modifications enhance the LON–membrane anchoring. Arguably, addition of other functional moieties to LONs might give rise to smart bifunctional membrane sensors and nanodevices. The development of new and efficient synthetic methods for the preparation of highly modified LONs is therefore desirable.

Enzymatic oligonucleotide synthesis proved to be a useful tool for the preparation of diverse modified nucleic acids. We and others have recently shown that such an approach often allows multiple base-site modifications and their combination.^40–43^ Once there exists a reliable protocol, this approach is then often universal for various sequences, or might require only minor optimizations in terms of enzyme quantity or reaction time adjustments. The enzymatic synthesis employing modified nucleoside triphosphates (dNTPs) offers a toolbox for fast, straightforward synthesis, allowing exploration of novel functional modified-oligonucleotide moieties.

Several DNA polymerases (*i.e.* KOD XL, KOD (exo^−^), Therminator, Phusion, Vent (exo^−^)) are known to accept dNTPs linked to a large variety of lipophilic and bulky modifications. Aliphatic^41^ and aromatic hydrocarbons,^44^ various fluorophores,^45^ amino acid side chains,^41^ diamondoids,^46^ carboranes,^47^ metallacarboranes,^43^ ferrocenes,^48^ polyethyleneglycols,^49^ or bile acids^50^ were introduced into the position C5 of pyrimidines and C7 of 7-deazapurines in dNTPs and used for the enzymatic construction of modified DNA containing hydrophobic or amphiphilic functionalities. In this paper, we describe the design and synthesis of lipid-linked (fatty acid, cholesterol or diacylphosphoglycerol) modified dNTPs; we investigated their DNA polymerase substrate activity and the possibility of introducing multiple modifications into the oligonucleotide structure. Last but not least, we studied LON interactions with cellular membranes and implemented our observations into the design and synthesis of novel structures with enhanced in-membrane stability.

## Results and discussion

### Synthesis

In order to mimic the main lipid components of cellular membranes, we designed 2′-deoxyribonucleoside triphosphates (dNTP) decorated with a fatty acid, cholesterol or diacylphosphoglycerol moiety. Positions C5 of pyrimidines and C7 of 7-deazapurines are known to be suitable sites for attaching the modifications to dNTPs in order to preserve their Watson–Crick base pairing capability. Even bulky modifications are well tolerated by several DNA polymerases when tethered *via* a sufficiently long flexible linker.^51,52^ The attachment of lipid modifications was envisaged either through the Sonogashira cross-coupling^53,54^ reactions of iodinated nucleosides (or nucleotides) or through the Cu-catalyzed Alkyne Azide Cycloaddition (CuAAC)^55^ of 5-azidomethyl-2′-deoxyuridine with relevant alkynes.

**Scheme 1:**
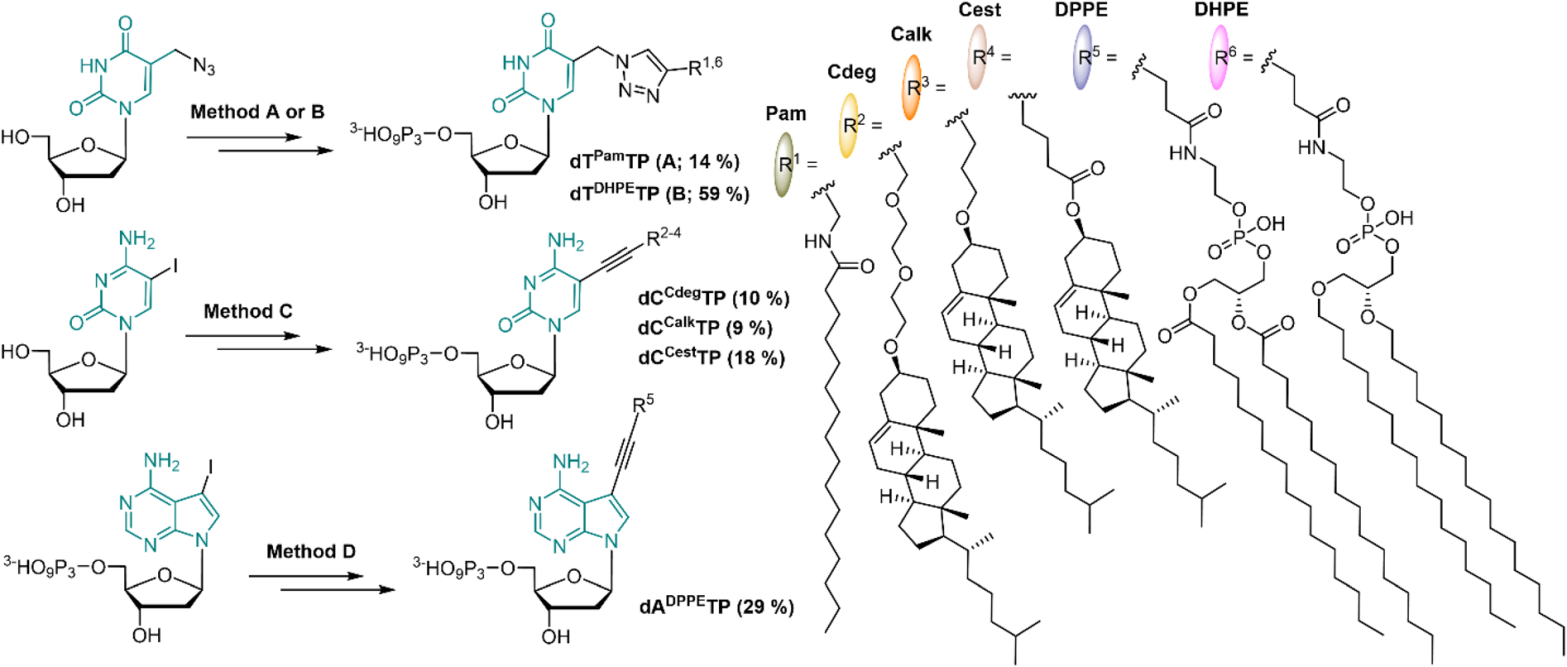
Synthesis of lipid-modified dN^R^TPs; Method A: CuAAC on nucleoside (i), triphosphorylation (ii); Method B: triphosphorylation (ii), CuAAC on dNTP (iii); Method C: Sonogashira cross-coupling (iv), triphosphorylation (ii); Method D: Sonogashira cross-coupling on dNTP (v). Reagents and conditions: (i) alkyne, sodium ascorbate, CuSO_4_·5 H_2_O, DMF, 16 h, 22 °C; (ii) (1) POCl_3_, PO(OMe)_3_, 0 °C, 2 h; (2) pyrophosphate, Bu_3_N, DMF, 1 h, 0 °C; (3) TEAB, 22 °C, 15 min; (iii) alkyne, sodium ascorbate, CuSO_4_·5 H_2_O, DMSO, 4 h, 22 °C; (iv) alkyne, Pd(PPh_3_)_4_, CuI, DMF, 75 °C, 2 h; (v) alkyne, Pd(PPh_3_)_2_(Cl)_2_, CuI, Et_3_N, DMF, 75 °C, 1 h.

Palmitic acid was chosen as a simple representative of fatty acids. It was conjugated with propargylamine using *N*,*N*′-dicyclohexylcarbodiimide; the formed *N*-propargylpalmitamide (1) then underwent the CuAAC reaction with 5-azidomethyl-2′-deoxyuridine to give dT^Pam^ (2), and subsequent triphosphorylation and ion pair RP-HPLC purification gave the desired dT^Pam^TP (3) in a moderate 14% yield (Scheme 1 and S1†).

The cholesterol moiety was tethered to the terminal alkyne either through an ester or an ether linkage *via* an oxygen atom at the position C3 of the steroid skeleton. The esterification of the hydroxyl group with 4-pentynoic acid gave the cholesterol ester alkyne 4 which was used for the Sonogashira cross-coupling reaction with C5 iodinated cytidine to form compound 5 (dC^Cest^). Standard triphosphorylation reaction yielded dC^Cest^TP (6) in a moderate 18% yield (Scheme 1 and S2†).

A presumably more stable ether linkage was achieved by reaction of the cholesterol with a corresponding ethynyl-linked alcohol under heterogenous catalysis using Montmorillonite K10 clay.^56^ We used two ethynyl-linked alcohols: a longer propargyl-diethyleneglycol and a shorter 4-pentyn-1-ol. The reactions were performed under microwave irradiation^57^ and were highly stereoselective with configuration retention on the chiral C3 centre (7 and 10). The Sonogashira reactions of the terminal alkynes 7 and 10 with 5-iodocytidine followed by triphosphorylation reaction yielded corresponding nucleotides dC^Cdeg^TP and dC^Calk^TP (9 and 12, respectively) in moderate yields of 9–10% (Scheme 1, S3 and S4†).

In order to construct a nucleotide bearing a phospholipid moiety, 1,2-dipalmitoyl-*sn-glycero*-3-phosphoethanolamine was reacted with an *N*-hydroxysuccinimide (NHS) activated ester of 4-pentynoic acid to form a phospholipid-alkyne conjugate 13, which was directly coupled with 7-deaza-7-iodo-dATP under aqueous Sonogashira cross-coupling conditions to afford dA^DPPE^TP (14) in 29% yield (Scheme 1 and S5†). Furthermore, a dNTP modified with a dialkylphosphoglycerol moiety, wherein both aliphatic chains are tethered to a phosphoglycerol *via* a more stable ether bond was prepared. We synthesized analogously the corresponding terminal alkyne 15, but unfortunately it did not react with 7-deaza-7-iodo-dATP following the same synthetic procedure, nor under any other tested cross-coupling conditions. Eventually, we succeeded in attaching the dialkylphosphoglycerol moiety through CuAAC with 5-azidomethyl-2′-deoxyuridine triphosphate; the reaction gave dT^DHPE^TP (16) in a 59% yield (Scheme 1 and S6†).

### Enzymatic incorporation

**Fig. 1:**
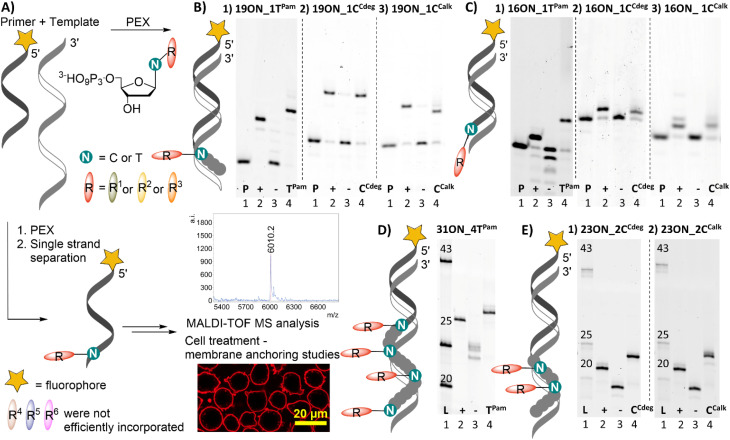
(A) PEX using single modified dNTPs and fluorophore labelled prim^*rnd*^ (B) denaturing PAGE analysis of PEX using KOD XL, (1) temp^*1T*^, dT^Pam^TP; (2) temp^*1C*^, dC^Cdeg^TP; (3) temp^*1C*^, dC^Calk^TP; (C) denaturing PAGE analysis of PEX using (1) KOD XL, temp^*1T*^, dT^Pam^TP; (2) Vent (exo^−^), temp^*TermC*^, dC^Cdeg^TP; (3) Vent exo^−^, temp^*TermC*^, dC^Calk^TP; (D) native page analysis of PEX using KOD XL, temp^*rnd16*^, dT^Pam^TP; (E) native page analysis of PEX using KOD XL, temp^*rnd8*^ (1) dC^Cdeg^TP; (2) dC^Calk^TP; [lanes (1) prim^*rnd*^ for denaturing and dsDNA ladder for native PAGE; (2) positive control, natural dNTPs; (3) negative control, without studied dNTP; (4) reaction containing modified dN^R^TP].

All prepared dNTPs were subjected to primer extension (PEX) experiments to test their suitability as substrates for DNA polymerase using a 16-, 19-, 23- or 31-mer template and 5′-(6-FAM)-labelled primer (Fig. 1, for sequences see Table S1†). KOD XL was selected as the first-choice polymerase for dNTPs bearing bulky lipophilic modifications based on our previous experience and published data.^58^ The PEX progress was monitored by polyacrylamide gel electrophoretic analysis (PAGE). Attempted HPLC analysis of the PEX products on C18 or even C8 columns failed because the lipid-linked amphiphilic LONs tended to remain on the column.^59^ Therefore, the purity of the products was verified by quantifying images of PAGE analyses (Fig. S11†) to confirm that the PEX product homogeneity is ≥90%. The product identity was confirmed by MALDI-TOF mass spectrometry upon single strand streptavidin magnetic bead separation using 5′-dualbiotinylated templates.^60^ MS data are presented in Table 1 and the whole spectra can be found in the ESI.† Some of the promising substrates were as well tested in polymerase chain reaction (PCR) and terminal deoxyribonucleotidyl transferase (TdT) elongation experiments.

**Table 1:** List of prepared LONs; MS characterization; confocal images of treated HL-60 cells^a^

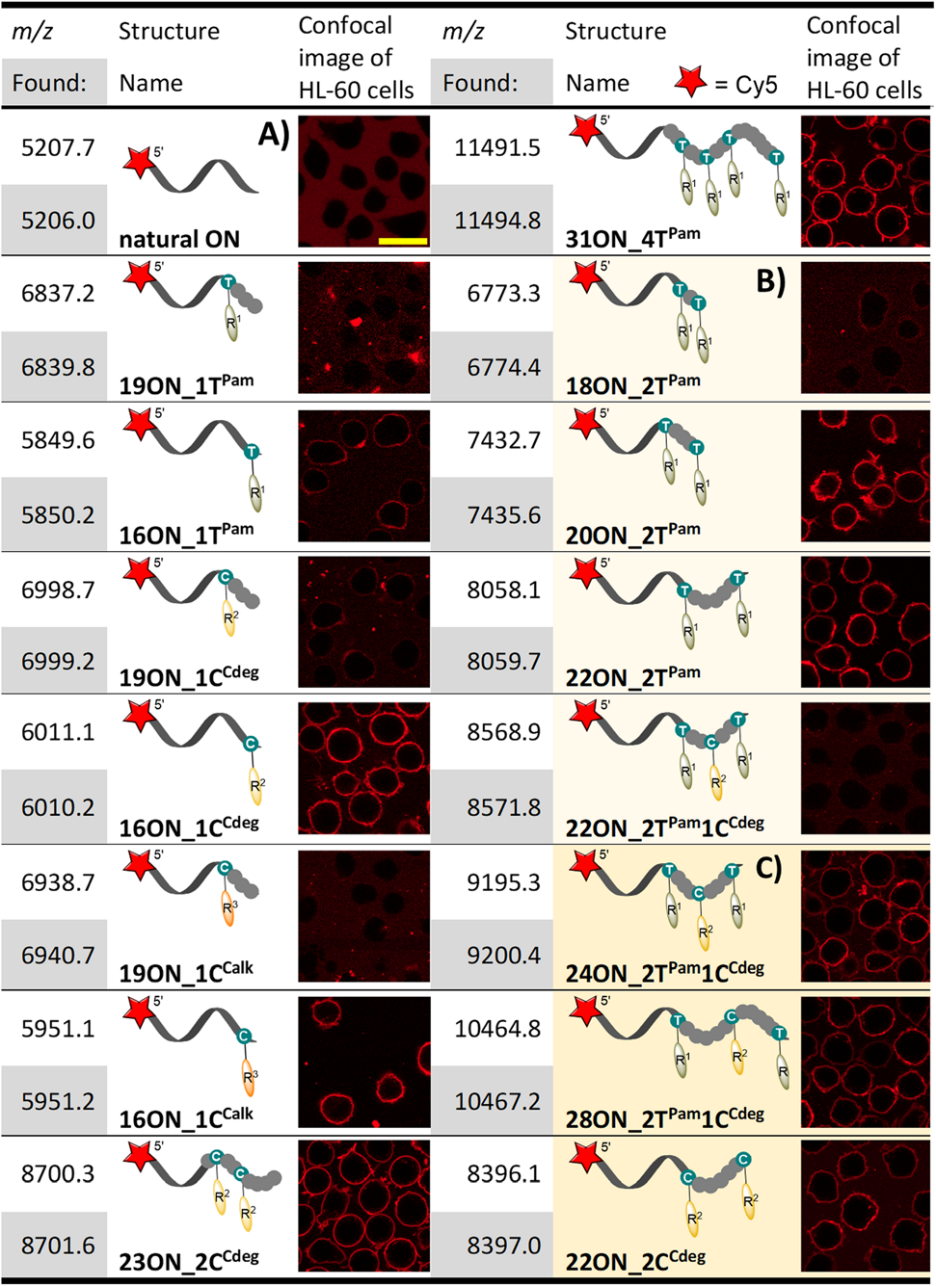

a HL-60 cells were treated with 1 μM solution (10 min; 37 °C) of given LON in PBS. Intensive fluorescence staining of cell plasma membranes is apparent in the cases when the membrane anchoring was efficient, whereas cells with unstained plasma membranes (as in the control − natural ON) indicate less efficient or no insertion of LONs. The yellow marker size is 20 μm.

Both fatty acid and cholesterol modified dN^R^TPs (namely: dT^Pam^TP, dC^Cdeg^TP and dC^Calk^TP) proved to be good substrates for KOD XL DNA polymerase in single nucleotide PEX with a 19-mer template (Fig. 1B). It ought to be mentioned that bands of the full-length products of enzymatic elongation using dC^Cdeg^TP and dC^Calk^TP were shifted downwards (faster mobility) compared to the positive control (a natural ON) upon denaturing PAGE analysis in contrast to the majority of cases, in which modified ONs are typically shifted upwards. Yet, MS analysis confirmed the identity of expected full-length products (Table 1). We do not know the reason for the unexpected faster electrophoretic mobility of some of the lipid-modified ON sequences, but we have previously observed similar behavior with ONs bearing a lipophilic methylated bodipy fluorophore.^61^ Surprisingly, KOD XL DNA polymerase poorly incorporated modified dC^R^ nucleotides into a terminal position using a 16-mer template (single-nucleotide extension of the primer). Even after several optimizations and reaction time extensions only shorter ON fragments appeared, which might be attributed to its exonuclease activity. This was overcome by use of Vent (exo^−^) or Therminator DNA polymerases (Fig. 1C). Multiple incorporations of up to four dT^Pam^ nucleotides (Fig. 1D) proceeded smoothly using a 31-mer template, but the denaturing PAGE analysis of multiply modified ON showed only smeared bands, which was probably caused by ssON product aggregation.^62^ This was resolved by native PAGE analysis of a double-stranded product which showed relatively sharp bands. In contrast, the synthesis of multiply modified 31-mer ON (4 modifications per strand) using cholesterol-linked dC^Cdeg^TP or dC^Calk^TP as substrates could not be achieved with any of the mentioned polymerases (data not shown). Therefore, we truncated the template sequence to a 23-mer (encoding for double incorporation of each nucleotide). In this case the full-length products containing these cholesterol-linked nucleotides were obtained (Fig. 1E). These experiments demonstrated that the fatty acid-linked dT^Pam^TP was a better substrate than the cholesterol-linked nucleotides. Among them, dCTP with a tethered cholesterol moiety through a longer diethylene glycol linker (dC^Cdeg^TP) performed better (a lower amount of enzyme was required for multiple incorporations) than the shortly linked dC^Calk^TP. Thus, dT^Pam^TP and dC^Cdeg^TP were selected for further more challenging PEX experiments and membrane interaction studies.

The other three nucleotides: dC^Cest^TP, dA^DPPE^TP and dT^DHPE^TP did not perform well. MS analyses of 19-mer products of PEX reactions employing dC^Cest^TP (Fig. S4†) and dA^DPPE^TP (Fig. S5†), respectively, revealed cleavage of the ester bonds within the products. Incubation of dC^Cest^TP and dA^DPPE^TP under PEX reaction conditions in the buffer and in pure H_2_O with subsequent UPLC-MS analysis revealed inherent instability of these ester bonds in aqueous solutions and further confirmed that the ester linkage is insufficiently stable for attachment of modifications at dNTPs for the use in PEX (Fig. S12 and S13†). The dialkylphospoglycerol-linked dT^DHPE^TP was found to be a poor substrate (high enzyme concentrations required) and we were able to detect only traces of 19-mer and 16-mer PEX products by both PAGE and MS analysis (Fig. S6†). We tried to resolve this problem by addition of an equimolar amount (with respect to dT^DHPE^TP) of methyl-β-cyclodextrin or heptakis(2,3-di-*O*-methyl)-β-cyclodextrin^63^ to minimize the possibility of aggregation, but it did not improve the outcome of the reaction. Therefore, we did not study dT^DHPE^TP any further.

The fatty acid-linked thymidine and cholesterol-modified cytidine triphosphates were tested as substrates for PCR, but no amplification was observed (Fig. S7†). This observation somewhat corresponds to our previous findings for other bulky lipophilic modifications of dNTPs.^43,47^ Next, we tested TdT elongation which would allow direct facile 3′-end modification of single stranded ON.^64^ The TdT, however, did not efficiently incorporated these lipid-modified nucleotides (Fig. S8 and S9†) and the inhibition of TdT was rather apparent at higher concentrations of dT^Pam^TP (Fig. S9†).

### Membrane anchoring experiments, preliminary studies with basic PEX products

Having prepared and characterized a first set of LONs differing in number and position(s) of lipophilic modifications, we investigated the interactions of these LONs with the plasma membranes of live cells. To this end, we enzymatically prepared above-mentioned LONs using a 5′-Cy5-labelled primer and 5′-dualbiotin template. After the PEX, we separated and purified single stranded LONs by streptavidin-biotin magnetic separation. Concentrations of all LONs were adjusted to 1 μM in PBS buffer using UV-VIS absorbance of the Cy5 label (*λ*_max_ = 646 nm). HL-60 cells (deposited on polylysine-coated glass) were treated with 1 μM solutions of LONs and incubated for 10 min at 37 °C before the confocal fluorescence microscopy image was taken (Table 1). Intensive fluorescence staining of the cellular membrane is apparent in the cases when the membrane anchoring occurred. Conversely, the images of cells with unstained plasma membranes (uncircumscribed dark spots) represent cases of inefficient membrane anchoring as can be seen also in the control experiment performed with the 15-mer natural ON (5′-Cy5-labelled prim^*rnd*^). In all experiments, we observed an occasional localization of intensive fluorescence inside the cells, which were apparently apoptotic. This is in accordance with previously published findings.^26^

The 16-mer LONs bearing fatty acid or cholesterol moieties at the 3′-end (terminal modification) performed well in the membrane anchoring experiments and resulted in an intensive fluorescence staining of the cellular membrane (Table 1A). Interestingly, 19-mer LONs where the lipid modifications are not at the sequence terminus (the modified nucleotide is in-between 15- and 3-nucleotide sequences; 5′- to 3′-end direction) were rather poorly anchored. This can be explained by wrapping of the natural nucleotides around the hydrophobic moiety, thus shielding it from interactions with the membrane.^62^ On the other hand, multiply lipid-modified 23ON_2C^Cdeg^ and 31ON_4T^Pam^ showed effective membrane anchoring despite the fact that some of the lipid modifications were not positioned at the terminus of the sequence. When we replaced the probe solution after the initial incubation with the cell culture medium, we observed immediate decay of fluorescent signal intensity on the cellular membrane (data not shown). Particularly in the case of 16ON_1T^Pam^ the fluorescence signal was lost immediately. The rapid in-membrane insertion of terminally modified LONs and their subsequent release upon medium replacement are known LON properties and our observations were consistent with already published results.^27^

### Second generation LONs, exploring the scope of enzymatic synthesis

Learning from the above-described results, we designed a new series of templates, encoding for enzymatic incorporation of multiply lipid-modified nucleotides at the 3′-end. These were designed to allow either incorporation of two equally modified nucleotides at a varying distance, or to combine different lipid-linked nucleotides and create unique conjugates containing both palmitate and cholesterol. The designed 18-, 20- and 22-mer PEX products contained two dT^Pam^ and one dC^Cdeg^ nucleotides either in an adjacent position, or always separated by one or two natural nucleotides, respectively. Enzymatic incorporation of only two dT^Pam^ modified nucleotides at varying positions proceeded smoothly (18ON_2T^Pam^, 20ON_2T^Pam^, 22ON_2T^Pam^; Table 1B). On the other hand, KOD XL DNA polymerase performed poorly when preparing 18-, or 20-mer ON with three lipophilic modifications in close proximity at the terminus of the sequence. The desired PEX product was obtained only for 22-mer 22ON_2T^Pam^1C^Cdeg^, where each modified nucleotide is spaced by a two-nucleotide sequence. PAGE analysis was ambiguous in some cases due to product aggregation (Fig. S10†). However, the use of templates with 5′-dualbiotin and subsequent magnetic separation resulted in a complete removal of the template strand yielding LONs of sufficient purity. Their MALDI MS analysis further confirmed the identity and purity of the full-length products (comparison with the use of a 5′-biotin labeled template can be seen in ESI Fig. S14†). Importantly, this experiment proved our hypothesis, that both dT^Pam^TP and dC^Cdeg^TP are mutually compatible and can be used for the construction of highly modified nucleic acids. We assume that the synthesis of high-density lipid-modified sequences might be restricted due to sterical bulkiness, especially in the case of cholesterol-linked nucleotides.

The densely modified thread 18ON_2T^Pam^ (Table 1B) did not significantly interact with the cell surface. Also, 22ON_2T^Pam^1C^Cdeg^ with three lipophilic modifications in close proximity performed poorly. On the other hand, both 20ON_2T^Pam^ and 22ON_2T^Pam^ with more distant lipid modifications (separated by three- and five-nucleotide sequences, respectively) were anchored in the phospholipid membrane, but they were also significantly released upon medium displacement (data not shown). The importance of the distance between two lipid modifications was quite unexpected, since one could envisage that the two lipids in adjacent positions would reinforce rather than restrict such interaction.

### Third generation LONs

Since the densely modified LONs did not perform well, we designed a new set of 24- and 28-mer templates, encoding for incorporation of dT^Pam^ and dC^Cdeg^ nucleotides spaced either by a three (24ON_2T^Pam^1C^Cdeg^) or five (28ON_2T^Pam^1C^Cdeg^) nucleotide sequence, respectively. In addition, we also designed the 22-mer template for a PEX synthesis of 22ON_2C^Cdeg^ with two dC^Cdeg^ nucleotides spaced by a five-nucleotide sequence (Table 1C). The enzymatic synthesis, product purification and identification proceeded smoothly under already established conditions (Fig. S10†). Gratifyingly, we observed efficient anchoring of these LONs in the plasma membrane in all cases. Initial examination by confocal microscopy revealed that the drop of fluorescence intensity at the plasma membrane was slower upon replacing the LON solution with the cell culture media indicating a higher stability of the anchoring as compared to the previously studied LONs.

### Nuclease degradation of LONs

Since there were some literature examples^65,66^ suggesting that tethering a single lipid moiety to the 5′-end enhances LON stability against nucleases in some specific sequences, we tested whether also our 3′-end multiple lipid modifications would cause some resistance against a nuclease mediated degradation. Therefore, we incubated a solution of natural ON or modified LONs in the presence of DNAse I for 2 h at 37 °C (Fig. S19 in the ESI†). We did not observe any enhancement of resistance against DNAse I digestion in these particular LON sequences and full degradation was observed within 10 minutes in all cases. This is not unexpected because the 5′-end and the main part of the LON are unmodified and are freely accessible for the enzyme in the solution.^11,67^

### Quantitative assessment of the LON persistence in cells

To investigate and evaluate the LON persistence in the cell membrane, we first selected 16ON_1C^Cdeg^ as the representative example, due to its structural similarity to the commonly studied 3′-TEG-cholesterol modified LONs. 16ON_1C^Cdeg^ was compared with structurally unique, multiply lipid-modified oligonucleotides, namely 31ON_4T^Pam^, 23ON_2C^Cdeg^, 22ON_2C^Cdeg^, 24ON_2T^Pam^1C^Cdeg^ and 28ON_2T^Pam^1C^Cdeg^. We established a flow cytometry-based assay to obtain reliable statistical data from more than 5000 events. In a typical experiment, HL-60 cells were incubated with a 1 μM solution of the given LON in PBS as described for the microscopy evaluation. Then the LON solution was removed, and the cells were washed and further incubated in a cell culture medium at 37 °C. Measurements of fluorescence intensities of the cells were performed by flow cytometry at several time points (0, 15, 30 min, 1, 2, 4, and 24 h). To ensure mutually comparable results, treatments of cells with LONs and subsequent measurements by flow cytometry were performed consecutively in one series.

**Fig. 2:**
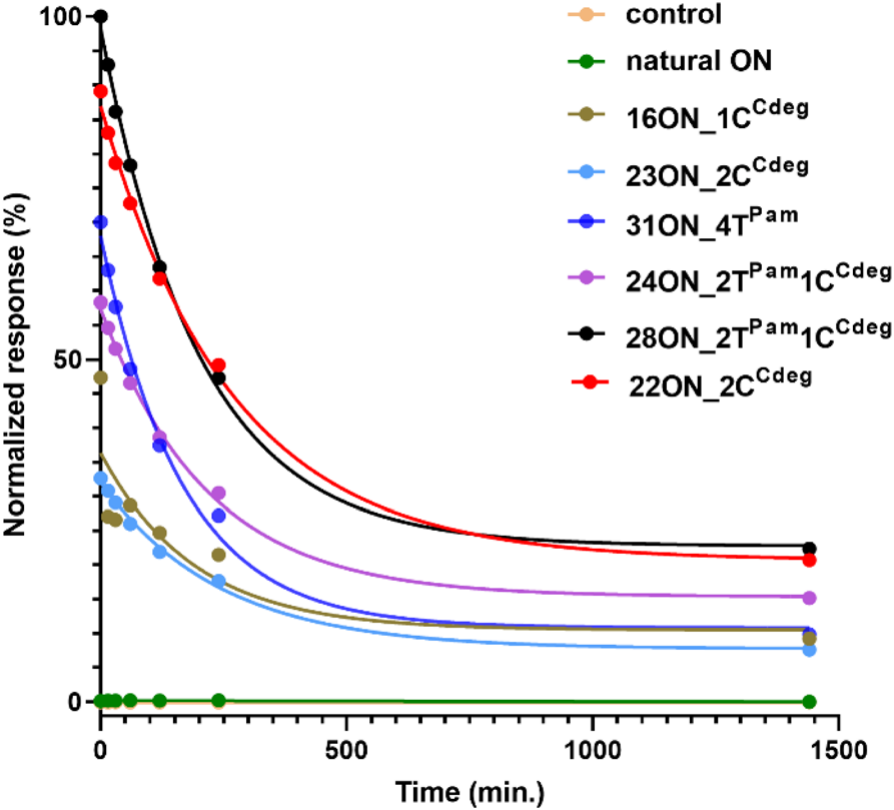
Time course of mean values of fluorescence intensity evaluated by flow cytometry on HL-60 cells. HL-60 cells were incubated with a 1 μM solution of a given LON in PBS for 10 min, washed and further incubated in L-15 medium at 37 °C. Measurements were performed at several time points (0, 15, 30 min, 1, 2, 4, and 24 h). Graph shows data from one representative experiment.

**Fig. 3:**
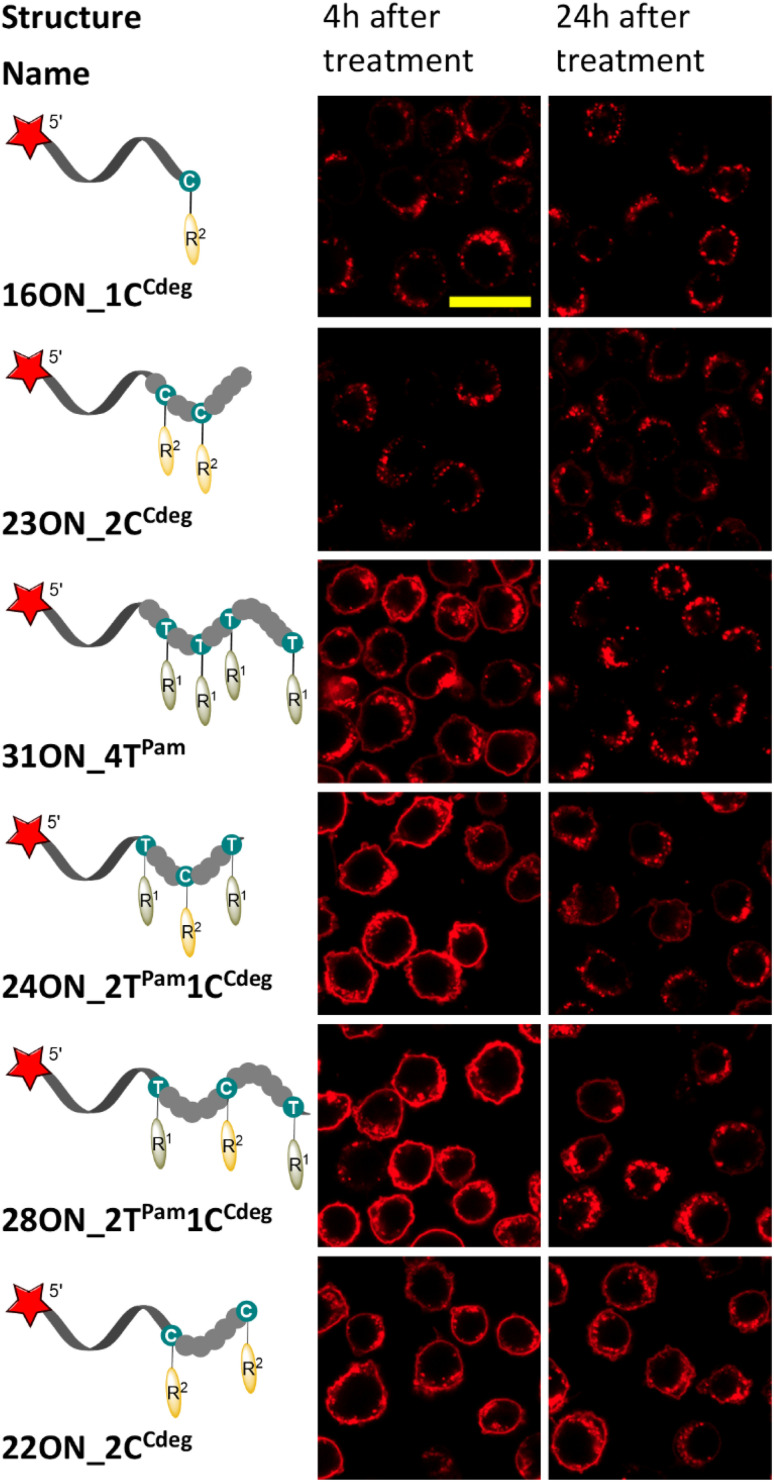
Confocal microscopy images of the samples of HL-60 cells (from the flow cytometry assays) at 4 h and 24 h time-points. The microscopy images were taken and processed under identical conditions to illustrate different LON behaviors. The yellow marker size is 20 μm.

Plots of normalized mean values of fluorescence intensities as a function of time (Fig. 2) revealed their considerable decay, particularly in the first four-hour time period. Yet, 22ON_2C^Cdeg^ (red) and 28ON_2T^Pam^1C^Cdeg^ (black) exhibit superior persistence compared to other LONs. To see the localization of the probes in cells, we investigated the samples of the cells by confocal microscopy in parallel at 4 h and 24 h time points. Imaging of the cells revealed (Fig. 3) that particularly the third generation LONs, with multiple terminal lipid modifications, were significantly anchored in the plasma membrane even after 4 hours upon the removal of LON solutions, whereas 16ON_1C^Cdeg^ was already substantially removed from the cell surface. Notably, LONs 22ON_2C^Cdeg^ and 28ON_2T^Pam^1C^Cdeg^ appeared to be present even after 24 h in the plasma membrane (Fig. 3 and S15†), although, a considerable amount of the probe was internalized in all cases, presumably as a natural consequence of endocytosis.^27^ This set of experiments revealed enhanced membrane anchoring efficiency for both 22ON_2C^Cdeg^ and 28ON_2T^Pam^1C^Cdeg^ as the total amount of retained compound was significantly higher.

### Quantification of the membrane-anchored probes by image analysis

**Fig. 4:**
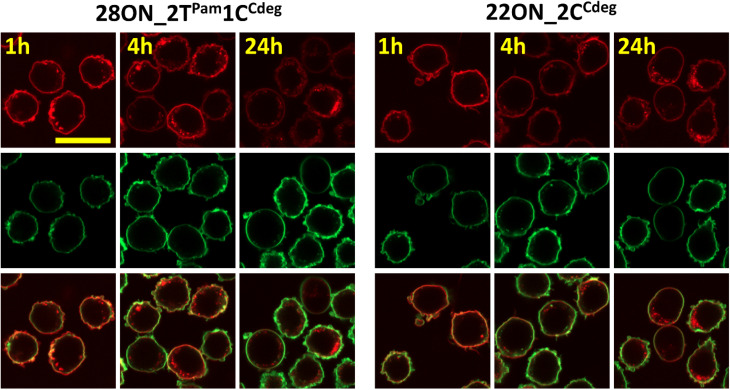
Confocal microscopy images of the samples of HL-60 cells treated with LONs 28ON_2T^Pam^1C^Cdeg^ (left) or 22ON_2C^Cdeg^ (right) and subsequently imaged at given time-points (1 h, 4 h, 24 h; in this figure) membrane staining was performed using CellMask™ Green 5–10 minutes prior imaging. Top: Cy5 – red channel; Middle: CellMask™ Green – green channel; Bottom: merged.

**Fig. 5:**
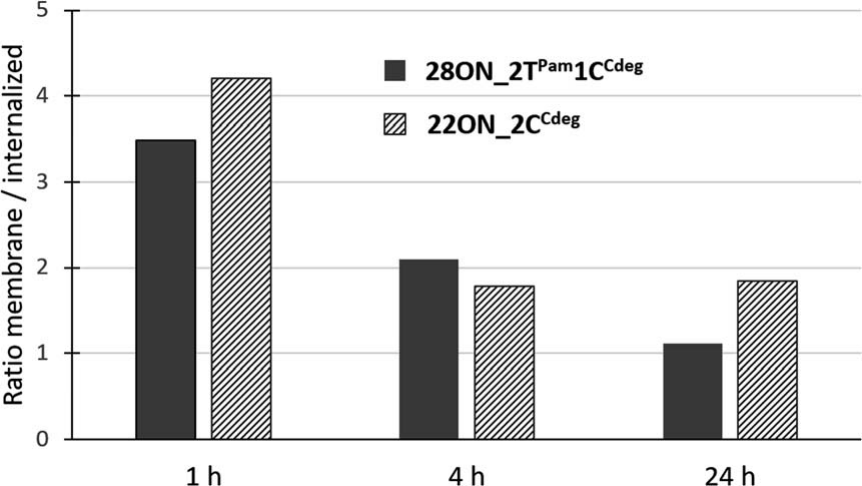
Time-evolution of ratios of fluorescence intensities detected in the membrane and in the intracellular space determined by two-color image analysis.

To assess the contributions of the fluorescence intensities of the membrane-anchored and internalized probes, which cannot be distinguished by flow cytometry, we determined their ratio by quantitative image analysis using a specific plasma membrane stain delineating the membrane contours. Thus, HL-60 cells were treated with LONs 22ON_2C^Cdeg^ or 28ON_2T^Pam^1C^Cdeg^ and subsequently imaged at given time-points (Fig. 4 and S16†). Membrane staining was performed using CellMask™ Green 5-10 minutes prior imaging. Image analysis (for detailed procedure see ESI; Fig. S17†) allowed us to calculate the ratios of probe intensities localized at the equator of the cell membrane and in the corresponding intracellular section (Fig. 5). These results indicate that the major portion of LONs was localized in the plasma membrane within the first hour; only about one fifth of the intensity comes from intracellular space. In the fourth hour, the signal intensity localized inside the cell was approximately one third of that in the membrane. After 24 hours, we observed that about a half of the signal intensity of 28ON_2T^Pam^1C^Cdeg^ came from intracellular space; interestingly 22ON_2C^Cdeg^ was still anchored in the plasma membrane in an excess (ratio membrane/internalized = 1.8).

### LON membrane anchoring efficiency in other cell lines

**Fig. 6:**
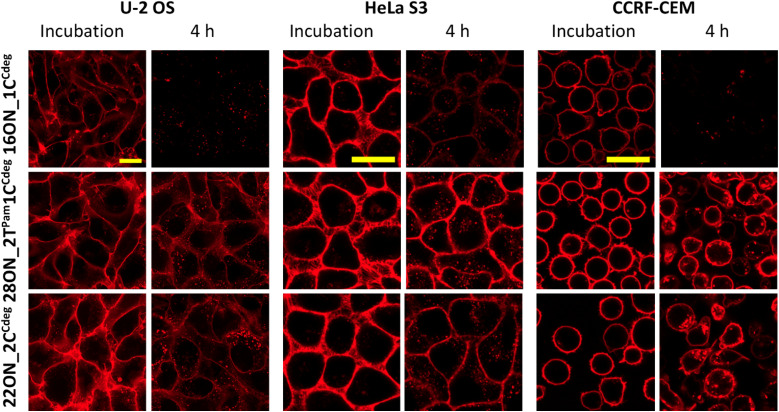
LON treatment (10 min incubation with 1 μM solution at 37 °C) and the membrane persistence (4 hour time-point upon LON solution removal) among various cell lines. The microscopic images were taken under the same settings and processed under identical conditions within each cell line. The yellow marker size is 20 μm.

Next, we studied the LON–membrane interactions in different cell types. We tested two adherent cell lines, U-2 OS and HeLa S3, along with CCRF-CEM suspension cells, and compared them with the HL-60 suspension cell line. We selected the representative 16ON_1C^Cdeg^ and two candidates with enhanced in-membrane stability, 28ON_2T^Pam^1C^Cdeg^ and the 22ON_2C^Cdeg^. 10 minute incubation with the selected LON was performed following the procedure described for the HL-60 cell line. Then the LON solution was removed and the cells were further incubated in a L-15 medium at 37 °C. The confocal microscopy imaging revealed that the LONs inserted efficiently into the membranes of all tested cells (Fig. 6). More importantly, this suggests that the enhanced membrane-anchoring effect observed for 28ON_2T^Pam^1C^Cdeg^ and 22ON_2C^Cdeg^ is comparable among the various tested cell lines (Fig. 6 and S18†). These results indicate that the multiple lipid modifications, which are located at the terminal part of the oligonucleotide backbone and sufficiently separated with a natural nucleotide sequence, reinforce LON stability within the cellular membranes and that the effect is general to various cell types.

## Conclusions

We designed and synthesized several lipid-linked dNTPs and tested their enzymatic incorporation into a DNA strand. Palmitate- and cholesterol-ether-linked dNTPs were used as substrates for KOD XL or Vent (exo^−^) DNA polymerases in a PEX and we demonstrated their mutual compatibility for simultaneous, multiple enzymatic incorporations. Unfortunately, cholesterol-ester- and phospholipid-linked dNTPs were either inherently unstable or poor substrates for all tested DNA polymerases. Modified single-stranded ONs were synthesized by PEX and isolated by a magnetoseparation protocol using 5′-dualbiotin modified templates to provide LONs of high purity for fundamental studies. However, such ssON isolation could be also performed using regular 5′-biotin modified templates, λ exonuclease digestion of 5′-phosphorylated templates, or our recently published method of traceless template and primer region RNase digestion.^68^ The PEX products can also be used directly as double stranded ONs, which can be purified using the commercially available kits for short DNA fragment purification.

We used palmitate- and cholesterol-linked dNTPs for construction of diverse LONs bearing one or several lipid modifications at the 3′-end and studied their anchoring to plasma membranes. We found that the nature and position of the lipid modification within the LON were crucial for interaction with a plasma membrane and for the stability of the anchoring. In general, LONs bearing one palmitate or cholesterol modification anchored less efficiently and were easily washed out. LONs with multiple lipid modifications at adjacent or close positions anchored poorly, whereas LONs with two cholesterols or combination of palmitate and cholesterol at sequence-distant positions anchored efficiently and were persistent in the membrane. Notably, the oligonucleotide bearing two cholesterol moieties interspaced by a five-nucleotide sequence at the 3′-end was still significantly localized on the cell surface even after 24 h upon the probe solution removal. To the best of our knowledge, this work is the first report on LONs containing two different lipid modifications and the beneficial effect of their combination. The PEX enzymatic synthesis enables an easy access to different sequences and combinations of modifications and could also be combined with other modified nucleotides and hence it might be interesting as a tool for constructing and exploring novel programmable materials for membrane modification and sensing.

## Experimental part

The complete experimental part, procedures and characterization of all compounds are given in the ESI.† Only the most important typical procedures are given below:

### CuAAC reactions

In an argon purged flask, 5-azidomethyl-2′-deoxyuridine and corresponding alkyne (1.1 equiv.) were dissolved in DMF (2 mL) and degassed with argon. A 1 M water solution of CuSO_4_ (10 mol%) and a 1 M water solution of sodium ascorbate (40 mol%) were added. The reaction was stirred for 16 h at 22 °C under an argon atmosphere and then concentrated under vacuum and the crude mixture was purified using a normal phase silica gel flash chromatograph with a DCM–MeOH mobile phase (linear gradient).

### Sonogashira cross-coupling reactions

In an argon purged flask, 2′-deoxy-5-iodocytidine, Pd(PPh_3_)_4_ (10 mol%), CuI (20 mol%) and corresponding alkyne (2 equiv.) were dissolved in dry DMF (1.5 mL) and finally Et_3_N (2.4 equiv.) was added. Reaction was stirred for 2 h at 75 °C and then concentrated under vacuum and residual DMF was removed by several co-distillations with toluene. The crude mixture was purified using a normal phase silica gel flash chromatograph with a DCM–MeOH mobile phase (linear gradient).

### Triphosphorylation reactions

A modified nucleoside was stirred under vacuum for 16 h at 65 °C. Then it was dissolved in dry PO(OMe)_3_ (0.5 mL) under an argon atmosphere and cooled on an ice-salt bath. POCl_3_ (1.2 equiv.) was added through a septum and stirred for 2 h at 0 °C. In another argon purged flask, bis(tributylammonium) pyrophosphate (5 equiv.) was dissolved in dry DMF (0.5 mL) and after subsequent addition of Bu_3_N (4 equiv.) and cooling down on an ice-salt bath, the mixture was added to the reaction. The reaction was stirred for an additional 1 h at 0 °C. 2 M TEAB (0.5 mL) was added dropwise and stirred for 15 min at 22 °C. The reaction was concentrated under vacuum. The product was purified on RP-HPLC with use of a linear gradient of 0.1 M TEAB (triethylammonium bicarbonate) in H_2_O to 0.1 M TEAB in H_2_O/MeOH (1 : 1) to MeOH as eluent in 90 minutes.

### Analytical PEX reactions

The reaction mixture (10 μL) contained template temp^*1N*^ (3 μM, 0.75 μL) 5′-(6-FAM)-labelled primer prim^*rnd*^ (3 μM, 0.5 μL), dGTP (0.5 mM, 0.5 μL), and dT^Pam^TP (0.5 mM, 1 μL); or dC^Cdeg^TP, or dC^Calk^TP (0.1 mM, 1 μL); KOD XL DNA polymerase (0.3 U) and a reaction buffer (10×, 1 μL) as supplied by the manufacturer. The reaction mixture was incubated for 30 minutes at 60 °C. The PEX reaction was stopped by addition of PAGE stop solution (10 μL) and heated for 2 minutes at 95 °C. Samples were analyzed by denaturing PAGE and visualized using fluorescence imaging.

### Enzymatic synthesis of multiply modified LON for cell experiments

The reaction mixture (50 μL) contained 5′-dual biotin-labelled template temp^*TCT*^, or temp^*T(1)C(1)T*^, or temp^*T(2)C(2)T*^, or temp^*T(3)C(3)T*^, or temp^*T(5)C(5)T*^ (100 μM, 1 μL); 5′-(Cy5)-labelled primer prim^rnd^ (100 μM, 1 μL), corresponding natural dNTPs (4 mM, 1 μL), dT^Pam^TP and dC^Cdeg^TP (each 2 mM, 1 μL) and KOD XL DNA polymerase (3.75 U) in the enzyme reaction buffer (10×, 5 μL) as supplied by the manufacturer. The reaction mixture was incubated for 2 h at 60 °C.

### Live cell imaging

The cells were cultured under conditions specified above. 24 hours prior to experiments, the cells were seeded into a 96 Well Glass Bottom Plate (Cellvis, P96-1.5H–N) in this manner: (i) suspension cells (HL-60, CCRF-CEM) were transferred to the complete Leibowitz L-15 medium (10% FCS) and seeded into wells, in which the bottom glass had been pre-coated (2 hours at 37 °C) with poly-d-lysine hydrobromide (cat. no. P6407, Sigma-Aldrich), at the density of 50k per well in 100 μL of complete L-15 medium; (ii) adherent cells (U-2 OS, HeLa S3) were transferred to the complete Leibowitz L-15 medium (10% FCS) and plated to glass bottom wells at densities of 40k and 60k of U-2 OS and HeLaS3 cells, respectively, per well.

On the day of the experiment, the cells were placed into an incubator (37 °C, no additional CO_2_) of the confocal microscope. The cells were gently washed with PBS and treated with 35 μL of 1 μM LON in PBS and incubated for 10 min at 37 °C. After this period, the LON solution was removed and the cells were washed with PBS prior to confocal microscopy imaging was performed at specified time intervals (0 min, 4 h, 24 h). The samples were irradiated with a laser beam set at 639 nm, and the detector (GaAsP) range was set to 640–694 nm. For the two-color experiment the samples were additionally irradiated with a laser beam set at 493 nm, and the detector (GaAsP) range was set to 490–579 nm. Acquisition parameters can be found in Table S2.† The images were taken and exported with Zeiss Imaging Software ZEN 3.2 (blue edition). Images were further processed using ImageJ v1.53t Software for the purpose of two-color quantitative image analysis using the approach detailed in the ESI.†

### Flow cytometry assessment

For flow cytometry experiments the HL-60 cell line was used. The cells were counted using a cell counter (Luna-FL™ Dual Fluorescent Cell Counter, Logos Biosystems, South Korea), washed with PBS and aliquoted (6 × 10^6^ cells per 1.5 mL Eppendorf tube). The pellets were treated with 75 μL of 1 μM LON in PBS for 10 minutes at 37 °C, washed 3× in PBS, resuspended in L-15 media and cultivated in an Eppendorf thermoblock at 37 °C. The fluorescence intensities were measured at several time points (0, 15, 30 min, 1, 2, 4, and 24 h). The sample flow rate was 60 μL min^−1^ and approximately 200 events per s. We collected a minimum of 5000 cells out of the live cell gated population (FSC-A *vs.* SSC-A) and single cell gated population (FSC-A *vs.* FSC-H). A histogram was created for each measurement using a red laser 638 nm with detection filters 660/10 nm band pass (channel R660-APC-A). Data obtained were analyzed using the FlowJo™ v10.6.1 Software (Becton, Dickinson & Company) and exported data were evaluated using GraphPad Prism v.8.4.3. (686) Software (La Jolla, USA).

## Data availability

Data are available in ESI† and upon request from the authors.

## Author contributions

DK, TK and MH conceptualized and designed experiments, analysed data, and wrote the manuscript. DK carried out chemical synthesis and biochemistry, EK and TK did cell experiments and microscopy, RP performed NMR analysis.

## Conflicts of interest

No conflict of interest to declare.

## Supplementary Material

SC-014-D2SC06718H-s001
