# Supplementary material for: Lipid-linked nucleoside triphosphates for enzymatic synthesis of hydrophobic oligonucleotides with enhanced membrane anchoring efficiency
Source: Chem Sci. 2023 Mar 20;14(15):4059–69. doi: 10.1039/d2sc06718h (PMC10094435; doi:10.1039/d2sc06718h)
Supplement: SC-014-D2SC06718H-s001 [file SC-014-D2SC06718H-s001.pdf]

Electronic Supplementary Material (ESI) for Chemical Science.
This journal is © The Royal Society of Chemistry 2023

Supporting Information

Lipid-linked nucleoside triphosphates for enzymatic synthesis of hydrophobic oligonucleotides with enhanced membrane anchoring efficiency

David Kodr,[a+] Erika Kužmová,[a] Radek Pohl,[a] Tomáš Kraus,[a]* and Michal Hocek[a,b]*

a Institute of Organic Chemistry and Biochemistry, Academy of Sciences of the Czech Republic, Flemingovo namesti 2, CZ-16610 Prague 6, Czech Republic;

b Department of Organic Chemistry, Faculty of Science, Charles University in Prague, Hlavova 8, Prague-2 12843, Czech Republic.

tomas.kraus@uochb.cas.cz; hocek@uochb.cas.cz

Table of Contents

1 Experimental section – organic chemistry 4

1.1 Schemes of synthetic approach 5

1.2 Synthesis 10

2 Experimental section – biochemistry 28

2.1 Primer extension experiments 30

2.1.1 Single $dT^{Pam}TP$ incorporation 30

2.1.2 Single $dT^{Pam}TP$ incorporation in the terminal position 30

2.1.3 Multiple $dT^{Pam}TP$ incorporation 30

2.1.4 Single $dC^{Cdeg}TP$ incorporation 31

2.1.5 Single $dC^{Cdeg}TP$ incorporation in the terminal position 32

2.1.6 Multiple $dC^{Cdeg}TP$ incorporation 32

2.1.7 Single $dC^{Calk}TP$ incorporation 33

2.1.8 Single $dC^{Calk}TP$ incorporation in the terminal position 34

2.1.9 Multiple $dC^{Calk}TP$ incorporation 34

2.1.10 Single $dC^{Cest}TP$ incorporation 35

2.1.11 Single $dA^{DPPE}TP$ incorporation 36

2.1.12 Single $dT^{DHPE}TP$ incorporation 37

2.1.13 Single $dT^{DHPE}TP$ incorporation in the terminal position 38

2.2 Polymerase chain reaction (PCR) study with modified dNTPs – example of tested conditions 39

2.3 Terminal deoxyribonuleotidyl transferase (TdT) single strand elongation study with modified dNTPs – 40

2.3.1 Example of tested analytical conditions 40

2.3.1 Example of tested preparative conditions and effect of various additives 41

2.4 Preparation of newly designed probes for improved membrane anchoring 42

2.4.1 Oligonucleotides bearing two modified dT^{Pam} (I.) 42

2.4.2 Oligonucleotides bearing mixed, multiple modifications (II.) 42

2.4.3 Oligonucleotides bearing two modified dC^{Cdeg} (III.) 42

2.5 Ester bond stability assessment 45

2.5.1 Incubation of $dC^{Cest}TP$ 45

2.5.2 Incubation of $dA^{DPPE}TP$ 45

2.6 Magnetoseparation procedure 48

2.7 MALDI-TOF measurements 48

2.8 Cell cultures and cultivation conditions 50

2.9 Live-cell imaging of the anchoring of LONs into cells by confocal microscopy 50

2.10 Cell membrane persistence study of LONs using flow cytometry 53

3. Copies of MALDI-TOF MS spectra 59

4. Copies of NMR spectra 69

5. References 92

1 Experimental section – organic chemistry

General remarks

NMR spectra were recorded on 500 (500.0 or 500.2 MHz for ^{1}H, 125.7 or 125.8 MHz for ^{13}C and 202.4 or 202.5 MHz for ^{31}P) or 600 (600.1 MHz for ^{1}H, 150.9 MHz for ^{13}C) spectrometers from sample solutions in $CDCl_3$, DMSO-d_6, ~~D_2O~~ or CD_3OD. All chemical shifts are quoted on the δ scale in ppm and referenced using residual ^{1}H solvent signal in ^{1}H NMR spectra (δ($CHCl_3$) = 7.26 ppm; δ(CHD_2OD) = 3.31 ppm; δ(CHD_2SO2CD_3) = 2.50 ppm) and ^{13}C solvent signal in ^{13}C NMR spectra (δ($CDCl_3$) = 77.16 ppm; δ(CD_3OD) = 49.0 ppm; δ($CD_3SO_2CD_3$) = 39.52 ppm). ^{31}P chemical shifts were referenced to H_3PO_4 (0 ppm) as external reference. Coupling constants (*J*) are reported in Hz with the following splitting abbreviations: s = singlet, d= doublet, t = triplet, q = quartet, m = multiplet. Complete assignment of all NMR signals was achieved by using a combination of H,H-COSY, H,C-HSQC, and H,C-HMBC experiments. Reactions were monitored by thin layer chromatography (TLC) on TLC Silica gel 60 F_{254} (Merck) and detected by UV (254 nm) or by solution of 4-anisaldehyde in ethanol and 10% of sulphuric acid, or by solution of H_2SO_4 in methanol (10%, v/v). Reactions were monitored by Advion Expression Compact Mass Spectrometer connected with Plate Express® TLC Plate Reader using electrospray ionization (ESI). High performance flash chromatography (HPFC) were performed with ISCO Combiflash Rf system on RediSep Rf Gold Silica Gel Disposable columns or Reverse Phase (C18) RediSep Rf column. Purification of nucleoside triphosphates was performed using HPLC (Waters modular HPLC system) on a column packed with 5 µm C18 reversed phase (Phenomenex, Luna C18 (2) 100 Å, or Kinetex C18 (2) 100 Å). High resolution mass spectra (HR MS) were measured on a LTQ Orbitrap XL (Thermo Fischer Scientific) spectrometer using ESI ionization technique. Chemicals were of analytical grade.

1.1 Schemes of synthetic approach

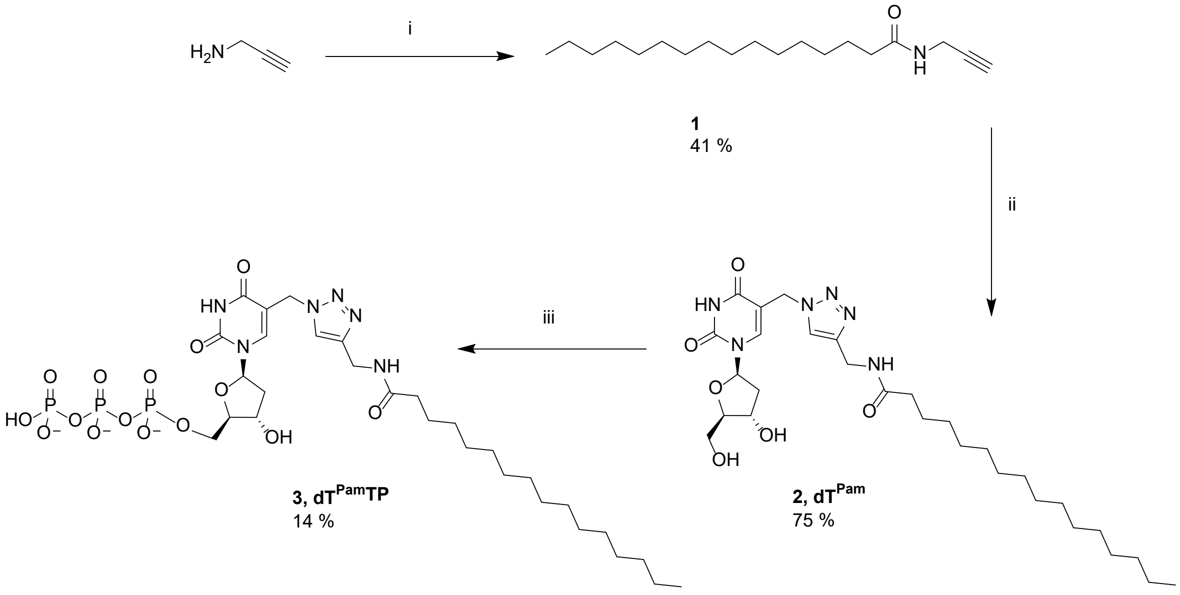

Scheme S1. Synthesis of **dT^{Pam}TP**; Reagents and conditions: i) palmitic acid, DMAP, DCC, dry DCM, 16 h, 22 °C; ii) 5-azidomethy-2'-deoxyuridine, sodium ascorbate, $CuSO_4$ 5$\cdot H_2O$, DMF, 16 h, 22 °C; iii) 1. $POCl_3$, $PO(OMe)_3$, 0 °C, 2 h; 2. pyrophosphate, Bu_3N, DMF, 1 h, 0 °C; 3. TEAB, 22 °C, 15 min.

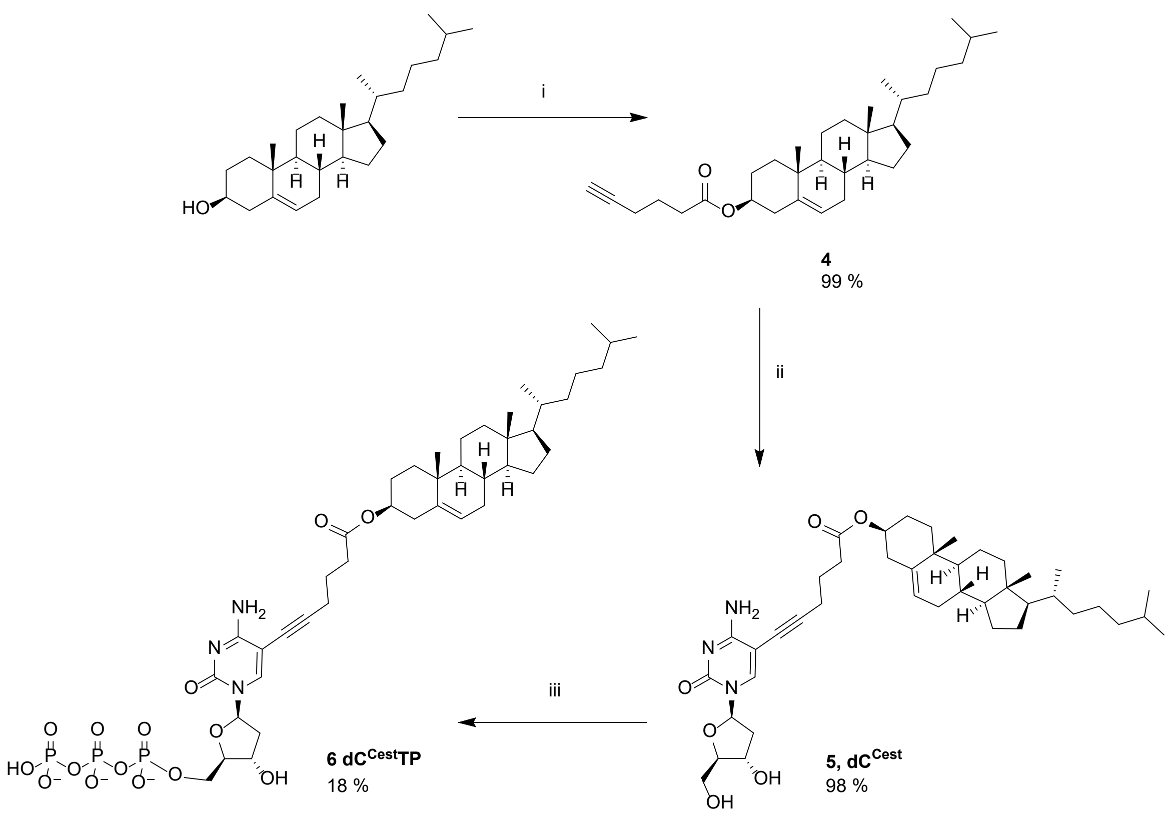

Scheme S2. Synthesis of **dC^{Cest}TP**; Reagents and conditions: i) 4-pentynoic acid, DMAP, DCC, dry DCM, 16 h, 22 °C; ii) 5-iodo-2'-deoxycytidine, $Pd(PPh_3)_4$, CuI, DMF, 75 °C, 2 h; iii) 1. $POCl_3$, $PO(OMe)_3$, 0 °C, 2 h; 2. pyrophosphate, Bu_3N, DMF, 1 h, 0 °C; 3. TEAB, 22 °C, 15 min.

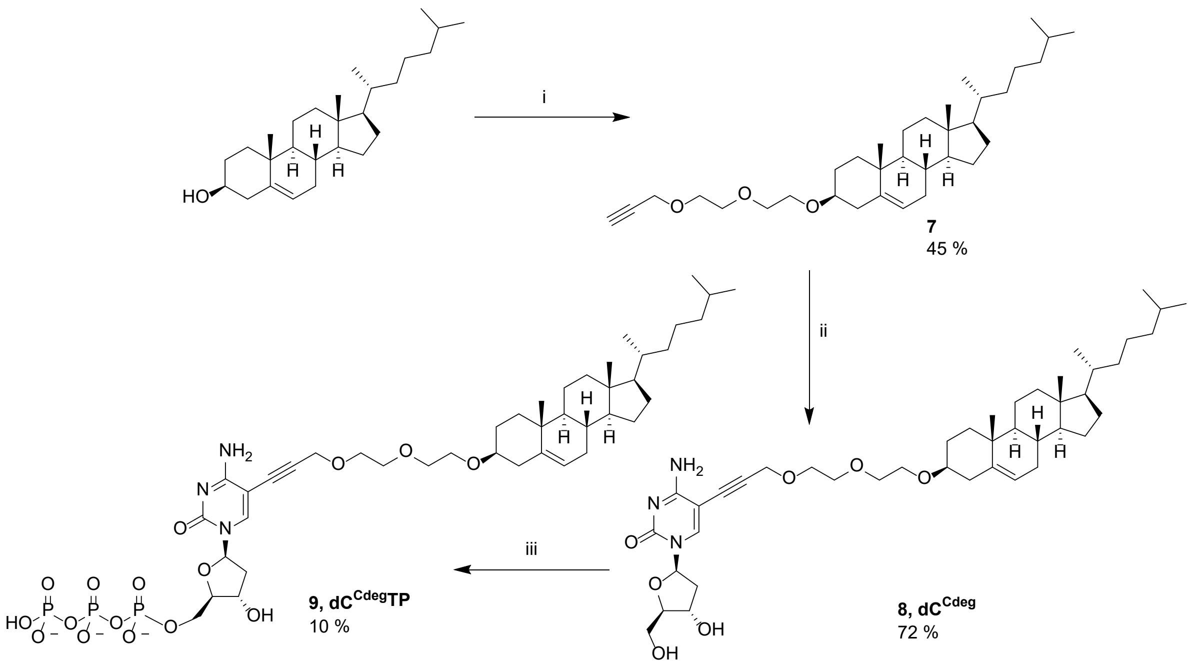

Scheme S3. Synthesis of **dC^Cdeg^TP**; Reagents and conditions: i) *O*-propargyl-diethyleneglycol, Montmorillonite K-10, $CHCl_3$, 20 h, 90 °C (microwave); ii) 5-iodo-2'-deoxycytidine, $Pd(PPh_3)_4$, CuI, DMF, 75 °C, 2 h; iii) 1. $POCl_3$, $PO(OMe)_3$, 0 °C, 2 h; 2. pyrophosphate, Bu_3N, DMF, 1 h, 0 °C; 3. TEAB, 22 °C, 15 min.

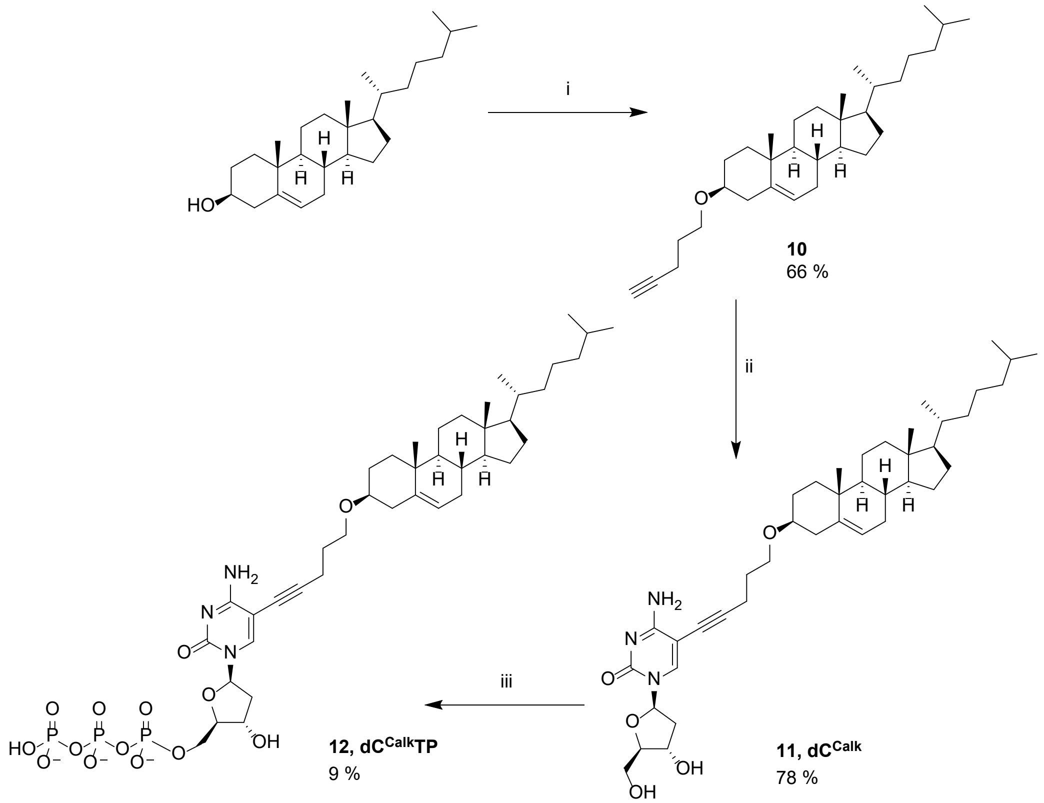

Scheme S4. Synthesis of **dC^{Calk}TP**; Reagents and conditions: i) 4-pentyn-1-ol, Montmorillonite K-10, $CHCl_3$, 20 h, 90 °C (microwave); ii) 5-iodo-2’-deoxycytidine, $Pd(PPh_3)_4$, CuI, DMF, 75 °C, 2 h; iii) 1. $POCl_3$, $PO(OMe)_3$, 0 °C, 2 h; 2. pyrophosphate, Bu_3N, DMF, 1 h, 0 °C; 3. TEAB, 22 °C, 15 min.

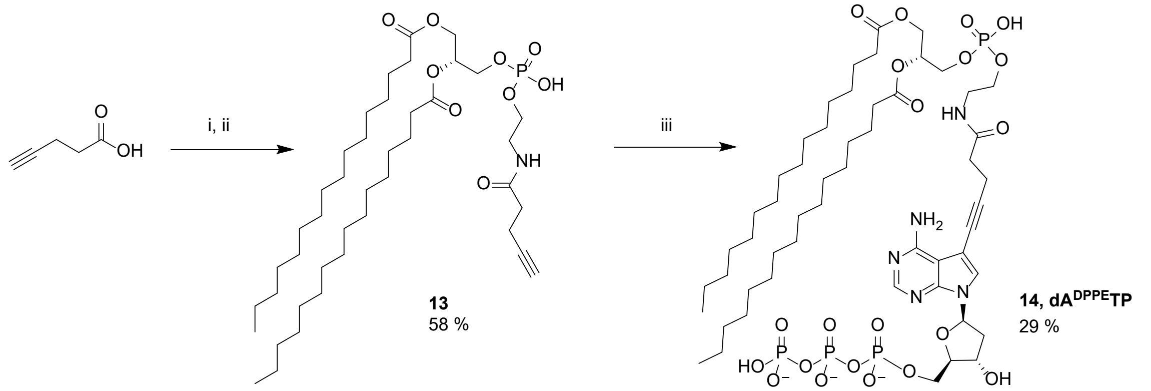

Scheme S5. Synthesis of **dA^{DPPE}TP**; Reagents and conditions: i) NHS, EDCI, dry DCM, 1 h, 22 °C; ii) 1,2-dipalmitoyl-*sn*-glycero-3-phosphoethanolamine, DIPEA, dry DCM, 28 h, 22 °C; iii) 7-deaza-7-iodo-2’-deoxyadenosine triphosphate, $Pd(PPh_3)_2(Cl)_2$, CuI, Et_3N, DMF, 75 °C, 1 h.

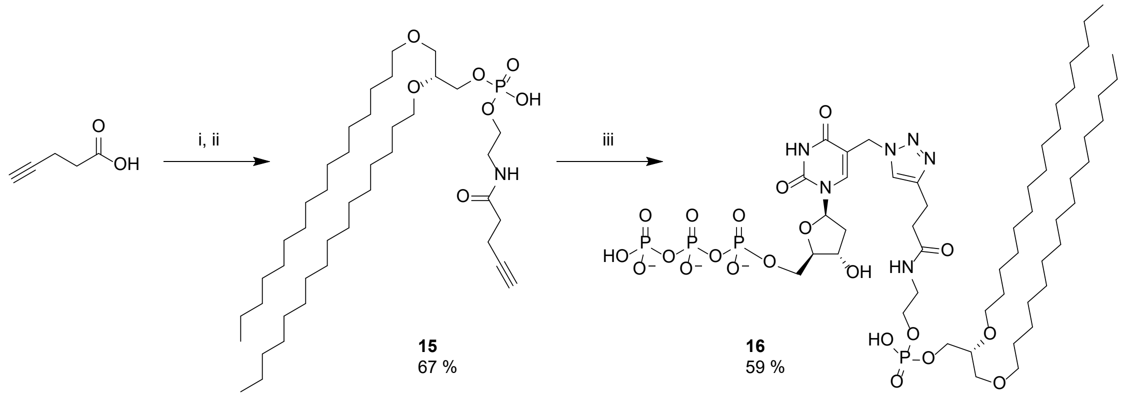

Scheme S6. Synthesis of **dT^{DHPE}TP**; Reagents and conditions: i) NHS, EDCI, dry DCM, 1 h, 22 °C; ii) 1,2-di-*O*-hexadecyl-*sn*-glycero-3-phosphoethanolamine, DIPEA, dry DCM, 1 h, 22 °C; iii) 5-azidomethy-2'-deoxyuridine, sodium ascorbate, $CuSO_4$ 5·H_2O, DMSO, 4 h, 22 °C.

1.2 Synthesis

***N*-Propargylpalmitamide, compound 1**

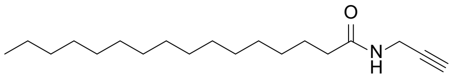

Palmitic acid (698 mg, 2.722 mmol), 4-dimethylaminopyridine (DMAP; 444 mg, 3.630 mmol) and *N*,*N*'-dicyclohexylcarbodiimide (DCC; 749 mg, 3.630 mmol) were dissolved in 15 mL dry DCM in an argon purged flask and 0.12 mL propargyl amine (100 mg, 1.815 mmol) was added through septum. Reaction was stirred for 16 h at 22 °C under argon atmosphere. Reaction was concentrated under vacuo. Crude mixture was purified using normal phase silica gel flash chromatography with toluene-acetone mobile phase (linear gradient). Product was obtained as a white lyophilizate in 41% yield (220 mg). R_f (cyclohexane/AcOEt, 1:1) = 0.32; HR MS (ESI-) for $C_{19}H_{34}NO$ $[M – H]^{1-}$ calcd.: 292.2646 found: 292.2646.

Spectral data were in accordance with literature.[1]

5-{4-[(16-oxo-hexadec-16-yl)amino]methyl-1,2,3-triazole-1-*N*-yl}methyl-2'-deoxyuridine, compound 2, dT^{Pam}

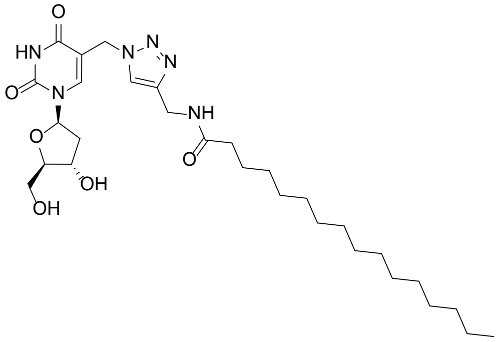

In argon purged flask, 5-azidomethyl-2'-deoxyuridine (70 mg, 0.247 mmol) and *N*-propargylpalmitamide (**1**; 80 mg, 0.272 mmol) were dissolved in DMF (2 mL) and degassed with argon. 1 M water solution of $CuSO_4$ (25 μL) and 1 M water solution of sodium ascorbate (100 μL) were added. Reaction stirred for 16 h at 22 °C under argon atmosphere. Reaction was concentrated under vacuo. Crude mixture was purified using normal phase silica gel flash chromatography with DCM-MeOH mobile phase (linear gradient). Product was obtained as a white lyophilizate in 75% yield (106 mg). R_f (DCM/MeOH, 10:1) = 0.38; HR MS (ESI-) for

$C_{29}H_{47}N_6O_6$ [M – H]$^{1-}$ calcd.: 575.3563 found: 575.3557; ^{1}H NMR (500.2 MHz, DMSO-d_6): 0.82 – 0.89 (m, 3H, C**H$_3$**(CH$_2$)$_{13}$CH$_2$CO); 1.17 – 1.30 (m, 24H, CH$_3$(C**H$_2$**)$_{12}$CH$_2$CH$_2$CO); 1.47 (p, 2H, J_{vic} = 7.3, CH$_3$(CH$_2$)$_{12}$C**H$_2$**CH$_2$CO); 2.04 – 2.16 (m, 4H, H-2′, CH$_3$(CH$_2$)$_{13}$C**H$_2$**CO); 3.54, 3.59 (2 × ddd, 2 × 1H, J_{gem} = 11.8, $J_{5',OH}$ = 5.2, $J_{5',4'}$ = 4.1); 3.78 (td, 1H, $J_{4',5'}$ = 4.1, $J_{4',3'}$ = 3.0, H-4′); 4.23 (m, 1H, H-3′); 4.24 (d, 2H, J_{vic} = 5.6, C**H$_2$**NH); 5.03 (t, 1H, $J_{OH,5'}$ = 5.5, OH-5′); 5.13, 5.17 (2 × d, 2 × 1H, J_{gem} = 14.7, CH$_2$N); 5.26 (d, 1H, $J_{OH,3'}$ = 4.3, OH-3′); 6.13 (dd, 1H, $J_{1',2'}$ = 7.2, 6.2, H-1′); 7.81 (s, 1H, H-5-triazole); 8.12 (s, 1H, H-6); 8.22 (t, 1H, J_{vic} = 5.6, CH$_2$N**H**); 11.55 (s, 1H, NH-3). ^{13}C NMR (125.8 MHz, DMSO-d_6): 14.12 (**C**H$_3$(CH$_2$)$_{13}$CH$_2$CO); 22.25 (CH$_3$(**C**H$_2$)$_{12}$CH$_2$CH$_2$CO); 25.36 (CH$_3$(CH$_2$)$_{12}$**C**H$_2$CH$_2$CO); 28.85, 28.86, 28.93, 29.09, 29.17, 29.18, 29.21, 31.45 (CH$_3$(**C**H$_2$)$_{12}$CH$_2$CH$_2$CO); 34.23 (CH$_2$NH); 35.36 (CH$_3$(CH$_2$)$_{12}$CH$_2$**C**H$_2$CO); 39.87 (CH$_2$-2′); 46.17 (CH$_2$N); 61.38(CH$_2$-5′); 70.36 (CH-3′); 84.62 (CH-1′); 87.69 (CH-4′); 108.02 (C-5); 122.85 (CH-5-triazole); 141.05 (CH-6); 145.07 (C-4-triazole); 150.33 (C-2); 162.74 (C-4); 172.25 (CO).

5-{4-[(16-oxo-hexadec-16-yl)amino]methyl-1,2,3-triazole-1-*N*-yl}methyl-2′-deoxyuridine 5′-*O*-triphosphate, compound 3, dT^{Pam}TP

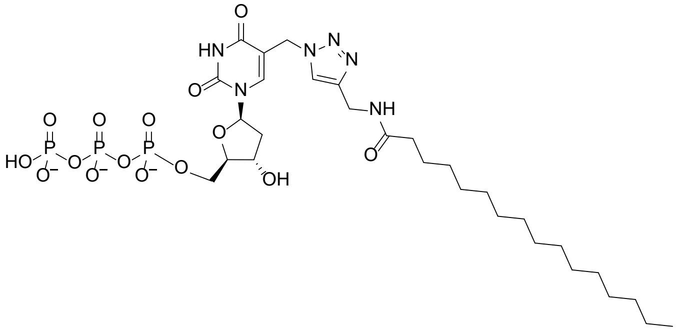

dT^{Pam} (**2**; 30 mg, 0.052 mmol) was stirred under vacuo for 16 h at 65 °C. Then it was dissolved in dry PO(OMe)$_3$ (0.5 mL) under argon atmosphere and cooled on ice-salt bath. POCl$_3$ (6 μL) was added through septum and stirred for 2 h at 0 °C. In another argon purged flask, bis(tributylammonium) pyrophosphate (143 mg, 0.260 mmol) was dissolved in dry DMF (0.5 mL) and after subsequent addition of Bu$_3$N (50 μL) and cooling down on ice-salt bath was mixture added to the reaction. Reaction was stirred for additional 1 h at 0 °C. 2 M TEAB (0.5 mL) was added dropwise and stirred for 15 min at 22 °C. Reaction was concentrated under vacuo. Product was purified on RP-HPLC with use of a linear gradient of 0.1 M TEAB (triethylammonium

bicarbonate) in H_2O to 0.1 M TEAB in H_2O/MeOH (1:1) to MeOH as eluent in 90 minutes. Several co-distillations with water and subsequent lyophilization gave product in form of Et_3NH^+ salt as a white gummy solid in 14% yield (8 mg). HR MS (ESI-) for $C_{29}H_{50}N_6O_{15}P_3$ $[M – H]^{1-}$ calcd.: 815.2552 found: 815.2528; ^{1}H NMR (500.0 MHz, CD_3OD): 0.90 (t, 3H, J_{vic} = 7.1, C**H3**(CH2)13CH2CO); 1.25 – 1.36 (m, 51H, CH3(C**H2**)12CH2CH2CO, C**H3**CH2N); 1.55 – 1.63 (m, 2H, CH3(CH2)12C**H2**CH2CO); 2.19 – 2.23 (m, 2H, CH3(CH2)13C**H2**CO); 2.31 (ddd, 1H, J_{gem} = 13.6, $J_{2'b,1'}$ = 6.3, $J_{2'b,3'}$ = 4.6, H-2'b); 2.37 (dt, 1H, J_{gem} = 13.6, $J_{2'a,1'}$ = $J_{2'a,3'}$ = 6.3, H-2'a); 3.13 – 3.25 (bm, 18H, CH3C**H2**N); 4.03 (dddd, 1H, $J_{4',3'}$ = 3.7, $J_{4',5'}$ = 2.9, 2.5, $J_{H,P}$ = 2.7, H-4'); 4.29 (ddd, 1H, J_{gem} = 11.5, $J_{H,P}$ = 4.5, $J_{5'b,4'}$ = 2.9, H-5'b); 4.35 (ddd, 1H, J_{gem} = 11.5, $J_{H,P}$ = 5.9, $J_{5'a,4'}$ = 2.5, H-5'a); 4.43 (s, 2H, C**H2**NH); 4.68 (m, 1H, H-3'); 5.40, 5.43 (2 × d, 2 × 1H, J_{gem} = 14.5, CH2N); 6.28 (t, 1H, $J_{1',2'}$ = 6.3, H-1'); 8.08 (s, 1H, H-5-triazole); 8.48 (s, 1H, H-6). ^{13}C NMR (125.7 MHz, CD_3OD): 9.12 (**C**H3CH2N); 14.44 (**C**H3(CH2)13CH2CO); 23.73 (CH3(**C**H2)12CH2CH2CO); 26.95 (CH3(CH2)12**C**H2CH2CO); 30.38, 30.47, 30.50, 30.64, 30.75, 30.76, 30.78, 30.79, 30.80, 33.07 (CH3(**C**H2)12CH2CH2CO); 35.62 (CH2NH); 36.98 (CH3(CH2)12CH2**C**H2CO); 41.18 (CH2-2'); 47.38 (CH3**C**H2N); 47.89 (CH2N); 66.31 (d, $J_{C,P}$ = 5.7, CH2-5'); 71.42 (CH-3'); 86.70 (CH-1'); 87.79 (d, $J_{C,P}$ = 9.1, CH-4'); 109.62 (C-5); 124.73 (CH-5-triazole); 143.15 (CH-6); 145.82 (C-4-triazole); 152.11 (C-2); 164.74 (C-4); 176.09 (CO). ^{31}P NMR (202.4 MHz, CD_3OD): -22.32 (t, J = 21.6, Pβ); -10.07 (d, J = 21.6, Pα); -9.08 (d, J = 21.6, Pγ).

Cholesteryl 5-hexynate, compound 4

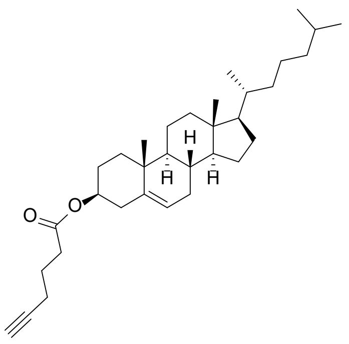

In argon purged flask, cholesterol (1.00 g, 2.586 mmol), DMAP (632 mg, 5.172 mmol) and DCC (1.07 g, 5.172 mmol) were dissolved in dry DCM (20 mL) and 0.43 ml of 5-hexynoic acid (3.879 mmol) was added. Reaction was stirred for 16 h at 22 °C. Mixture was filtered and concentrated under vacuo. Product was purified using normal phase silica gel flash

chromatography with toluene-acetone mobile phase (linear gradient). Product was obtained as a white lyophilizate in 99% yield (1.23 g). R_f (cyclohexane/AcOEt, 3:1) = 0.85; HR MS (ESI+) for $C_{33}H_{52}O_2Na$ [M + Na]$^{1-}$ calcd.: 503.3860 found: 503.3861.

Spectral data were in accordance with literature.[2]

5-(*O*-Cholesteryl-5-carboxy-1-pentynyl)-2'-deoxycytidine, compound 5, dC^{Cest}

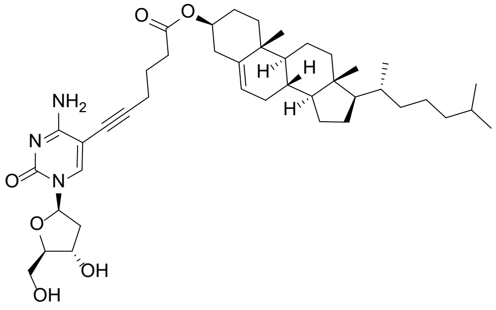

In argon purged flask 2'-deoxy-5-iodocytidine (100 mg, 0.283 mmol), cholesteryl 5-hexynate (**4**; 272 mg, 0.566 mmol), $Pd(PPh_3)_4$ (33 mg, 0.028 mmol) and CuI (11 mg, 0.057 mmol) were dissolved in dry DMF (1.5 mL) and 0.1 mL of Et_3N was added. Reaction was stirred for 2 h at 75 °C. Reaction was concentrated under vacuo and residual DMF was removed by several co-distillations with toluene. Crude mixture was purified using normal phase silica gel flash chromatography with DCM-MeOH mobile phase (linear gradient). Product was obtained as s white lyophilizate in 98% yield (195 mg). R_f (DCM/MeOH, 7:1) = 0.45 HR MS (ESI-) for $C_{42}H_{63}N_3O_6Na$ [M + Na]$^{1+}$ calcd.: 728.4609 found: 728.4605; ^{1}H NMR (600.1 MHz, DMSO-d_6): 0.65 (s, 3H, CH_3-18-chol); 0.838, 0.842 (2 × d, 2 × 3H, $J_{26\&27,25}$ = 6.6, CH_3-26,27-chol); 0.89 (d, 3H, $J_{21,20}$ = 6.5, CH_3-21-chol); 0.92 (m, 1H, H-9-chol); 0.97 (s, 3H, CH_3-19-chol); 0.97 – 1.17 (m, 9H, H-1α,12α,14,15α,17,22b,23b,24-chol); 1.24 (m, 1H, H-16β–chol); 1.28 – 1.43 (m, 5H, H-8,11b,20,22a,23a-chol); 1.45 – 1.57 (m, 5H, H-2α,7α,11a,15β,25-chol); 1.74 – 1.85 (m, 5H, H-1β,2α,16α-chol, $OCCH_2C\mathbf{H_2}CH_2C{\equiv}C$); 1.91 (m, 1H, H-7β-chol); 1.93 – 2.00 (m, 2H, H-2'b,H-12β-chol); 2.12 (ddd, 1H, J_{gem} = 13.2, $J_{2'a,1'}$ = 6.0, $J_{2'a,3'}$ = 3.5, H-2'a); 2.26 (d, 2H, $J_{4,3}$ = 7.6, H-4-chol); 2.39 (t, 2H, J_{vic} = 7.3, $OCC\mathbf{H_2}CH_2CH_2C{\equiv}C$); 2.44 (t, 2H, J_{vic} = 7.1, $OCCH_2CH_2C\mathbf{H_2}C{\equiv}C$); 3.52 – 3.62 (m, 2H, H-5'); 3.78 (q, 1H, $J_{4',3'} = J_{4',5'}$ = 3.5, H-4'); 4.19 (bm, 1H, H-3'); 47 (m, 1H, H-3-chol); 5.04 (bs, 1H, OH-5'); 5.19 (bs, 1H, OH-3'); 5.32 (m, 1H, H-6-chol); 6.10 (dd, 1H, $J_{1',2'}$ = 7.3, 6.0, H-1'); 6.76,

7.73 (2 × bs, 2 × 1H, NH_2); 8.08 (s, 1H, H-6). ^{13}C NMR (150.9 MHz, DMSO-d_6): 11.68 (CH_3-18-chol); 18.53 (C≡**C**$CH_2CH_2CH_2$CO); 18.56 (CH_3-21-chol); 18.97 (CH_3-19-chol); 20.56 (CH_2-11-chol); 22.41, 22.68 (CH_3-26,27-chol); 23.20 (CH_2-23-chol); 23.45 (C≡CCH_2**C**H_2CH_2CO); 23.87 (CH_2-15-chol); 27.36 (CH_2-2-chol); 27.40 (CH-25-chol); 27.79 (CH_2-16-chol); 31.33 (CH_2-7-chol); 31.37 (CH-8-chol); 32.94 (C≡CCH_2CH_2**C**H_2CO); 35.20 (CH-20-chol); 35.86 (CH_2-22-chol); 36.09 (C-10-chol); 36.50 (CH_2-1-chol); 37.69 (CH_2-4-chol); 38.94 (CH_2-24-chol); 39.20 (CH_2-12-chol); 40.71 (CH_2-2′); 41.86 (C-13-chol); 49.43 (CH-9-chol); 55.57 (CH-17-chol); 56.12 (CH-14-chol); 61.05 (CH_2-5′); 70.17 (CH-3′); 72.67 (**C**≡C$CH_2CH_2CH_2$CO); 73.26 (CH-3-chol); 85.35 (CH-1′); 87.43 (CH-4′); 90.94 (C-5); 94.74 (C≡**C**$CH_2CH_2CH_2$CO); 122.10 (CH-6-chol); 139.48 (C-5-chol); 143.69 (CH-6); 153.31 (C-2); 164.30 (C-4); 172.03 (C≡C$CH_2CH_2CH_2$**C**O).

5-(*O*-Cholesteryl-5-carboxy-1-pentynyl)-2′-deoxycytidine 5′-*O*-triphosphate, compound 6, dC^{Cest}TP

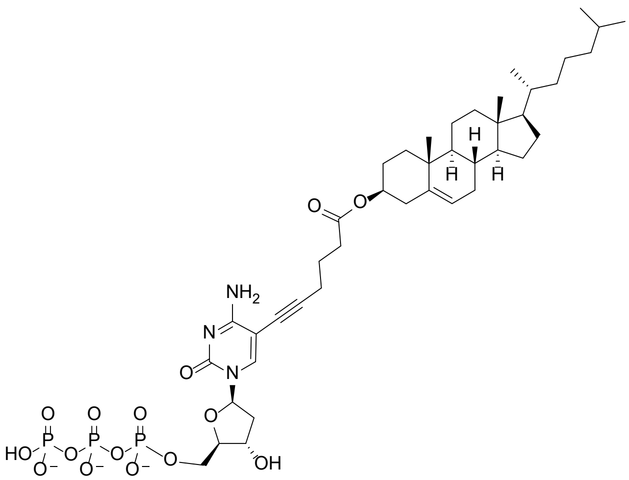

dC^{Cest} (**5**; 50 mg, 0.071 mmol) was stirred under vacuo for 16 h at 65 °C. Then it was dissolved in dry $PO(OMe)_3$ (0.5 mL) under argon atmosphere and cooled on ice-salt bath. $POCl_3$ (8 μL) was added through septum and stirred for 2 h at 0 °C. In another argon purged flask, bis(tributylammonium) pyrophosphate (195 mg, 0.355 mmol) was dissolved in dry DMF (0.5 mL) and after subsequent addition of Bu_3N (68 μL) and cooling down on ice-salt bath was mixture added to the reaction. Reaction was stirred for additional 1 h at 0 °C. 2 M TEAB (0.5 mL) was added dropwise, and stirred for 15 min at 22 °C. Reaction was concentrated under vacuo. Product was purified on RP-HPLC with use of a linear gradient of 0.1 M TEAB (triethylammonium

bicarbonate) in H_2O to 0.1 M TEAB in H_2O/MeOH (1:1) to MeOH as eluent in 90 minutes. Several co-distillations with water and subsequent lyophilization gave product in form of Et_3NH^+ salt as a white gummy solid in 18% yield (15 mg). HR MS (ESI-) for $C_{42}H_{65}N_3O_{15}P_3$ $[M-H]^{1-}$ calcd.: 944.3634 found: 944.3615 ^{1}H NMR (600.1 MHz, CD_3OD): 0.72 (s, 3H, CH_3-18-chol); 0.882, 0.885 (2 × d, 2 × 3H, $J_{26\&27,25}$ = 6.6, CH_3-26,27-chol); 0.95 (d, 3H, $J_{21,20}$ = 6.6, CH_3-21-chol); 0.97 (m, 1H, H-9-chol); 0.99 – 1.07 (s, 5H, H-14,22b-chol, CH_3-19-chol); 1.08 – 1.23 (m, 7H, H-1α,12α,15α,17,23b,24-chol); 1.30 (m, 1H, H-16β–chol); 1.31 (t, 18H, J_{vic} = 7.2, C**H₃**CH₂N); 1.35 – 1.43 (m, 3H, H-20,22a,23a-chol); 1.58 (m, 8H, H-2,7α,8,11,15β,25-chol); 1.82 – 1.94 (m, 4H, H-1β,16α-chol, OCH₂C**H₂**CH₂C≡C); 1.98 (m, 1H, H-7β-chol); 2.05 (dt, 1H, J_{gem} = 12.3, $J_{12\beta,11}$ = 3.4, H-12β-chol); 2.19 (m, 1H, J_{gem} = 13.8, $J_{2'b,1'}$ = 7.2, $J_{2'b,3'}$ = 6.4, H-2′b); 2.28 – 2.33 (m, 2H, H-4-chol); 2.34 (ddd, 1H, J_{gem} = 13.8, $J_{2'a,1'}$ = 6.0, $J_{2'a,3'}$ = 3.6, H-2′a); 2.45 (t, 2H, J_{vic} = 7.2, OCC**H₂**CH₂CH₂C≡C); 2.53 (t, 2H, J_{vic} = 7.1, OCCH₂CH₂C**H₂**C≡C); 3.20 (bq, 12H, J_{vic} = 7.2, CH₃C**H₂**N); 4.08 (m, 1H, H-4′); 4.22 (ddd, 1H, J_{gem} = 11.2, $J_{H,P}$ = 5.4, $J_{5'b,4'}$ = 4.3, H-5′b); 4.28 (ddd, 1H, J_{gem} = 11.2, $J_{H,P}$ = 7.1, $J_{5'a,4'}$ = 3.9, H-5′a); 4.51 – 4.57 (m, 2H, H-3′, H-3-chol); 5.36 (dt, 1H, $J_{6,7\beta}$ = 5.5, $J_{6,4\beta}$ = $J_{6,7\alpha}$ = 2.0, H-6-chol); 6.21 (dd, 1H, $J_{1',2'}$ = 7.2, 6.0, H-1′); 8.06 (s, 1H, H-6). ^{13}C NMR (150.9 MHz, CD_3OD): 9.12 (**C**H₃CH₂N); 12.32 (CH_3-18-chol); 19.26 (CH_3-21-chol); 19.78 (CH_3-19-chol); 19.88 (C≡C**C**H₂CH₂CH₂CO); 22.14 (CH_2-11-chol); 22.95, 23.20 (CH_3-26,27-chol); 24.82 (C≡CCH₂**C**H₂CH₂CO); 24.98 (CH_2-23-chol); 25.31 (CH_2-15-chol); 28.66 (CH_2-2-chol); 29.15 (CH-25-chol); 29.34 (CH_2-16-chol); 31.02 (CH_2-7-chol); 33.20 (CH-8-chol); 34.56 (C≡CCH₂CH₂**C**H₂CO); 37.15 (CH-20-chol); 37.40 (CH_2-22-chol); 37.76 (C-10-chol); 38.27 (CH_2-1-chol); 39.22 (CH_2-4-chol); 40.70 (CH_2-24-chol); 41.13 (CH_2-12-chol); 41.30 (CH_2-2′); 43.51 (C-13-chol); 47.43 (CH₃**C**H₂N); 51.60 (CH-9-chol); 57.59 (CH-17-chol); 58.13 (CH-14-chol); 66.55 (d, $J_{C,P}$ = 4.7, CH_2-5′); 71.88 (CH-3′); 71.91 (**C**≡CCH₂CH₂CH₂CO); 75.46 (CH-3-chol); 87.60 (CH-1′); 87.63 (d, $J_{C,P}$ = 9.1, CH-4′); 93.63 (C-5); 97.50 (C≡**C**CH₂CH₂CH₂CO); 123.61 (CH-6-chol); 141.07 (C-5-chol); 145.22 (CH-6); 154.59 (C-2); 165.09 (C-4); 174.52 (C≡CCH₂CH₂CH₂**C**O).

Cholesteryl (2-*O*-Propargyl-2-oxyethoxy)ethyl ether, compound 7

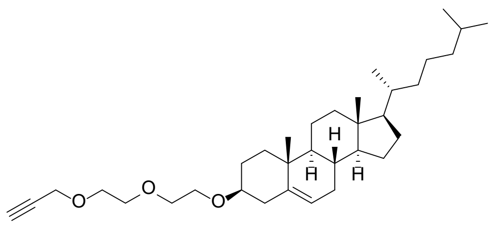

Montmorillonite K-10 clay (1 g) was activated at 120 °C under vacuo for 16 h. Then it was suspended in dry $CHCl_3$ (5 mL) and cholesterol (500 mg, 1.293 mmol) and *O*-propargyl-diethyleneglycol (932 mg, 6.465 mmol) were added. Suspension was stirred for 20 h at 90 °C in microwave reactor, diluted by 25 mL of $CHCl_3$ and filtered, concentrated under vacuo and purified using normal phase silica gel flash chromatography with cyclohexane-AcOEt mobile phase (linear gradient). Product was obtained as a white lyophilizate in 45% yield (300 mg). R_f (cyclohexane/AcOEt, 3:1) = 0.50; HR MS (ESI+) for $C_{34}H_{56}O_3Na$ $[M + Na]^{1+}$ calcd.: 535.4122 found: 535.4124; ^1H NMR (500.0 MHz, $CDCl_3$): 0.67 (s, 3H, CH_3-18); 0.858, 0.863 (2 × d, 2 × 3H, $J_{26\&27,25}$ = 6.6, CH_3-26,27); 0.91 (d, 3H, $J_{21,20}$ = 6.6, CH_3-21); 0.92 (m, 1H, H-9); 0.985 (m, 1H, H-14); 0.99 (m, 1H, H-22b); 0.99 (s, 3H, CH_3-19); 0.99 – 1.20 (m, 7H, H-1α,12α,15α,17,23b,24); 1.25 (m, 1H, H-16β); 1.30 – 1.39 (m, 3H, H-20,22a,23a); 1.40 – 1.60 (m, 7H, H-2β,7α,8,11,15β,25); 1.82 (m, 1H, H-16α); 1.84 (m, 1H, H-1β); 1.90 (m, 1H, H-2α); 1.96 (m, 1H, H-7β); 2.00 (m, 1H, H-12β); 2.21 (m, 1H, H-4β); 2.37 (ddd, 1H, $J_{4\alpha,4\beta}$ = 13.2, $J_{4\alpha,3}$ = 4.6, $J_{4\alpha,2\alpha}$ = 2.4, H-4α); 2.42 (t, 1H, ^4J = 2.4, **H**C≡CCH_2O); 3.18 (tt, 1H, $J_{3,2\beta} = J_{3,4\beta}$ = 11.3, $J_{3,2\alpha} = J_{3,4\alpha}$ = 4.6, H-3); 3.62 – 3.66, 3.67 – 3.72 (2 × m, 2 × 4H, OCH_2CH_2O); 4.21 (d, 2H, ^4J = 2.4, HC≡CC**H₂**O); 5.34 (dt, 1H, $J_{6,7\beta}$ = 5.3, $J_{6,4\beta} = J_{6,7\alpha}$ = 2.1, H-6). ^{13}C NMR (125.7 MHz, $CDCl_3$): 11.84 (CH_3-18); 18.70 (CH_3-21); 19.37 (CH_3-19); 21.05 (CH_2-11); 22.55, 22.81 (CH_3-26,27); 23.80 (CH_2-23); 24.27 (CH_2-15); 28.00 (CH-25); 28.22 (CH_2-16); 28.34 (CH_2-2); 31.88 (CH-8); 31.93 (CH_2-7); 35.77 (CH-20); 36.17 (CH_2-22); 36.85 (C-10); 37.23 (CH_2-1); 39.05 (CH_2-4); 39.50 (CH_2-24); 39.77 (CH_2-12); 42.30 (C-13); 50.16 (CH-9); 56.13 (CH-17); 56.76 (CH-14); 58.41 (**C**H₂C≡CH); 67.28, 69.12, 70.44, 70.92 (O**C**H₂**C**H₂O); 74.45 (H**C**≡C); 79.49 (CH-3); 79.67 (HC≡**C**); 121.53 (CH-6); 140.97 (C-5).

5-[(2-*O*-Cholesteryl-2-oxyethoxy)ethoxy-1-propynyl]-2′-deoxycytidine, compound 8, dC^{Cdeg}

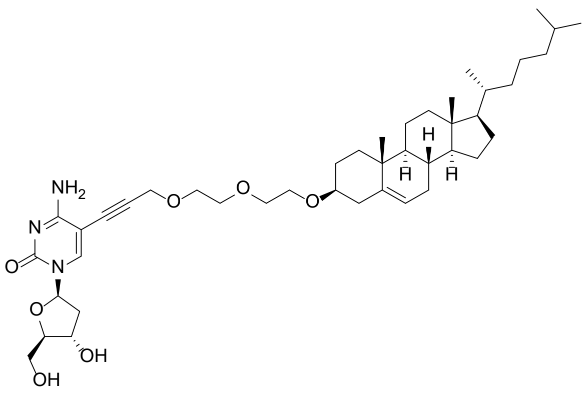

In argon purged flask 2′-deoxy-5-iodocytidine (86 mg, 0.244 mmol), cholesteryl (2-*O*-propargyl-2-oxyethoxy)ethyl ether (**7**; 150 mg, 0.293 mmol), $Pd(PPh_3)_4$ (28 mg, 0.024 mmol) and CuI (9 mg, 0.049 mmol) were dissolved in dry DMF (1.5 mL) and 80 μL of Et_3N was added. Reaction was stirred for 2 h at 75 °C, concentrated under vacuo and residual DMF was removed by several co-distillations with toluene. Crude mixture was purified using normal phase silica gel flash chromatography with DCM-MeOH mobile phase (linear gradient). Product was obtained as a white lyophilizate in 72% yield (129 mg). R_f (DCM/MeOH, 7:1) = 0.70; HR MS (ESI-) for $C_{43}H_{68}N_3O_7$ [M + H]$^{1+}$ calcd.: 738.5052 found: 738.5051; ^1H NMR (500.0 MHz, $CDCl_3$): 0.67 (s, 3H, CH_3-18-chol); 0.859, 0.862 (2 × d, 2 × 3H, $J_{26\&27,25}$ = 6.6, CH_3-26,27-chol); 0.90 (m, 1H, H-9-chol); 0.91 (d, 3H, $J_{21,20}$ = 6.5, CH_3-21-chol); 0.95 – 1.03 (m, 7H, H-1α,14,22b-chol, CH_3-19-chol); 1.03 – 1.18 (m, 6H, H-12α,15α,17,23b,24-chol); 1.25 (m, 1H, H-16β–chol); 1.30 – 1.39 (m, 3H, H-20,22a,23a-chol); 1.40 – 1.54 (m, 6H, H-2β,7α,8,11,25-chol); 1.57 (m, 1H, 15β); 1.77 – 1.92 (m, 3H, H-1β,2α,16α-chol); 1.96 (m, 1H, H-7β-chol); 2.00 (m, 1H, H-12β-chol); 2.19 (m, 1H, H-4β-chol); 2.28 (bm, 1H, H-2′); 2.35 (m, 1H, H-4α-chol); 2.47 (bm, 1H, H-2′a); 3.17 (m, 1H, H-3-chol); 3.61 – 3.65, 3.67 – 3.73 (2 × m, 2 × 4H, OC**H₂**C**H₂**O); 3.85 – 3.93 (bm, 2H, H-5′); 3.99 (m, 1H, H-4′); 4.40 (s, 2H, $OCH_2C{\equiv}C$); 4.50 (m, 1H, H-3′); 5.32 (dt, 1H, $J_{6,7\beta}$ = 5.5, $J_{6,4\beta}$ = $J_{6,7\alpha}$ = 2.0, H-6-chol); 6.08 (bt, 1H, $J_{1',2'}$ = 5.8, H-1′); 6.15, 6.91 (2 × bs, 2 × 1H, NH_2); 8.33 (s, 1H, H-6). ^{13}C NMR (125.7 MHz, $CDCl_3$): 12.02 (CH_3-18-chol); 18.87 (CH_3-21-chol); 19.54 (CH_3-19-chol); 21.22 (CH_2-11-chol); 22.71, 22.97 (CH_3-26,27-chol); 24.01 (CH_2-23-chol); 24.44 (CH_2-15-chol); 28.16 (CH-25-chol); 28.39 (CH_2-16-chol); 28.48 (CH_2-2-chol); 32.02 (CH-8-chol); 32.09 (CH_2-7-chol); 35.95 (CH-20-chol); 36.44 (CH_2-22-chol); 37.00 (C-10-chol); 37.34 (CH_2-1-chol); 39.15 (CH_2-4-chol); 39.66 (CH_2-24-chol);

39.92 (CH_2-12-chol); 41.18 (CH_2-2′); 42.46 (C-13-chol); 50.30 (CH-9-chol); 56.32 (CH-17-chol); 56.90 (CH-14-chol); 59.33 (C≡**C**CH_2O); 61.25 (CH_2-5′); 67.27, 69.45 (OCH_2CH_2O); 69.70 (CH-3′); 70.57, 70.99 (OCH_2CH_2O); 77.63 (**C**≡CCH_2O); 79.73 (CH-3-chol); 87.55 (CH-1′,4′); 90.46 (C-5); 91.70 (C≡**C**CH_2O); 121.90 (CH-6-chol); 140.87 (C-5-chol); 145.89 (CH-6); 154.72 (C-2); 164.47 (C-4).

5-[(2-*O*-Cholesteryl-2-oxyethoxy)ethoxy-1-propynyl]-2′-deoxycytidine 5′-*O*-triphosphate, compound 9, dC$^{\mathbf{Cdeg}}$TP

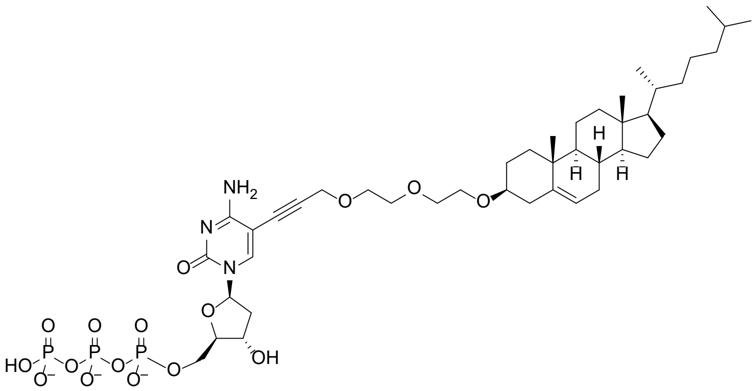

dC$^{\mathbf{Cdeg}}$ (**8**; 90 mg, 0.122 mmol) was stirred under vacuo for 16 h at 65 °C. Then it was dissolved in dry $PO(OMe)_3$ (0.7 mL) under argon atmosphere and cooled on ice-salt bath. $POCl_3$ (14 μL) was added through septum and stirred for 2 h at 0 °C. In another argon purged flask bis(tributylammonium) pyrophosphate (334 mg, 0.610 mmol) was dissolved in dry DMF (0.5 mL) and after subsequent addition of Bu_3N (120 μL) and cooling down on ice-salt bath was mixture added to the reaction. Reaction was stirred for additional 1 h at 0 °C. 2 M TEAB (0.5 mL) was added dropwise and stirred for 15 min at 22 °C. Reaction was concentrated under vacuo. Product was purified on RP-HPLC with use of a linear gradient of 0.1 M TEAB (triethylammonium bicarbonate) in H_2O to 0.1 M TEAB in H_2O/MeCN (1:1) to MeCN as eluent in 90 minutes. Several co-distillations with water and subsequent lyophilization gave product in form of Et_3NH^+ salt as a white gummy solid in 10 % yield (15 mg). HR MS (ESI-) for $C_{43}H_{69}N_3O_{16}P_3$ $[M-H]^{1-}$ calcd.: 976,3896 found: 976,3879; ^{1}H NMR (500.0 MHz, CD_3OD): 0.71 (s, 3H, CH_3-18-chol); 0.880, 0.883 (2 × d, 2 × 3H, $J_{26\&27,25}$ = 6.6, CH_3-26,27-chol); 0.93 (m, 1H, H-9-chol); 0.94 (d, 3H, $J_{21,20}$ = 6.5, CH_3-21-chol); 1.00 (s, 3H, CH_3-19-chol); 1.01 (m, 1H, H-14); 1.02 (m, 1H, H-22b); 1.04 (m, 1H, H-1α); 1.09 – 1.21 (m, 6H, H-12α,15α,17,23b,24-chol); 1.29 (m, 1H, H-16β–chol); 1.31 (t, 18H, J_{vic} = 7.2, C**H₃**CH_2N);

1.36 – 1.42 (m, 3H, H-20,22a,23a-chol); 1.43 (m, 1H, H-2β); 1.44 – 1.56 (m, 5H, H-7α,8,11,25-chol); 1.60 (m, 1H, 15β); 1.80 – 1.94 (m, 3H, H-1β,2α,16α-chol); 1.96 (m, 1H, H-7β-chol); 2.04 (m, 1H, H-12β-chol); 2.16 (m, 1H, H-4β-chol); 2.20 (bm, 1H, H-2′); 2.36 (m, 1H, H-4α-chol); 2.37 (bm, 1H, H-2′a); 3.16 – 3.23 (m, 13H, H-3-chol, CH_3CH_2N); 3.60 – 3.66, 3.67 – 3.70, 3.73 – 3.77 (3 × m, 8H, OC**H₂**C**H₂**O); 4.07 (qd, 1H, $J_{4',3'} = J_{4',5'} = 3.6$, $J_{H,P} = 1.5$, H-4′); 4.23 (ddd, 1H, $J_{gem} = 11.4$, $J_{H,P} = 5.2$, $J_{5'b,4'} = 3.6$, H-5′b); 4.28 (ddd, 1H, $J_{gem} = 11.4$, $J_{H,P} = 6.9$, $J_{5'a,4'} = 3.6$, H-5′b); 4.46 (s, 2H, $OCH_2C≡C$); 4.56 (dt, 1H, $J_{3',2'} = 6.9, 3.6$, $J_{3',4'} = 3.6$, H-3′); 5.34 (dt, 1H, $J_{6,7\beta} = 5.5$, $J_{6,4\beta} = J_{6,7\alpha} = 2.0$, H-6-chol); 6.2a (dd, 1H, $J_{1',2'} = 7.1, 5.9$, H-1′); 8.19 (s, 1H, H-6). ^{13}C NMR (125.7 MHz, CD_3OD): 9.14 (**C**H_3CH_2N); 12.32 (CH_3-18-chol); 19.25 (CH_3-21-chol); 19.86 (CH_3-19-chol); 22.17 (CH_2-11-chol); 22.94, 23.19 (CH_3-26,27-chol); 24.97 (CH_2-23-chol); 25.31 (CH_2-15-chol); 29.14 (CH-25-chol); 29.33 (CH_2-16-chol); 29.43 (CH_2-2-chol); 33.05 (CH_2-7-chol); 33.23 (CH-8-chol); 37.14 (CH-20-chol); 37.37 (CH_2-22-chol); 37.94 (C-10-chol); 38.42 (CH_2-1-chol); 40.15 (CH_2-4-chol); 40.68 (CH_2-24-chol); 41.14 (CH_2-12-chol); 41.49 (CH_2-2′); 43.49 (C-13-chol); 47.49 (CH_3**C**H_2N); 51.68 (CH-9-chol); 57.56 (CH-17-chol); 58.15 (CH-14-chol); 59.92 (C≡C**C**H_2O); 66.49 (d, $J_{C,P} = 5.6$, CH_2-5′); 68.36, 70.48, 71.49 (OCH_2CH_2O); 71.72 (CH-3′); 71.88 (OCH_2CH_2O); 77.33 (**C**≡CCH_2O); 80.87 (CH-3-chol); 87.66 (d, $J_{C,P} = 8.9$, CH-4′); 87.73 (CH-1′); 92.48 (C-5); 93.44 (C≡**C**CH_2O); 122.69 (CH-6-chol); 141.95 (C-5-chol); 146.27 (CH-6); 154.94 (C-2); 165.20 (C-4). $^{31}P\{^1H\}$ NMR (202.4 MHz, CD_3OD): -24.00 (bdd, J = 21.2, 20.1, P_β); -11.41 (d, J = 21.2, P_α); -10.65 (d, J = 20.1, P_γ).

Cholesteryl 4-pentynyl ether, compound 10

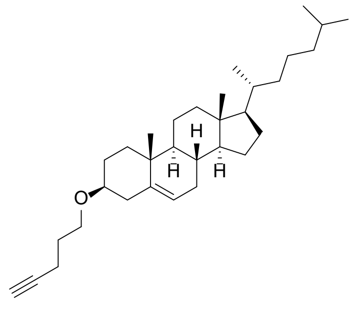

Montmorillonite K-10 clay (1 g) was activated at 120 °C under vacuo for 16 h. Then it was suspended in dry $CHCl_3$ (5 mL) and cholesterol (500 mg, 1.293 mmol) and 4-pentyn-1-ol (544 mg, 6.465 mmol) were added. Suspension was stirred for 20 h at 90 °C in microwave reactor, diluted

by 25 mL of $CHCl_3$ and filtered, concentrated under vacuo and purified using normal phase silica gel flash chromatography with cyclohexane-AcOEt mobile phase (linear gradient). Product was obtained as a white lyophilizate in 66% yield (400 mg). R_f (cyclohexane/AcOEt, 3:1) = 0.90 HR MS (ESI+) for $C_{32}H_{52}ONa$ $[M + Na]^{1+}$ calcd.: 475.3910 found: 475.3904
Spectral data were in accordance with literature.[3]

5-(5-*O*-Cholesteryl-5-oxy-1-pentynyl)-2'-deoxycytidine, compound 11, dC^{Calk}

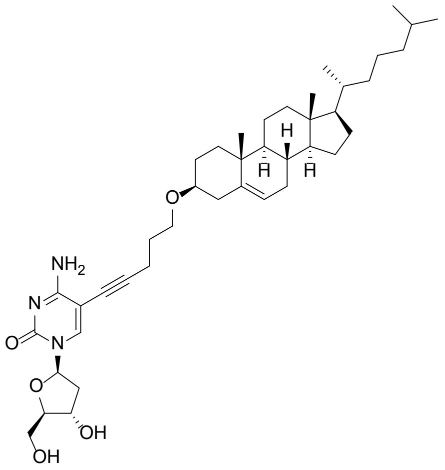

In argon purged flask, 2'-deoxy-5-iodocytidine (100 mg, 0.283 mmol), cholesteryl 4-pentynyl ether (**10**; 256 mg, 0.566 mmol), $Pd(PPh_3)_4$ (33 mg, 0.028 mmol) and CuI (11 mg, 0.057 mmol) were dissolved in dry DMF (1.5 mL) and 0.1 mL of Et_3N was added. Reaction was stirred for 2 h at 75 °C, concentrated under vacuo and residual DMF was removed by several co-distillations with toluene. Crude mixture was purified using normal phase silica gel flash chromatography with DCM-MeOH mobile phase (linear gradient). Product was obtained as a white lyophilizate in 78% yield (150 mg). R_f (DCM/MeOH, 7:1) = 0.85 HR MS (ESI-) for $C_{41}H_{63}N_3O_5Na$ $[M + Na]^{1+}$ calcd.: 700.4660 found: 700.4661; ^{1}H NMR (500.0 MHz, DMSO-d_6): 0.65 (s, 3H, CH_3-18-chol); 0.838, 0.842 (2 × d, 2 × 3H, $J_{26\&27,25}$ = 6.6, CH_3-26,27-chol); 0.88 (m, 1H, H-9-chol); 0.89 (d, 3H, $J_{21,20}$ = 6.5, CH_3-21-chol); 0.94 (s, 3H, CH_3-19-chol); 0.95 – 1.15 (m, 9H, H-1α,12α,14,15α,17,22b,23b,24-chol); 1.22 (m, 1H, H-16β–chol); 1.27 – 1.44 (m, 6H, H-2β,8,11b,20,22a,23a-chol); 1.45 – 1.57 (m, 4H, H-7α,11a,15β,25-chol); 1.69 – 1.86 (m, 5H, H-1β,2α,16α-chol, $OCH_2C\mathbf{H_2}CH_2C{\equiv}C$); 1.91 (m, 1H, H-7β-chol); 1.93 – 1.99 (m, 2H, H-2'b,H-12β-chol); 2.07 (m, 1H, H-4β-chol); 2.12 (ddd, 1H, J_{gem} =

13.2, $J_{2'a,1'}$ = 6.0, $J_{2'a,3'}$ = 3.4, H-2'a); 2.32 (ddd, 1H, J_{gem} = 13.2, $J_{4\alpha,3}$ = 4.6, $J_{4\alpha,2\alpha}$ = 2.2, H-4α-chol); 2.43 (t, 2H, J_{vic} = 7.2, $OCH_2CH_2C\mathbf{H_2}C{\equiv}C$); 3.09 (m, 1H, H-3-chol); 3.44 – 3.51 (m, 2H, $OC\mathbf{H_2}CH_2CH_2C{\equiv}C$); 3.54, 3.56 (2 × ddd, 2 × 1H, J_{gem} = 11.8, $J_{5',OH}$ = 5.1, $J_{5',4'}$ = 3.6, H-5'); 3.78 (q, 1H, $J_{4',3'}$ = $J_{4',5'}$ = 3.6, H-4'); 4.19 (m, 1H, H-3'); 5.04 (t, 1H, $J_{OH,5'}$ = 5.1, OH-5'); 5.19 (d, 1H, $J_{OH,3'}$ = 4.2, OH-3'); 5.31 (dt, 1H, $J_{6,7\beta}$ = 5.5, $J_{6,4\beta}$ = $J_{6,7\alpha}$ = 1.9, H-6-chol); 6.11 (dd, 1H, $J_{1',2'}$ = 7.3, 6.0, H-1'); 6.72, 7.68 (2 × bs, 2 × 1H, NH_2); 8.06 (s, 1H, H-6). ^{13}C NMR (125.7 MHz, DMSO-d_6): 11.70 (CH_3-18-chol); 16.16 ($C{\equiv}C\mathbf{C}H_2CH_2CH_2O$); 18.57 ($CH_3$-21-chol); 19.10 ($CH_3$-19-chol); 20.62 ($CH_2$-11-chol); 22.42, 22.70 (CH_3-26,27-chol); 23.20 (CH_2-23-chol); 23.89 (CH_2-15-chol); 27.42 (CH-25-chol); 27.80 (CH_2-16-chol); 28.13 (CH_2-2-chol); 28.72 ($C{\equiv}CCH_2\mathbf{C}H_2CH_2O$); 31.38 ($CH_2$-7-chol); 31.43 (CH-8-chol); 35.21 (CH-20-chol); 35.66 (CH_2-22-chol); 36.33 (C-10-chol); 36.70 (CH_2-1-chol); 38.79 (CH_2-4-chol); 38.95 (CH_2-24-chol); 39.26 (CH_2-12-chol); 40.73 (CH_2-2'); 41.87 (C-13-chol); 49.61 (CH-9-chol); 55.58 (CH-17-chol); 56.19 (CH-14-chol); 61.08 (CH_2-5'); 65.95 ($C{\equiv}CCH_2CH_2\mathbf{C}H_2O$); 70.20 (CH-3'); 72.08 ($\mathbf{C}{\equiv}CCH_2CH_2CH_2O$); 78.18 (CH-3-chol); 85.25 (CH-1'); 87.42 (CH-4'); 90.37 (C-5); 95.28 ($C{\equiv}\mathbf{C}CH_2CH_2CH_2O$); 121.09 (CH-6-chol); 140.56 (C-5-chol); 143.52 (CH-6); 153.49 (C-2); 164.39 (C-4).

5-(5-*O*-Cholesteryl-5-oxy-1-pentynyl)-2'-deoxycytidine 5'-*O*-triphosphate, compound 12, dC^{Calk}TP

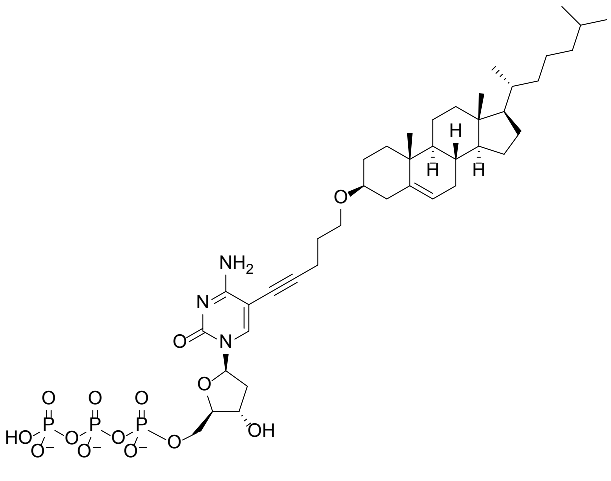

dC^{Calk} (**11**; 70 mg, 0.103 mmol) was stirred under vacuo for 16 h at 65 °C. Then it was dissolved in dry $PO(OMe)_3$ (0.7 mL) under argon atmosphere and cooled on ice-salt bath. $POCl_3$ (12 μL) was added through septum and stirred for 2 h at 0 °C. In another argon purged flask was

bis(tributylammonium) pyrophosphate (283 mg, 0.516 mmol) dissolved in dry DMF (0.5 mL) and after subsequent addition of Bu_3N (100 μL) and cooling down on ice-salt bath was mixture added to the reaction. Reaction was stirred for additional 1 h at 0 °C. 2 M TEAB (0.5 mL) was added dropwise and stirred for 15 min at 22 °C. Reaction was concentrated under vacuo. Product was purified on RP-HPLC with use of a linear gradient of 0.1 M TEAB (triethylammonium bicarbonate) in H_2O to 0.1 M TEAB in H_2O/MeCN (1:1) to MeCN as eluent in 90 minutes. Several co-distillations with water and subsequent lyophilization gave product in form of Et_3NH^+ salt as a white gummy solid in 9% yield (11 mg). HR MS (ESI-) for $C_{41}H_{65}N_3O_{14}P_3$ [M – H]$^{1-}$ calcd.: 916.3685 found: 916.3681; ^{1}H NMR (500.0 MHz, CD_3OD): 0.72 (s, 3H, CH_3-18-chol); 0.881, 0.885 (2 × d, 2 × 3H, $J_{26\&27,25}$ = 6.6, CH_3-26,27-chol); 0.95 (d, 3H, $J_{21,20}$ = 6.5, CH_3-21-chol); 0.96 (m, 1H, H-9-chol); 0.99 – 1.08 (s, 6H, H-1α,14,22b-chol, CH_3-19-chol); 1.08 – 1.22 (m, 6H, H-12α,15α,17,23b,24-chol); 1.29 (m, 1H, H-16β–chol); 1.31 (t, 18H, J_{vic} = 7.2, C**H₃**CH₂N); 1.35 – 1.58 (m, 9H, H-2β,7α,8,11,20,22a,23a,25-chol); 1.61 (m, 1H, H-15β-chol); 1.80 – 1.94 (m, 5H, H-1β,2α,16α-chol, OCH₂C**H₂**CH₂C≡C); 1.97 (m, 1H, H-7β-chol); 2.05 (dt, 1H, J_{gem} = 12.8, $J_{12\beta,11}$ = 3.5, H-12β-chol); 2.12 – 2.21 (m, 2H, H-2′b, H-4β-chol); 2.35 (ddd, 1H, J_{gem} = 13.8, $J_{2'a,1'}$ = 6.0, $J_{2'a,3'}$ = 3.4, H-2′a); 2.36 (ddd, 1H, J_{gem} = 13.1, $J_{4\alpha,3}$ = 4.7, $J_{4\alpha,2\alpha}$ = 2.3, H-4α-chol); 2.54 (t, 2H, J_{vic} = 7.3, OCH₂CH₂C**H₂**C≡C); 3.16 (m, 1H, H-3-chol); 3.20 (q, 12H, J_{vic} = 7.2, CH₃C**H₂**N); 3.60 (t, 2H, J_{vic} = 6.1, OC**H₂**CH₂CH₂C≡C); 4.08 (m, 1H, H-4′); 4.20 (ddd, 1H, J_{gem} = 11.1, $J_{H,P}$ = 5.3, $J_{5'b,4'}$ = 4.4, H-5′b); 4.28 (ddd, 1H, J_{gem} = 11.1, $J_{H,P}$ = 6.9, $J_{5'a,4'}$ = 3.8, H-5′a); 4.19 (dt, 1H, $J_{3',2'}$ = 6.5, 3.4, $J_{3',4'}$ = 3.4, H-3′); 5.35 (dt, 1H, $J_{6,7\beta}$ = 5.5, $J_{6,4\beta} = J_{6,7\alpha}$ = 2.0, H-6-chol); 6.22 (dd, 1H, $J_{1',2'}$ = 7.4, 6.0, H-1′); 8.04 (s, 1H, H-6). ^{13}C NMR (125.7 MHz, CD_3OD): 9.12 (CH₃CH₂N); 12.32 (CH₃-18-chol); 17.41 (C≡C**C**H₂CH₂CH₂O); 19.25 (CH₃-21-chol); 19.87 (CH₃-19-chol); 22.19 (CH₂-11-chol); 22.94, 23.19 (CH₃-26,27-chol); 24.96 (CH₂-23-chol); 25.32 (CH₂-15-chol); 29.16 (CH-25-chol); 29.34 (CH₂-16-chol); 29.55 (CH₂-2-chol); 30.11 (C≡CCH₂**C**H₂CH₂O); 33.06 (CH₂-7-chol); 33.25 (CH-8-chol); 37.15 (CH-20-chol); 37.38 (CH₂-22-chol); 37.97 (C-10-chol); 38.45 (CH₂-1-chol); 40.28 (CH₂-4-chol); 40.70 (CH₂-24-chol); 41.17 (CH₂-12-chol); 41.30 (CH₂-2′); 43.50 (C-13-chol); 47.47 (CH₃**C**H₂N); 51.71 (CH-9-chol); 57.58 (CH-17-chol); 58.18 (CH-14-chol); 66.60 (d, $J_{C,P}$ = 5.4, CH₂-5′); 67.87 (C≡CCH₂CH₂**C**H₂O); 71.52 (**C**≡CCH₂CH₂CH₂O); 71.96 (CH-3′); 80.54 (CH-3-chol); 87.61 (CH-1′); 87.62 (d, $J_{C,P}$ = 8.6, CH-4′); 93.79 (C-5); 97.99 (C≡**C**CH₂CH₂CH₂O); 122.69 (CH-6-chol); 141.98 (C-5-chol); 144.86 (CH-6);

155.18 (C-2); 165.52 (C-4). $^{31}P\{^{1}H\}$ NMR (202.4 MHz, CD_3OD): -23.91 (t, J = 20.9, P_β); -11.31 (d, J = 20.9, P_α); -10.59 (d, J = 20.9, P_γ).

(1,2-Dipalmitoyl-*sn*-glycero-3-phospho)ethyl-4-pentynamide, compound 13

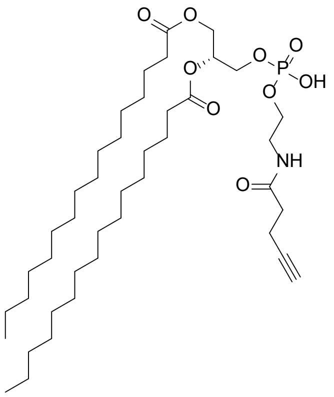

In argon purged flask, 4-pentynoic acid (16 mg, 0.173 mmol), *N*-hydroxysuccinimide (NHS; 20 mg, 0.173 mmol) and 1-ethyl-3-(3,3-dimethyaminopropyl)carbodiimide (EDCI; 27 mg, 0.173 mmol) were dissolved in dry DCM (1 mL) and stirred for 1 h at 22 °C. Reaction mixture was then added to a solution of 1,2-dipalmitoyl-*sn*-glycero-3-phosphoethanolamine (100 mg, 0.145 mmol) in dry DCM (1 mL) and *N,N*-diisopropylethylamine (DIPEA; 26 μL) was subsequently added. Reaction was stirred for 18 h at 22 °C, diluted by 15 mL of DCM and washed with H_2O (2×5 mL), dried with $MgSO_4$ and concentrated under vacuo. Product was purified using normal phase silica gel flash chromatography with $CHCl_3$-MeOH (containing 15 % v/v water) mobile phase (linear gradient). Product was obtained as a white lyophilizate in 58% yield (65 mg). R_f ($CHCl_3$/MeOH/H_2O, 65:25:4) = 0.65 HR MS (ESI-) for $C_{42}H_{78}NO_9PNa$ [M + Na]$^{1+}$ calcd.: 770.5341 found: 770.5326; ^{1}H NMR (500.2 MHz, CD_3OD, T = 60 °C): 0.87 – 0.92 (m, 6H, C**H₃**(CH₂)₁₃CH₂CO); 1.24 – 1.39 (m, 52H, CH₃(C**H₂**)₁₂CH₂CH₂CO); 1.56 – 1.66 (m, 4H, CH₃(CH₂)₁₂C**H₂**CH₂CO); 2.22 (t, 1H, ^{4}J = 2.5, **H**C≡CCH₂CH₂); 2.30 – 2.35 (m, 4H, CH₃(CH₂)₁₃C**H₂**CO); 2.41 – 2.51 (m, 4H, HC≡CC**H₂**C**H₂**); 3.44 (t, 2H, J_{vic} = 5.8, NC**H₂**CH₂O); 3.91 (dt, 2H, $J_{H,P}$ = 8.5, J_{vic} = 5.8, NCH₂C**H₂**O); 3.97 – 4.01 (m, 2H, H-1); 4.19 (dd, 1H, J_{gem} = 12.0, $J_{3b,2}$ = 6.8, H-3b); 4.42 (dd, 1H, J_{gem} = 12.0, $J_{3a,2}$ = 3.2, H-3a); 5.22 (m, 1H, H-2). ^{13}C NMR (125.8 MHz, CD_3OD, T = 60 °C): 14.28 (**C**H₃(CH₂)₁₃CH₂CO); 15.67 (HC≡C**C**H₂CH₂CO); 22.85 (CH₃**C**H₂(CH₂)₁₂CH₂CO); 25.99, 26.02 (CH₃(CH₂)₁₂**C**H₂CH₂CO); 30.16, 30.17, 30.33, 30.36, 30.53, 30.55, 30.65, 30.68, 32.97 (CH₃CH₂(**C**H₂)₁₁CH₂CH₂CO); 35.01, 30.20 (CH₃(CH₂)₁₃**C**H₂CO);

36.15 ($HC\equiv CCH_2\mathbf{C}H_2CO$); 41.40 (d, $J_{C,P}$ = 5.9, N**C**H_2CH_2O); 63.81 (CH_2-3); 64.92 (d, $J_{C,P}$ = 4.9, CH_2-1); 65.21 (d, $J_{C,P}$ = 5.5, $NCH_2\mathbf{C}H_2O$); 70.23 (H**C**$\equiv CCH_2CH_2CO$); 72.05 (d, $J_{C,P}$ = 8.3, CH-2); 83.47 ($HC\equiv\mathbf{C}CH_2CH_2CO$); 174.30 (CONH); 174.71, 175.03 ($CH_3(CH_2)_{13}CH_2\mathbf{C}O$). $^{31}P\{^1H\}$ NMR (202.5 MHz, CD_3OD, T = 60 °C): -1.07.

7-deaza-7-[*N*-(1,2-dipalmitoyl-*sn*-glycero-3-phosphoethylo)-5-amino-5-oxo-1-pentynyl]-2'-deoxyadenisine 5'-*O*-triphosphate, compound 14, dA^{DPPE}TP

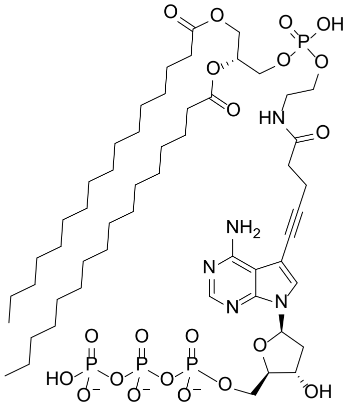

In argon purged flask, 7-deaza-7-iodo-2'-deoxyadenosine 5'-*O*-triphosphate in Et_3NH^+ salt form (20 mg, 0.0217 mmol), (1,2-Dipalmitoyl-*sn*-glycero-3-phospho)ethyl-4-pentynamide (20 mg, 0.0260 mmol), $Pd(PPh_3)_2Cl_2$ (3 mg, 0.0044 mmol) and CuI (0.8 mg, 0.0044 mmol) were dissolved in dry DMF (0.5 mL) and 30 μL of Et_3N was added. Reaction was stirred for 1 h at 75 °C under argon atmosphere. Ethylenediaminetetraacetic acid (EDTA; 14 mg/mL H_2O, 0.5 ml) was added and stirred for additional 10 min at 22 °C. Reaction was concentrated under vacuo. Product was purified on RP-HPLC with use of a linear gradient of 0.1 M TEAB (triethylammonium bicarbonate) in H_2O to 0.1 M TEAB in H_2O/MeOH (1:1) to MeOH as eluent in 90 minutes. Several co-distillations with water and subsequent lyophilization gave product in form of Et_3NH^+ salt as a white gummy solid in 29% yield (10 mg). HR MS (ESI-) for $C_{53}H_{92}N_5O_{21}P_4$ $[M - H]^{1-}$ calcd.: 1258.5241 found: 1258.5231; ^1H NMR (600.1 MHz, CD_3OD): 0.88 – 0.91 (m, 6H, $C\mathbf{H_3}(CH_2)_{13}CH_2CO$); 1.25 – 1.34 (m, 79H, $CH_3(C\mathbf{H_2})_{12}CH_2CH_2CO$, $C\mathbf{H_3}CH_2N$); 1.55 – 1.62 (m, 4H, $CH_3(CH_2)_{12}C\mathbf{H_2}CH_2CO$); 2.28 – 2.34 (m, 4H, $CH_3(CH_2)_{13}C\mathbf{H_2}CO$); 2.35 (ddd, 1H, J_{gem} = 13.6, $J_{2'b,1'}$ = 6.1, $J_{2'b,3'}$ = 3.1, H-2'b); 2.53 – 2.56 (m, 2H, $C\equiv CCH_2C\mathbf{H_2}CO$); 2.57 (ddd, 1H, J_{gem} = 13.6, $J_{2'a,1'}$ = 7.8, $J_{2'a,3'}$ = 6.0, H-2'a); 2.70 (dt, 1H, J_{gem} = 17.0, J_{vic} = 6.6, $C\equiv CCH_a\mathbf{H_b}CH_2CO$); 2.74 (dt, 1H, J_{gem} = 17.0, J_{vic} = 7.3, $C\equiv CCH_a\mathbf{H_b}CH_2CO$); 3.19 (q,

18H, J_{vic} = 7.3, $CH_3C\mathbf{H_2}N$); 3.46 (t, 2H, J_{vic} = 5.6, $NC\mathbf{H_2}CH_2O$); 3.91 (dt, 2H, $J_{H,P}$ = 6.9, J_{vic} = 5.6, $NCH_2C\mathbf{H_2}O$); 3.98 (dd, 2H, $J_{H,P}$ = 6.0, $J_{1'',2''}$ = 5.3, H-1″); 4.13 (tdd, 1H, $J_{4',5'}$ = 3.9, $J_{4',3'}$ = 2.7, $J_{H,P}$ = 1.4, H-4′); 4.17 (dd, 1H, J_{gem} = 12.0, $J_{3''b,2''}$ = 6.8, H-3″b); 4.21 (ddd, 1H, J_{gem} = 11.2, $J_{H,P}$ = 5.4, $J_{5'b,4'}$ = 3.9, H-5′b); 4.26 (dt, 1H, J_{gem} = 11.2, $J_{H,P}$ = $J_{5'a,4'}$ = 3.9, H-5′a); 4.42 (dd, 1H, J_{gem} = 12.0, $J_{3''a,2''}$ = 3.2, H-3″a); 4.67 (ddd, 1H, $J_{3',2'}$ = 6.0, 3.1, $J_{3',4'}$ = 2.8, H-3′); 5.22 (m, 1H, H-2″); 6.62 (dd, 1H, $J_{1',2'}$ = 7.8, 6.1, H-1′); 7.71 (s, 1H, H-6); 8.23 (s, 1H, H-2). ^{13}C NMR (150.9 MHz, CD_3OD): 9.15 ($\mathbf{C}H_3CH_2N$); 14.46 ($\mathbf{C}H_3(CH_2)_{13}CH_2CO$); 16.84 ($C{\equiv}C\mathbf{C}H_2CH_2CO$); 23.74 ($CH_3\mathbf{C}H_2(CH_2)_{12}CH_2CO$); 26.03, 26.05 ($CH_3(CH_2)_{12}\mathbf{C}H_2CH_2CO$); 30.21, 30.23, 30.49, 30.51, 30.67, 30.69, 30.78, 30.79, 30.80, 30.83, 33.08 ($CH_3CH_2(\mathbf{C}H_2)_{11}CH_2CH_2CO$); 34.95, 35.13 ($CH_3(CH_2)_{13}\mathbf{C}H_2CO$); 35.28 ($C{\equiv}CCH_2\mathbf{C}H_2CO$); 41.38 (d, $J_{C,P}$ = 7.5, $N\mathbf{C}H_2CH_2O$); 41.50 (CH_2-2′); 47.53 ($CH_3\mathbf{C}H_2N$); 63.67 (CH_2-3″); 64.73 (d, $J_{C,P}$ = 5.2, CH_2-1″); 65.22 (d, $J_{C,P}$ = 5.5, $NCH_2\mathbf{C}H_2O$); 66.99 (d, $J_{C,P}$ = 5.7, CH_2-5′); 71.89 (d, $J_{C,P}$ = 8.3, CH-2″); 72.72 (CH-3′); 73.30 ($\mathbf{C}{\equiv}CCH_2CH_2CO$); 85.00 (CH-1′); 87.55 (d, $J_{C,P}$ = 8.7, CH-4′); 93.70 ($HC{\equiv}\mathbf{C}CH_2CH_2CO$); 99.43 (C-5); 103.40 (C-4a); 127.77 (CH-6); 148.55 (CH-2); 148.87 (C-7a); 155.20 (C-4); 174.16 (CONH); 174.60, 174.91 ($CH_3(CH_2)_{13}CH_2\mathbf{C}O$). $^{31}P\{^1H\}$ NMR (202.4 MHz, CD_3OD): -22.06 (bdd, J = 20.9, 19.8, P_β); -9.66 (d, J = 20.9, P_α); -8.80 (d, J = 19.8, P_γ); -1.07 (s, P-phosphate).

(1,2-Di-*O*-hexadecyl-*sn*-glycero-3-phospho)ethyl-4-pentynamide, compound 15

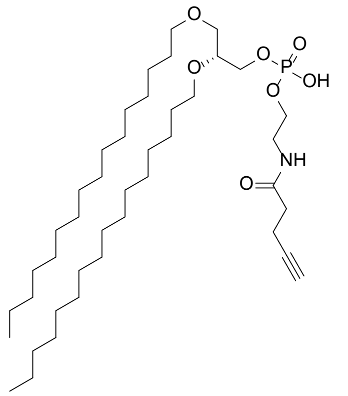

In argon purged flask, 4-pentynoic acid (18 mg, 0.181 mmol), NHS (21 mg, 0.181 mmol) and 1-EDCI (35 mg, 0.181 mmol) were dissolved in dry DCM (1 mL) and stirred for 1 h at 22 °C. Reaction mixture was then added to a solution of 1,2-di-*O*-hexadecyl-*sn*-glycero-3-phosphoethanolamine (100 mg, 0.151 mmol) in dry DCM (1 mL) and DIPEA (26 μL) was subsequently added. Reaction was stirred for 18 h at 22 °C, diluted by 15 mL of DCM and washed with H_2O (2×5 mL), dried with

$MgSO_4$ and concentrated under vacuo. Product was purified using normal phase silica gel flash chromatography with $CHCl_3$-MeOH (containing 15 % v/v water) mobile phase (linear gradient). Product was obtained as a white lyophilizate in 67% yield (75 mg). R_f ($CHCl_3$/MeOH/H_2O, 65:25:4) = 0.72 HR MS (ESI-) for $C_{42}H_{82}NO_7PNa$ $[M + Na]^{1+}$ calcd.: 766.5721 found: 766.5726; ^1H NMR (500.0 MHz, $CDCl_3$): 0.85 – 0.90 (m, 6H, C**H$_3$**$(CH_2)_{14}CH_2O$); 1.20 – 1.33 (m, 52H, CH_3(C**H$_2$**)$_{13}CH_2CH_2O$); 1.49 – 1.58 (m, 4H, $CH_3(CH_2)_{13}$C**H$_2$**CH_2O); 1.99 (t, 1H, ^4J = 2.5, **H**C≡CCH_2CH_2); 2.44 – 2.49 (m, 2H, HC≡CCH_2C**H$_2$**); 2.49 – 2.54 (m, 2H, HC≡CC**H$_2$**CH_2); 3.37 – 3.65 (m, 9H, H-2,3, NC**H$_2$**CH_2O, $CH_3(CH_2)_{14}$C**H$_2$**O); 3.86 – 4.05 (m, 4H, H-1, NCH_2C**H$_2$**O); 7.33 (bs, 1H, NH). ^{13}C NMR (125.7 MHz, $CDCl_3$): 14.27 (**C**$H_3(CH_2)_{14}CH_2O$); 14.89 (HC≡C**C**H_2CH_2CO); 22.85, 26.24, 26.29, 29.53, 29.76; 29.83, 29.89, 29.90, 29.91, 30.20, 32.08 (CH_3(**C**H_2)$_{14}CH_2O$); 35.08 (HC≡CCH_2**C**H_2CO); 40.36 (d, $J_{C,P}$ = 5.5, N**C**H_2CH_2O); 65.19 (d, $J_{C,P}$ = 4.9, NCH_2**C**H_2O); 65.79 (d, $J_{C,P}$ = 4.6, CH_2-1); 69.23 (H**C**≡CCH_2CH_2CO); 70.31 (CH_2-3); 70.83, 71.98 ($CH_3(CH_2)_{14}$**C**H_2O); 77.86 (d, $J_{C,P}$ = 8.3, CH-2); 83.51 (HC≡**C**CH_2CH_2CO); 172.22 (CONH). $^{31}P\{^1H\}$ NMR (202.4 MHz, $CDCl_3$): -1.60.

5-{4-[*N*-(1,2-di-*O*-hexadecyl-*sn*-glycero-3-phosphoethyl)-3-amino-3-oxopropyl]-1,2,3-triazole-1-*N*-yl}methyl-2'-deoxyuridine 5'-*O*-triphosphate, compound 16, dT^{DHPE}TP

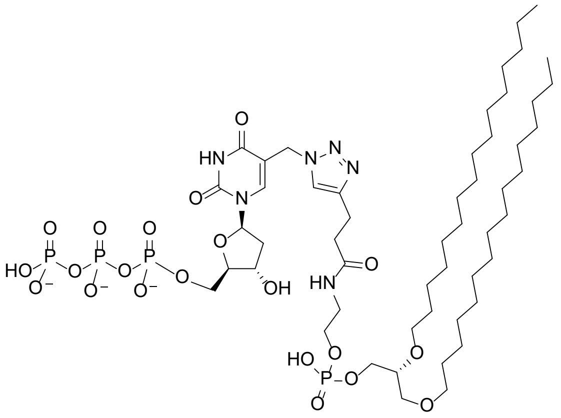

In argon purged flask were (1,2-Di-*O*-hexadecyl-*sn*-glycero-3-phospho)ethyl-4-pentynamide (12 mg, 0.016 mmol) and 5-azidomethyl-2'-deoxyuridine in Et_3NH^+ salt form (9 mg, 0.011 mmol) were dissolved in degassed DMSO (1 mL) and $CuSO_4 \cdot 5\ H_2O$ (0.3 mg, 0.0011 mmol in 10 μL H_2O) and sodium ascorbate (1 mg, 0.005 mmol in 10 μL H_2O) were added and reaction was stirred for 4 h at 22 °C. EDTA (14 mg/mL H_2O, 0.5 ml) was added and stirred for additional 10 min at 22 °C.

Solvents were lyophilized and product was purified on RP-HPLC with use of a linear gradient of 0.1 M TEAB (triethylammonium bicarbonate) in H_2O to 0.1 M TEAB in H_2O/MeOH (1:1) to MeOH as eluent in 90 minutes. Several co-distillations with water and subsequent lyophilization gave product in form of Et_3NH^+ salt as a white gummy solid in 59% yield (10 mg). HR MS (ESI-) for $C_{52}H_{97}N_6O_{21}P_4$ $[M - H]^{1-}$ calcd.: 1265.5663 found: 1265.5657; ^{1}H NMR (500.0 MHz, CD_3OD): 0.88 – 0.92 (m, 6H, C**H₃**$(CH_2)_{14}CH_2O$); 1.25 – 1.39 (m, 88H, CH_3(C**H₂**)$_{13}$$CH_2CH_2O$, C**H₃**$CH_2N$); 1.50 – 1.58 (m, 4H, $CH_3(CH_2)_{13}$C**H₂**CH_2O); 2.30 (ddd, 1H, J_{gem} = 13.6, $J_{2'b,1'}$ = 6.3, $J_{2'b,3'}$ = 4.4, H-2'b); 2.37 (dt, 1H, J_{gem} = 13.6, $J_{2'a,1'}$ = $J_{2'a,3'}$ = 6.3, H-2'a); 2.54 – 2.58 (m, 2H, triazoleCH_2C**H₂**CO); 2.94 – 2.98 (m, 2H, triazoleC**H₂**CH_2CO); 3.18 (bq, 24H, J_{vic} = 7.3, CH_3C**H₂**N); 3.38 – 3.42 (m, 2H, NC**H₂**CH_2O); 3.42 – 3.50, 3.55 – 3.60 (2 × m, 7H, H-2″,3″, $CH_3(CH_2)_{14}$C**H₂**O); 3.84 – 3.92 (m, 4H, H-1″, NCH_2C**H₂**O); 4.04 (m, 1H, H-4'); 4.28 (ddd, 1H, J_{gem} = 11.5, $J_{H,P}$ = 4.8, $J_{4',5'}$ = 3.2, H-5'b); 4.36 (bm, 1H, H-5'a); 4.66 (m, 1H, H-3'); 5.37, 5.40 (2 × d, 2 × 1H, J_{gem} = 14.5, CH_2N); 6.27 (t, 1H, $J_{1',2'}$ = 6.3, H-1'); 7.87 (s, 1H, H-5-triazole); 8.43 (s, 1H, H-6). ^{13}C NMR (125.7 MHz, CD_3OD: 9.17 (**C**H_3CH_2N); 14.46 (**C**$H_3(CH_2)_{14}CH_2O$); 22.66 (triazole**C**H_2CH_2CO); 23.75, 27.26, 27.33, 30.50, 30.66; 30.78, 30.80, 30.81, 30.83, 30.84, 30.85, 31.20, 33.09 (CH_3(**C**H_2)$_{14}$$CH_2O$); 36.41 (triazole$CH_2$**C**$H_2$CO); 41.03 ($CH_2$-2'); 41.40 (d, $J_{C,P}$ = 7.7, N**C**H_2CH_2O); 47.46 (CH_3**C**H_2N); 48.09 (CH_2N); 64.94 (d, $J_{C,P}$ = 5.6, NCH_2**C**H_2O); 65.94 (d, $J_{C,P}$ = 5.7, CH_2-1″); 66.36 (bd, $J_{C,P}$ = 5.7, CH_2-5'); 71.53 (CH-3'); 71.53 (CH_2-3″); 71.99, 72.57 ($CH_3(CH_2)_{14}$**C**H_2O); 79.45 (d, $J_{C,P}$ = 8.3, CH-2″); 86.71 (CH-1'); 87.77 (d, JC,P = 9.2, CH-4'); 109.65 (C-5); 123.81 (CH-5-triazole); 143.00 (CH-6); 147.45 (C-4-triazole); 152.14 (C-2); 164.71 (C-4); 174.65 (CONH). ^{31}P{^{1}H} NMR (202.4 MHz, CD_3OD): -23.56 (bt, J = 21.3, P_β); -11.35 (d, J = 21.3, P_α); -10.27 (d, J = 21.3, P_γ); -1.60 (s, phosphate).

2 Experimental section – biochemistry

General remarks

All gels were analyzed by fluorescence imaging using Typhoon FLA 9500 (GE Healthcare). The MALDI-TOF spectra were measured on a MALDI-TOF/TOF mass spectrometer with a 1 kHz smartbeam II laser. UV-Vis spectra were measured at room temperature in a NanoDrop1000 (ThermoScientific). Synthetic oligonucleotides (primers labelled at 5′-end with 6-carboxyfluorescein (6-FAM), Cy5, templates and biotinylated templates (for sequences see Table S1) were purchased from Generi Biotech, or Eurofins (Czech Republic), dual-biotinylated templates were purchased from biomers.net GmbH (Germany). Natural nucleoside triphosphates (dATP, dGTP, dTTP, dCTP) were purchased from ThermoScientific. KOD XL DNA polymerase and corresponding polymerase reaction buffer from Merck (Sigma Aldrich). Streptavidin magnetic particles (Roche) were obtained from Sigma Aldrich. Micro Bio-Spin® P-6 and P-30 comlumns for dsDNA purification were purchased from BioRad. Milli-Q water was used for all experiments. PAGE stop solution used after PEX reactions contains: 95% [v/v] formamide, 0.5 M EDTA, 0.025% [w/v] bromophenol blue and 0.025% [w/v] xylene cyanol, 0.025% [w/v] SDS and Milli-Q water. Samples after PEX reactions were analysed by a 12.5% denaturating PAGE (acrylamide/bisacrylamide 19:1, 25% urea) under denaturing conditions (1 h, 50 °C, 1× TBE). Native loading dye used after PEX reactions contains: 0.07% [w/v] bromophenol blue, 0.07% [w/v] xylene cyanol and 13.3% [w/v] saccharose. Samples after PEX reactions were analysed by a 12.5% native PAGE (acrylamide/bisacrylamide 19:1), (2 h, 8 °C, 1× TBE). Other chemicals were of analytical grade. Samples after PCR reactions were separated with a 4% agarose gel (Agarose, research grade, Serva) in 0.5× TBE buffer. Cell counting was done using LUNA Automated Cell Counter. Confocal microscopy was done using confocal microscope ZEISS LSM 980 fitted with an incubator and objective 40x/1.2 W C-Apochromat (Carl Zeiss Jena GmbH, Germany) and glass bottom wells, or 96 Well Glass Bottom Plate (Cellvis, P96-1.5H-N). Flow cytometry was done using flow cytometry analyzer CytoFlex LX and CytExpert Acquisition and Analysis Software v.2.5 (Beckman Coulter Life Sciences, USA).

Table S1. List of sequences of primers and templates.

Name	Size (nt)	Sequence (5’ → 3’)
[a]prim^{rnd}	15	5’-CATGGGCGGCCATGGG-3’
[a]prim ^{LT25TH}	25	5’-CAAGGACAAAATACCTGTATTCCTT-3’
[a]prim^{L20-}	20	5’-GACATCATGAGAGACATCGC-3’
[b]temp^{TermT}	16	5’-ACCCATGCCGCCCATG-3’
[b]temp^{TermC}	16	5’-GCCCATGCCGCCCATG-3’
[b]temp^{1T}	19	5’-CCCACCCATGCCGCCCATG-3’
[b]temp^{1A}	19	5’-CCCTCCCATGCCGCCCATG-3’
[b]temp^{1C}	19	5’-CCCGCCCATGCCGCCCATG-3’
[b]temp^{rnd8}	23	5’-CATGCAGTCCCATGCCGCCCATG-3‘
[b]temp^{rnd16}	31	5’-ATGCTCGAGTACGATCCCCATGCCGCCCATG-3‘
temp^{FVLA}	98	5’-GACATCATGAGAGACATCGCCTCTGGGCTAATAGGACTACTTCTAA TCTGTAAGAGCAGATCCCTGGACAGGCAAGGAATACAGGTATTTTGT CCTTG-3’
[b]temp^{TCT}	18	5'-AGACCCATGCCGCCCATG-3‘
[b]temp$^{T(1)C(1)T}$	20	5'-ACGCACCCATGCCGCCCATG-3‘
[b]temp$^{T(2)C(2)T}$	22	5'-ACTGTCACCCATGCCGCCCATG-3‘
[b]temp$^{T(3)C(3)T}$	24	5'-ATCTGTCTACCCATGCCGCCCATG-3‘
[b]temp$^{T(5)C(5)T}$	28	5'-ATTCTCGTTCTTACCCATGCCGCCCATG-3‘
[b]temp$^{C(5)C}$	22	5'-GCTATCGCCCATGCCGCCCATG-3‘

[a]5’-(6-FAM)-labelled for PAGE and subsequent MALDI analysis, Cy5-labelled for *in vitro* experiments; [b]5’-dual biotinylated for single stranded ON preparation for MALDI analysis and *in vitro* assay; in the template ONs the segments forming duplex with the primer are underlined.

2.1 Primer extension experiments

2.1.1 Single dT^{Pam}TP incorporation

Analytical PEX: The reaction mixture (10 µL) contained temp^{1T} (3 µM, 0.75 µL) prim^{rnd} (3 µM, 0.5 µL), dGTP (0.5 mM, 0.5 µL), **dT^{Pam}TP** (0.5 mM, 1 µL), KOD XL DNA polymerase (0.3 U) and reaction buffer (10×, 1 µL) as supplied by the manufacturer. The reaction mixture was incubated for 30 minutes at 60 °C. The PEX reaction was stopped by addition of PAGE stop solution (10 µL) and heated for 2 minutes at 95 °C. Samples were analyzed by denaturing PAGE and visualized using fluorescence imaging (**Figure S1A**).

Sample for MALDI and *in vitro* experiments: The reaction mixture (50 µL) contained temp^{1T} (100 µM, 1 µL), prim^{rnd} (100 µM, 1 µL), dGTP and **dT^{Pam}TP** (2 mM, 1 µL) and KOD XL DNA polymerase (1.25 U) in the enzyme reaction buffer (10×, 5 µL) as supplied by the manufacturer. The reaction mixture was incubated for 40 minutes at 60 °C. The reaction was stopped by cooling to 4 °C.

2.1.2 Single dT^{Pam}TP incorporation in the terminal position

Analytical PEX: The reaction mixture (10 µL) contained temp^{TermT} (3 µM, 0.75 µL) prim^{rnd} (3 µM, 0.5 µL), **dT^{Pam}TP** (0.5 mM, 1 µL), KOD XL DNA polymerase (0.3 U) and reaction buffer (10×, 1 µL) as supplied by the manufacturer. The reaction mixture was incubated for 30 minutes at 60 °C. The PEX reaction was stopped by addition of PAGE stop solution (10 µL) and heated for 2 minutes at 95 °C. Samples were analyzed by denaturing PAGE and visualized using fluorescence imaging (**Figure S1B**).

Sample for MALDI and *in vitro* experiments: The reaction mixture (50 µL) contained temp^{TermT} (100 µM, 3.2 µL), prim^{rnd} (100 µM, 3.2 µL), **dT^{Pam}TP** (4 mM, 1 µL), and KOD XL DNA polymerase (1.25 U) in the enzyme reaction buffer (10×, 5 µL) as supplied by the manufacturer. The reaction mixture was incubated for 60 minutes at 60 °C. The reaction was stopped by cooling to 4 °C.

2.1.3 Multiple dT^{Pam}TP incorporation

Analytical PEX: The reaction mixture (10 µL) contained template temp^{rnd16} (3 µM, 0.75 µL), prim^{rnd} (3 µM, 0.5 µL), natural dNTPs (each 0.5 mM, 1 µL), **dT^{Pam}TP** (0.5 mM, 1 µL), KOD XL DNA

polymerase (0.5 U) and reaction buffer (10×, 1 µL) as supplied by the manufacturer. The reaction mixture was incubated for 30 minutes at 60 °C. The PEX reaction was stopped by cooling to 4 °C. Sample was mixed with a native loading dye and analysed by native PAGE and visualised using fluorescence imaging (**Figure S1C**).

Sample for MALDI and *in vitro* experiments: The reaction mixture (50 µL) contained temp^{rnd16} (100 µM, 1 µL), prim^{rnd} (100 µM, 1 µL), corresponding natural dNTPs (4 mM, 2 µL) **dT^{Pam}TP** (4 mM, 1 µL), and KOD XL DNA polymerase (3.75 U) in the enzyme reaction buffer (10×, 5 µL) as supplied by the manufacturer. The reaction mixture was incubated for 2 h at 60 C. The reaction was stopped by cooling to 4 °C.

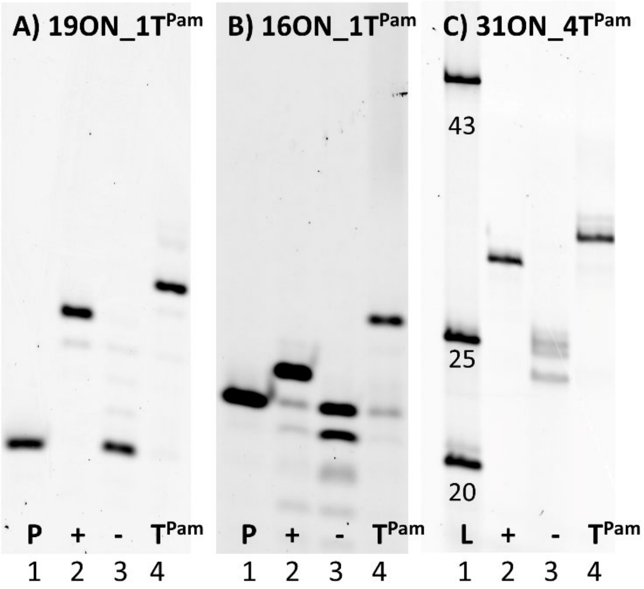

Fig. S1. PAGE analysis of PEX using **dT^{Pam}TP**, 5'-(6-FAM)-labelled prim^{rnd} and A) temp^{1T}; B) temp^{TermT}; C) temp^{rnd16}; [lanes (1) primer (denaturing PAGE (A) and (B)) or ds DNA ladder (native PAGE (C)); (2) positive control: natural dNTPs; (3) negative control: without dTTP; (4) reaction containing **dT^{Pam}TP**].

2.1.4 Single dC^{Cdeg}TP incorporation

Analytical PEX: The reaction mixture (10 µL) contained temp^{1C} (3 µM, 0.75 µL) prim^{rnd} (3 µM, 0.5 µL), dGTP (0.5 mM, 0.5 µL), **dC^{Cdeg}TP** (0.1 mM, 1 µL), KOD XL DNA polymerase (0.3 U) and reaction buffer (10×, 1 µL) as supplied by the manufacturer. The reaction mixture was incubated for 30 minutes at 60 °C. The PEX reaction was stopped by addition of PAGE stop solution (10 µL)

and heated for 2 minutes at 95 °C. Samples were analyzed by denaturing PAGE and visualized using fluorescence imaging (**Figure S2A**).

Sample for MALDI and *in vitro* experiments: The reaction mixture (50 µL) contained temp^{1C} (100 µM, 1 µL), prim^{rnd} (100 µM, 1 µL), dGTP and **dC^{Cdeg}TP** (4 mM, 1 µL), and KOD XL DNA polymerase (3.75 U) in the enzyme reaction buffer (10×, 5 µL) as supplied by the manufacturer. The reaction mixture was incubated for 60 minutes at 60 °C. The reaction was stopped by cooling to 4 °C.

2.1.5 Single dC^{Cdeg}TP incorporation in the terminal position

Analytical PEX: The reaction mixture (10 µL) contained temp^{TermC} (3 µM, 0.75 µL) prim^{rnd} (3 µM, 0.5 µL), **dC^{Cdeg}TP** (0.5 mM, 1 µL), Vent (exo-) polymerase (0.3 U) and reaction buffer (10×, 1 µL) as supplied by the manufacturer. The reaction mixture was incubated for 40 minutes at 60 °C (only 10 min at 60 °C for positive control). The PEX reaction was stopped by addition of PAGE stop solution (10 µL) and heated for 2 minutes at 95 °C. Samples were analyzed by denaturing PAGE and visualized using fluorescence imaging (**Figure S2B**).

Sample for MALDI and *in vitro* experiments: The reaction mixture (50 µL) contained temp^{TermC} (100 µM, 1 µL), prim^{rnd} (100 µM, 1 µL), **dC^{Cdeg}TP** (4 mM, 1 µL), and Vent (exo-) DNA polymerase (10 U), enzyme reaction buffer (10×, 5 µL) as supplied by the manufacturer and 10% (v/v) PEG200. The reaction mixture was incubated for 2 h at 60 °C. The reaction was stopped by cooling to 4 °C.

2.1.6 Multiple dC^{Cdeg}TP incorporation

Sample for analytical PEX, MALDI and *in vitro* experiments: The reaction mixture (50 µL) contained temp^{rnd8} (100 µM, 1 µL), prim^{rnd} (100 µM, 1 µL), corresponding natural dNTPs (4 mM, 1 µL) and **dC^{Cdeg}TP** (2 mM, 1 µL), and KOD XL DNA polymerase (3.75 U) in the enzyme reaction buffer (10×, 5 µL) as supplied by the manufacturer. The reaction mixture was incubated for 2 h at 60 °C. The reaction was stopped by cooling to 4 °C. 10× Diluted PEX samples were analyzed by native PAGE and visualized using fluorescence imaging (**Figure S2C**).

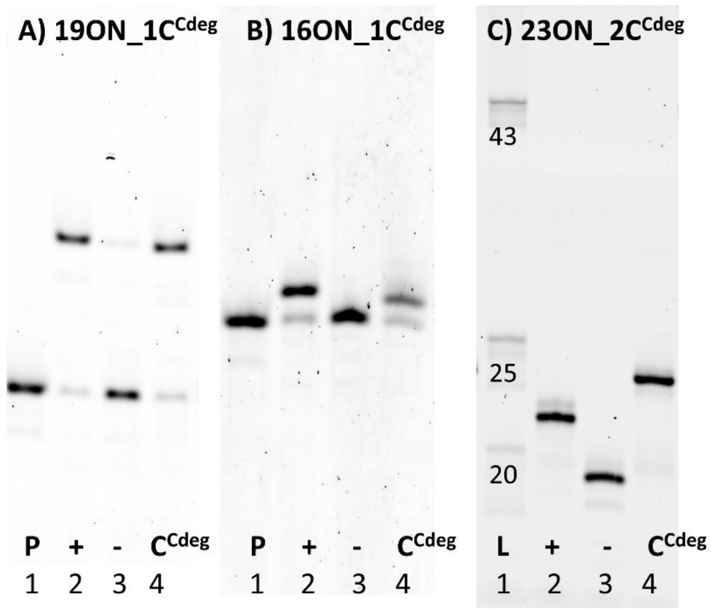

Fig. S2. PAGE analysis of PEX using **dC^Cdeg^TP**, 5'-(6-FAM)-labelled prim*^rnd^* and (A) temp*^1C^*; (B) temp*^TermC^*; (C) temp*^rnd8^*; [lanes (1) primer (denaturing PAGE (A) and (B)) or ds DNA ladder (native PAGE (C)); (2) positive control: natural dNTPs; (3) negative control: without dTTP; (4) reaction containing **dC^Cdeg^TP**].

2.1.7 Single dC^Calk^TP incorporation

Analytical PEX: The reaction mixture (10 µL) contained temp*^1C^* (3 µM, 0.75 µL) prim*^rnd^* (3 µM, 0.5 µL), dGTP (0.5 mM, 0.5 µL), **dC^Calk^TP** (0.1 mM, 1 µL), KOD XL DNA polymerase (0.3 U) and reaction buffer (10×, 1 µL) as supplied by the manufacturer. The reaction mixture was incubated for 30 minutes at 60 °C. The PEX reaction was stopped by addition of PAGE stop solution (10 µL) and heated for 2 minutes at 95 °C. Samples were analyzed by denaturing PAGE and visualized using fluorescence imaging (**Figure S3A**).

Sample for MALDI and *in vitro* experiments: The reaction mixture (50 µL) contained temp*^1C^* (100 µM, 1 µL), prim*^rnd^* (100 µM, 1 µL), dGTP and **dC^Calk^TP** (4 mM, 1 µL), and KOD XL DNA polymerase (3.75 U) in the enzyme reaction buffer (10×, 5 µL) as supplied by the manufacturer. The reaction mixture was incubated for 60 minutes at 60 °C. The reaction was stopped by cooling to 4 °C.

2.1.8 Single dC^{Calk}TP incorporation in the terminal position

Analytical PEX: The reaction mixture (10 µL) contained temp^{TermC} (3 µM, 0.75 µL) prim^{rnd} (3 µM, 0.5 µL), **dC^{Calk}TP** (0.5 mM, 1 µL), Vent (exo-) polymerase (0.3 U) and reaction buffer (10×, 1 µL) as supplied by the manufacturer. The reaction mixture was incubated for 40 minutes at 60 °C (only 10 min at 60 °C for positive control). The PEX reaction was stopped by addition of PAGE stop solution (10 µL) and heated for 2 minutes at 95 °C. Samples were analyzed by denaturing PAGE and visualized using fluorescence imaging (**Figure S3B**).

Sample for MALDI and *in vitro* experiments: The reaction mixture (50 µL) contained temp^{TermC} (100 µM, 1 µL), prim^{rnd} (100 µM, 1 µL), **dC^{Calk}TP** (4 mM, 1 µL), and Vent (exo-) DNA polymerase (10 U), enzyme reaction buffer (10×, 5 µL) as supplied by the manufacturer and 10% (v/v) PEG200. The reaction mixture was incubated for 2 h at 60 °C. The reaction was stopped by cooling to 4 °C.

2.1.9 Multiple dC^{Calk}TP incorporation

Sample for analytical PEX, MALDI and *in vitro* experiments: The reaction mixture (50 µL) contained temp^{rnd8} (100 µM, 1 µL), prim^{rnd} (100 µM, 1 µL), corresponding natural dNTPs (4 mM, 2 µL) and **dC^{Calk}TP** (2 mM, 1 µL), and KOD XL DNA polymerase (6.25 U) in the enzyme reaction buffer (10×, 5 µL) as supplied by the manufacturer. The reaction mixture was incubated for 2 h at 60 °C. The reaction was stopped by cooling to 4 °C. 10× Diluted PEX samples were analyzed by native PAGE and visualized using fluorescence imaging (**Figure S3C**).

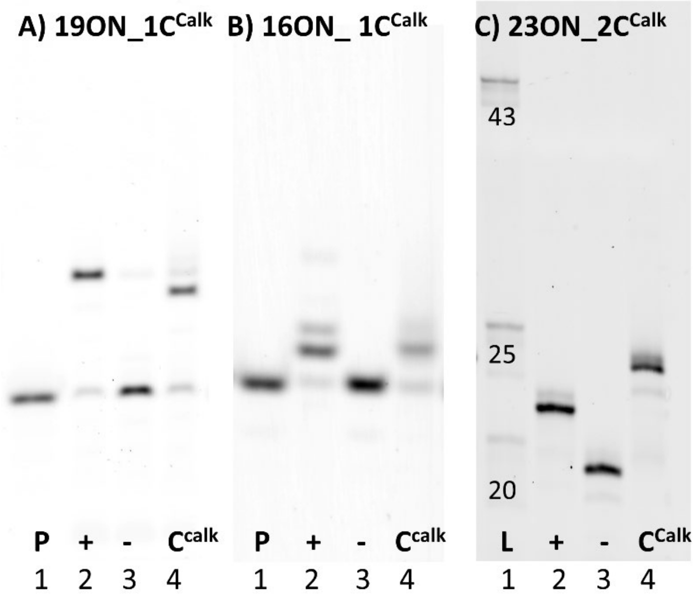

Fig. S3. PAGE analysis of PEX using **dC^Calk^TP**, 5'-(6-FAM)-labelled prim^{rnd} and (A) temp^{1C}; (B) temp^{TermC}; (C) temp^{rnd8}; [lanes (1) primer (denaturing PAGE (A) and (B)) or ds DNA ladder (native PAGE (C)); (2) positive control: natural dNTPs; (3) negative control: without dTTP; (4) reaction containing **dC^{Calk}TP**].

2.1.10 Single dC^{Cest}TP incorporation

Analytical PEX: The reaction mixture (10 µL) contained temp^{1C} (3 µM, 0.75 µL) prim^{rnd} (3 µM, 0.5 µL), dGTP (0.5 mM, 0.5 µL), **dC^{Cest}TP** (0.5 mM, 1 µL), KOD XL DNA polymerase (0.2 U) and reaction buffer (10×, 1 µL) as supplied by the manufacturer. The reaction mixture was incubated for 30 minutes at 60 °C. The PEX reaction was stopped by addition of PAGE stop solution (10 µL) and heated for 2 minutes at 95 °C. Samples were analyzed by denaturing PAGE and visualized using fluorescence imaging (**Figure S4**).

Sample for MALDI analysis: The reaction mixture (50 µL) contained temp^{1C} (3 µM, 2.5 µL) prim^{rnd} (3 µM, 2.5 µL), dGTP (0.5 mM, 2.5 µL), dC^{Cest}TP (0.5 mM, 5 µL), KOD XL DNA polymerase (1 U) and reaction buffer (10×, 5 µL) as supplied by the manufacturer. The reaction mixture was incubated for 30 minutes at 60 °C. The reaction was stopped by cooling to 4 °C.

Sample for MALDI analysis: The reaction mixture (50 µL) contained temp^{1C} (3 µM, 2.5 µL) prim^{rnd} (3 µM, 2.5 µL), dGTP (0.5 mM, 2.5 µL), dC^{Cest}TP (0.5 mM, 5 µL), KOD XL DNA polymerase (1 U) and reaction buffer (10×, 5 µL) as supplied by the manufacturer. The reaction mixture was incubated for 30 minutes at 60 °C. The reaction was stopped by cooling to 4 °C.

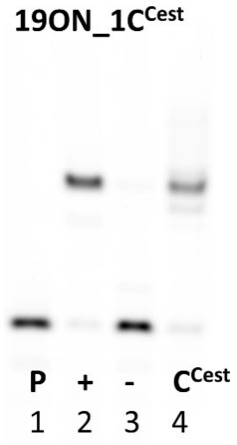

Fig. S4. PAGE analysis of PEX using **dC^{Cest}TP**, 5’-(6-FAM)-labelled prim^{rnd} and Temp^{1C}; [lanes (1) primer; (2) positive control: natural dNTPs; (3) negative control: without dCTP; (4) reaction containing **dC^{Cest}TP**].

2.1.11 Single dA^{DPPE}TP incorporation

Analytical PEX: The reaction mixture (10 µL) contained temp^{1A} (3 µM, 0.75 µL) prim^{rnd} (3 µM, 0.5 µL), dGTP (0.5 mM, 0.2 µL), **dA^{DPPE}TP** (0.1 mM, 1 µL), KOD XL DNA polymerase (0.6 U) and reaction buffer (10×, 1 µL) as supplied by the manufacturer. The reaction mixture was incubated for 30 minutes at 60 °C. The PEX reaction was stopped by addition of PAGE stop solution (10 µL) and heated for 2 minutes at 95 °C. Samples were analyzed by denaturing PAGE. Visualized using fluorescence imaging (**Figure S5**).

Sample for MALDI analysis: The reaction mixture (50 µL) contained temp^{1A} (3 µM, 2.5 µL) prim^{rnd} (3 µM, 2.5 µL), dGTP (0.5 mM, 1 µL), **dA^{DPPE}TP** (0.1 mM, 5 µL), KOD XL DNA polymerase (3 U) and reaction buffer (10×, 5 µL) as supplied by the manufacturer. The reaction mixture was incubated for 30 minutes at 60 °C. The reaction was stopped by cooling to 4 °C.

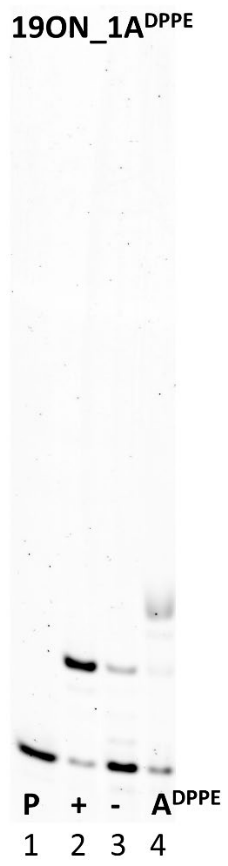

Fig. S5. PAGE analysis of PEX using **dA^{DPPE}TP**, 5’-(6-FAM) labelled prim^{rnd} and Temp^{1A}; [lanes (1) primer; (2) positive control: natural dNTPs; (3) negative control: without dATP; (4) reaction containing **dA^{DPPE}TP**].

2.1.12 Single dT^{DHPE}TP incorporation

Analytical PEX: The reaction mixture (10 µL) contained temp^{1T} (3 µM, 0.75 µL) prim^{rnd} (3 µM, 0.5 µL), dGTP (0.5 mM, 0.2 µL), **dT^{DHPE}TP** (0.1 mM, 1 µL), KOD XL DNA polymerase (0.6 U) and reaction buffer (10×, 1 µL) as supplied by the manufacturer. The reaction mixture was incubated for 45 minutes at 60 °C. The PEX reaction was stopped by addition of PAGE stop solution (10 µL) and heated for 2 minutes at 95 °C. Samples were analyzed by denaturing PAGE. Or the PEX reaction was stopped by cooling to 4 °C. And samples were mixed with a native loading dye and analysed by native PAGE or 4% agarose. The visualization was done using fluorescence imaging (**Figure S6A, C, E**).

Sample for MALDI experiments: The reaction mixture (50 µL) contained temp^{1T} (100 µM, 1 µL), prim^{rnd} (100 µM, 1 µL), dGTP and **dT^{DHPE}TP** (2 mM, 1 µL), and KOD XL DNA polymerase (6.25 U) in

the enzyme reaction buffer (10×, 5 µL) as supplied by the manufacturer. The reaction mixture was incubated for 2 h at 60 °C. The reaction was stopped by cooling to 4 °C.

2.1.13 Single dT^{DHPE}TP incorporation in the terminal position

Sample for electrophoretic analysis, MALDI experiments: The reaction mixture (50 µL) contained temp^{TermT} (100 µM, 1 µL), prim^{rnd} (100 µM, 1 µL), **dT^{DHPE}TP** (4 mM, 2.5 µL), and Therminator DNA polymerase (6 U), enzyme reaction buffer (10×, 5 µL) as supplied by the manufacturer. The reaction mixture was incubated for 2 h at 60 °C.

The PEX reaction was 10× diluted and stopped by addition of PAGE stop solution (10 µL) and heated for 2 minutes at 95 °C. Diluted PEX samples were analyzed by denaturing PAGE. Or the PEX reaction was stopped by cooling to 4 °C. Sample was 10× diluted and mixed with a native loading dye and analysed by native PAGE or 4% agarose. The visualization was done using fluorescence imaging (**Figure S6B, D, F**).

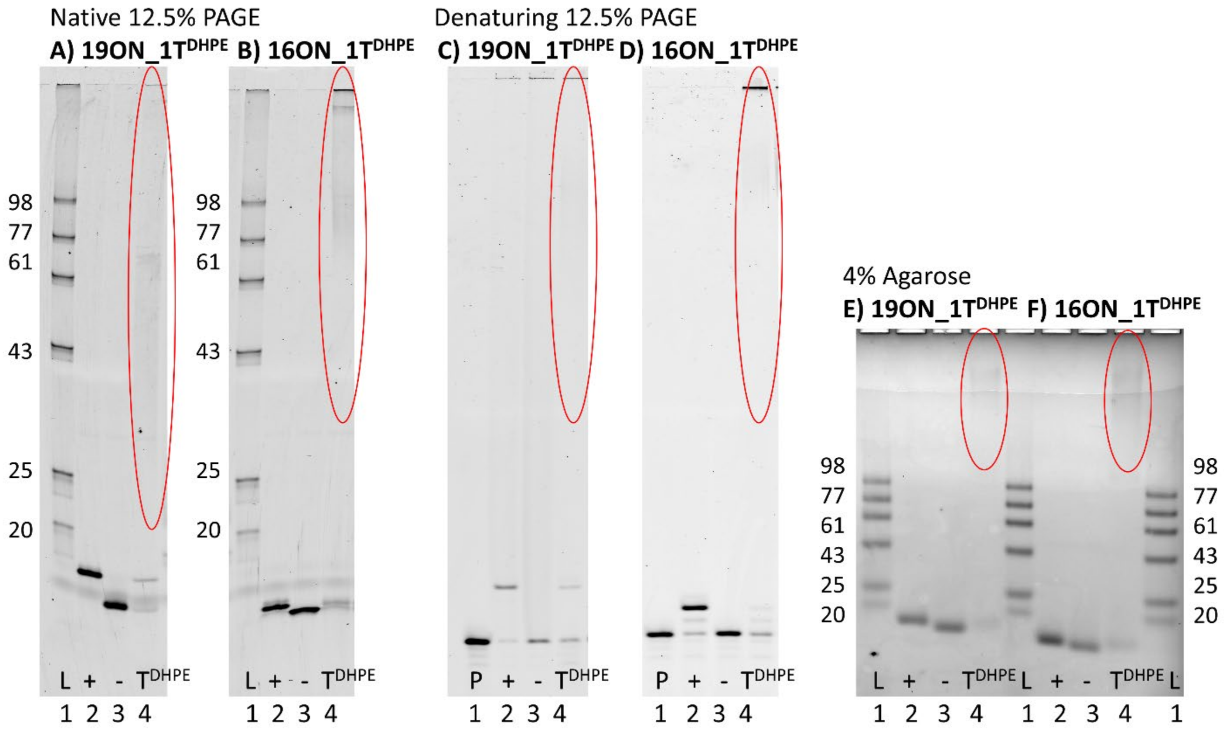

Fig. S6. PAGE analysis of PEX using **dT^{DHPE}TP**, 5'-(6-FAM)-labelled prim^{rnd} and (A, C, E) Temp^{1T}; (B, D, F) Temp^{TermC}; [Lanes(1) primer (denaturing PAGE C and D) or ds ladder (native PAGE A and B and agarose electrophoresis E and F); (2) positive control: natural dNTPs; (3) negative control: without dTTP; (4) reaction containing dT^{DHPE}TP] lightly visible smears suggesting aggregation are designated by red circles (visible when over-exposed).

2.2 Polymerase chain reaction (PCR) study with modified dNTPs – example of tested conditions

The PCR was performed with a C1000Touch thermal cycler (Biorad) using following cycling protocol: 98 °C for 3 min, followed by 30 cycles at 95 °C for 1 min, 53 °C for 1 min, 72 °C for 1 min, followed by final elongation step at 75 °C for 2 min.

The PCR reaction mixture (20 µL) contained KOD XL DNA polymerase (2.5 U/µL, 1.5 µL) corresponding natural dNTPs (4 mM, 0.5 µL), modified **dT^{Pam}TP** or **dC^{Calk}TP** (4 mM, 1 µL), 5'-(6-FAM)-labelled forward primer prim^{LT25TH}, reverse primer prim^{L20-} (10 µM, 4 µL each) and 98-mer template temp^{FVLA} (1 µM, 0.5 µL) in KOD XL reaction buffer (10×, 2 µL) as supplied by the manufacturer. PCR products were analysed by 4% agarose gel in 0.5× TBE buffer. The visualization was done using fluorescence imaging (**Figure S7**).

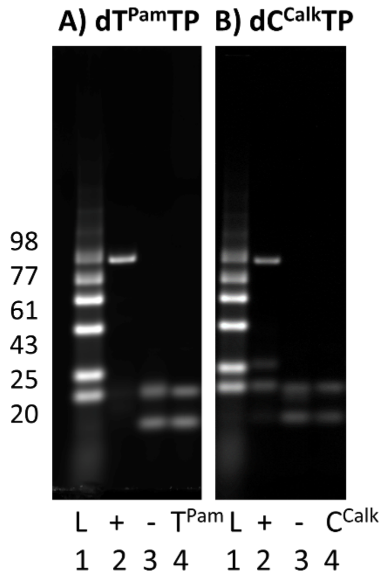

Fig. S7. Agarose gel analysis of PCR synthesis with KOD XL DNA polymerase using 98-mer temp^{FVLA} 5'-(6-FAM)-labelled prim^{LT25TH} and prim^{L20-}; [lanes (1) double stranded ladder; (2) positive control: natural dNTPs; (3) negative control: without studied dNTP (4) PCR using modified (A) **dT^{Pam}TP** or (B) **dC^{Calk}TP**].

2.3 Terminal deoxyribonuleotidyl transferase (TdT) single strand elongation study with modified dNTPs –

2.3.1 Example of tested analytical conditions

The reaction mixture (10 µL) contained prim^{rnd} (3 µM, 0.5 µL), TdT (12 U), natural or modified dNTP (each 2 mM, 1 µL) in TdT reaction buffer (5×, 2 µL) as supplied by the manufacturer. Reaction was incubated at 37 °C for 1 h. The PEX reaction was stopped by addition of PAGE stop solution (10 µL) and heated for 2 minutes at 95 °C. Samples were analyzed by denaturing PAGE and visualized using fluorescence imaging (**Figure S8**).

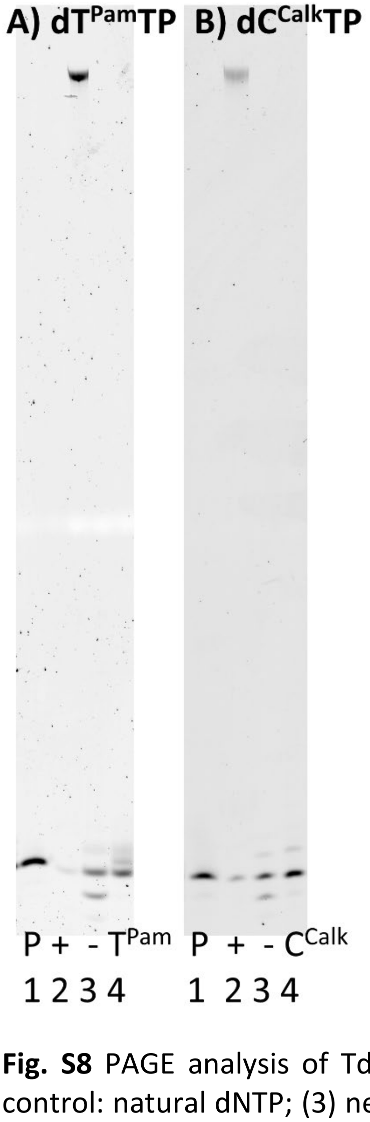

Fig. S8 PAGE analysis of TdT elongation using 15-mer prim^{rnd}; [lanes (1) prim^{rnd}; (2) positive control: natural dNTP; (3) negative control: without dNTP (4) TdT elongation using modified (A) **dT^{Pam}TP** or (B) **dC^{Calk}TP**].

2.3.1 Example of tested preparative conditions and effect of various additives

The reaction mixture (10 µL) contained prim[rnd] (20 µM, 0.5 µL), TdT (20 U), modified dNTP (in a range 0.01 mM to 2 mM, 1 µL; otherwise 0.1 mM, 1 µL) in TdT reaction buffer (5×, 2 µL) as supplied by the manufacturer and additive (5% formaimde, 50% Glycerol, 100% DMSO, or 50 mM solutions; 1 µL). Reaction was incubated at 37 °C for 24 h. The PEX reaction was stopped by addition of PAGE stop solution (10 µL) and heated for 2 minutes at 95 °C. Samples were analyzed by denaturing PAGE and visualized using fluorescence imaging (**Figure S9**).

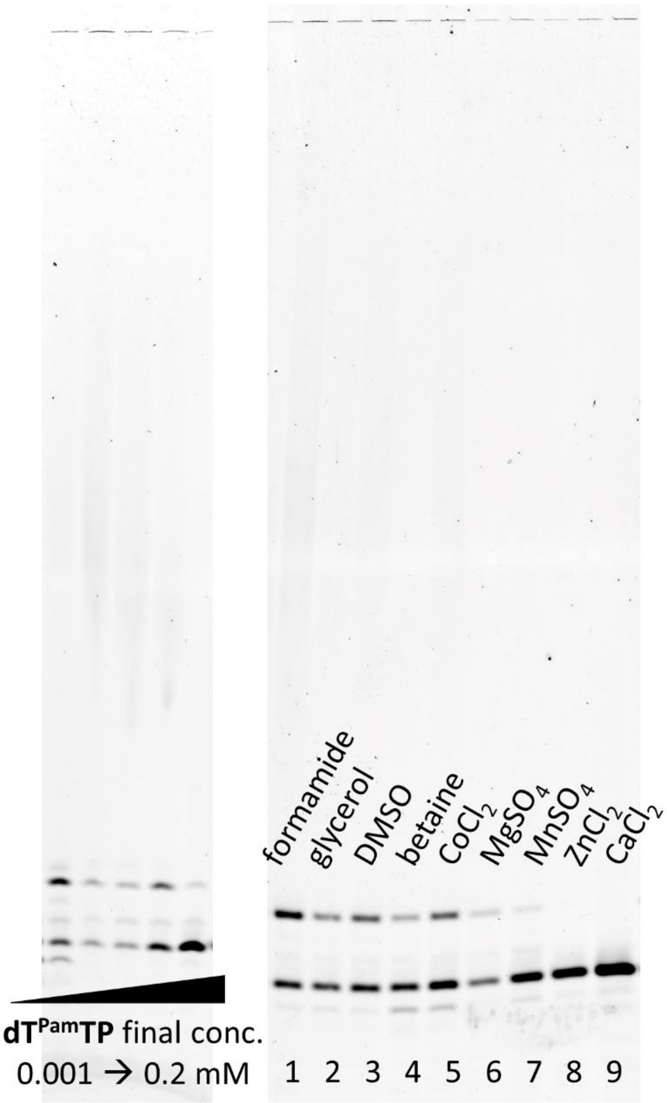

Fig. S9 PAGE analysis of TdT elongation with various **dT**[Pam]**TP** concentrations; or with 0.01 mM final concentration of **dT**[Pam]**TP** and with various additives

2.4 Preparation of newly designed probes for improved membrane anchoring

2.4.1 Oligonucleotides bearing two modified dT^{Pam} (I.)

Sample for analytical PEX, MALDI and *in vitro* experiments: The reaction mixture (50 µL) contained temp^{TCT}, or temp$^{T(1)C(1)T}$, or temp$^{T(2)C(2)T}$ (100 µM, 1 µL), prim^{rnd} (100 µM, 1 µL), corresponding natural dNTPs (4 mM, 1 µL), **dT^{Pam}TP** (2 mM, 1 µL), and KOD XL DNA polymerase (1.25 U, or 3.75 U when using temp^{TCT}) in the enzyme reaction buffer (10×, 5 µL) as supplied by the manufacturer. The reaction mixture was incubated for 2 h at 60 °C. Natural strand standard was prepared under similar conditions using natural dNTPs and 1.25 U KOD XL and incubating for 15 min at 60 °C. The PEX reaction was 10× diluted and stopped by addition of PAGE stop solution (10 µL) and heated for 2 minutes at 95 °C. Diluted PEX samples were analyzed by denaturing PAGE and visualized using fluorescence imaging (**Figure S10A-E**).

2.4.2 Oligonucleotides bearing mixed, multiple modifications (II.)

Sample for analytical PEX, MALDI and *in vitro* experiments: The reaction mixture (50 µL) contained temp^{TCT}, or temp$^{T(1)C(1)T}$, or temp$^{T(2)C(2)T}$, or temp$^{T(3)C(3)T}$, or temp$^{T(5)C(5)T}$ (100 µM, 1 µL), prim^{rnd} (100 µM, 1 µL), corresponding natural dNTPs (4 mM, 1 µL), **dT^{Pam}TP** and **dC^{Cdeg}TP** (each 2 mM, 1 µL) and KOD XL DNA polymerase (3.75 U) in the enzyme reaction buffer (10×, 5 µL) as supplied by the manufacturer. The reaction mixture was incubated for 2 h at 60 °C. Natural strand standard was prepared under same conditions using natural dNTPs and 1.25 U KOD XL and incubating for 15 min at 60 °C. The PEX reaction was 10× diluted and stopped by addition of PAGE stop solution (10 µL) and heated for 2 minutes at 95 °C. Diluted PEX samples were analyzed by denaturing PAGE and visualized using fluorescence imaging (**Figure S10A-E**).

2.4.3 Oligonucleotides bearing two modified dC^{Cdeg} (III.)

Sample for analytical PEX, MALDI and *in vitro* experiments: The reaction mixture (50 µL) contained temp$^{C(5)C}$ (100 µM, 1 µL), prim^{rnd} (100 µM, 1 µL), corresponding natural dNTPs (4 mM, 1 µL) and **dC^{Cdeg}TP** (2 mM, 1 µL) and KOD XL DNA polymerase (3.75 U) in the enzyme reaction buffer (10×, 5 µL) as supplied by the manufacturer. The reaction mixture was incubated for 2 h at 60 °C. Natural strand standard was prepared under same conditions using natural dNTPs and

1.25 U KOD XL and incubating for 15 min at 60 °C. The PEX reaction was 10× diluted and stopped by addition of PAGE stop solution (10 µL) and heated for 2 minutes at 95 °C. Diluted PEX samples were analyzed by denaturing PAGE and visualized using fluorescence imaging (**Figure S10F**).

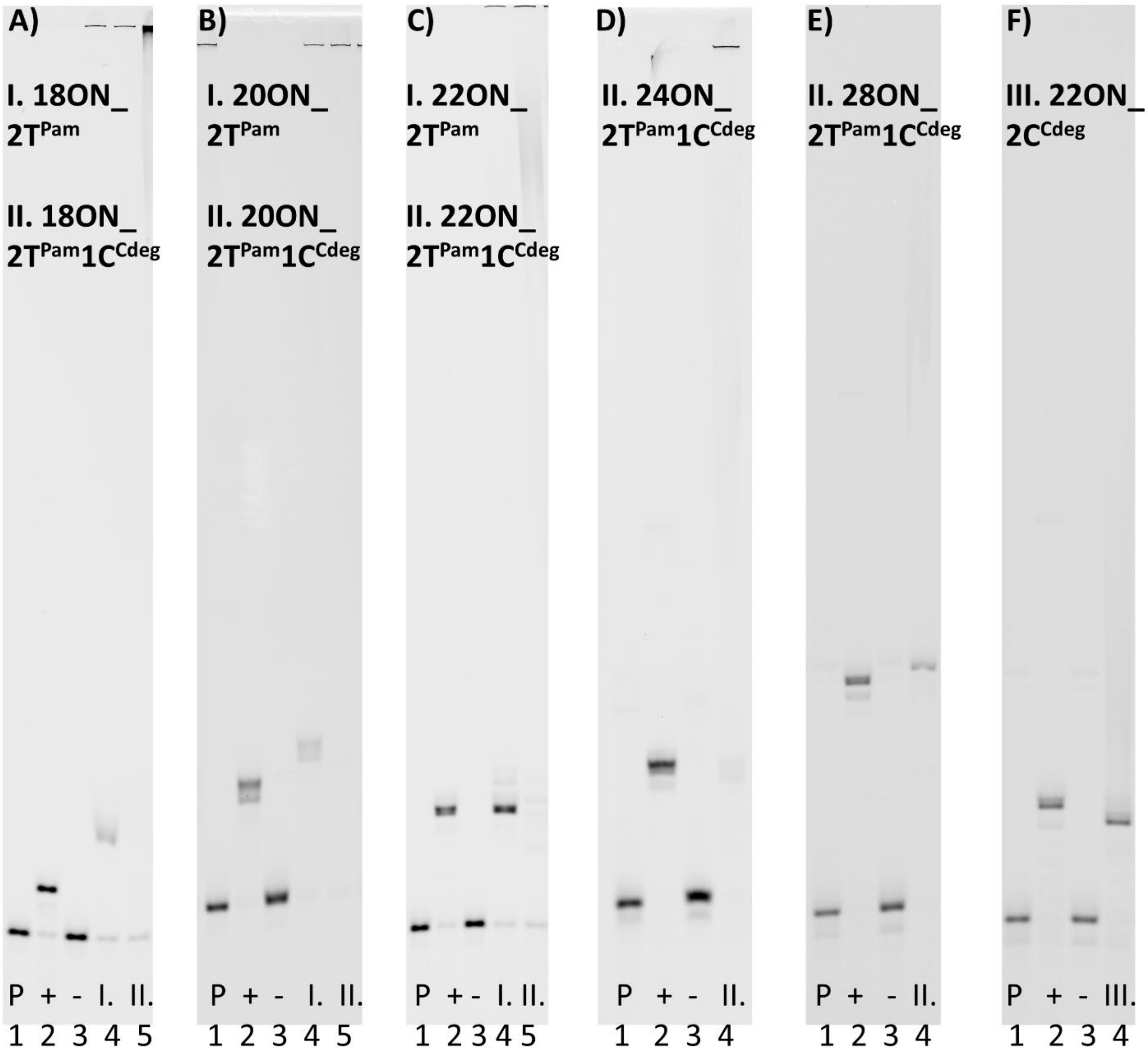

Fig. S10 PAGE analysis of PEX using 5'-(Cy5)-labelled prim^{rnd} and (A) temp^{TCT}, (B) temp$^{T(1)C(1)T}$, (C) temp$^{T(2)C(2)T}$, (D) temp$^{T(3)C(3)T}$, (E) temp$^{T(5)C(5)T}$, (F) Temp$^{C(5)C}$; [lanes (1) primer; (2) positive control: natural ON; (3) negative control: annealed primer and template; (4) and (5) reactions containing modified nucleotide(s): (I) **dT^{Pam}TP** (II) **dT^{Pam}TP** + **dC^{Cdeg}TP** (III) **dC^{Cdeg}TP**].

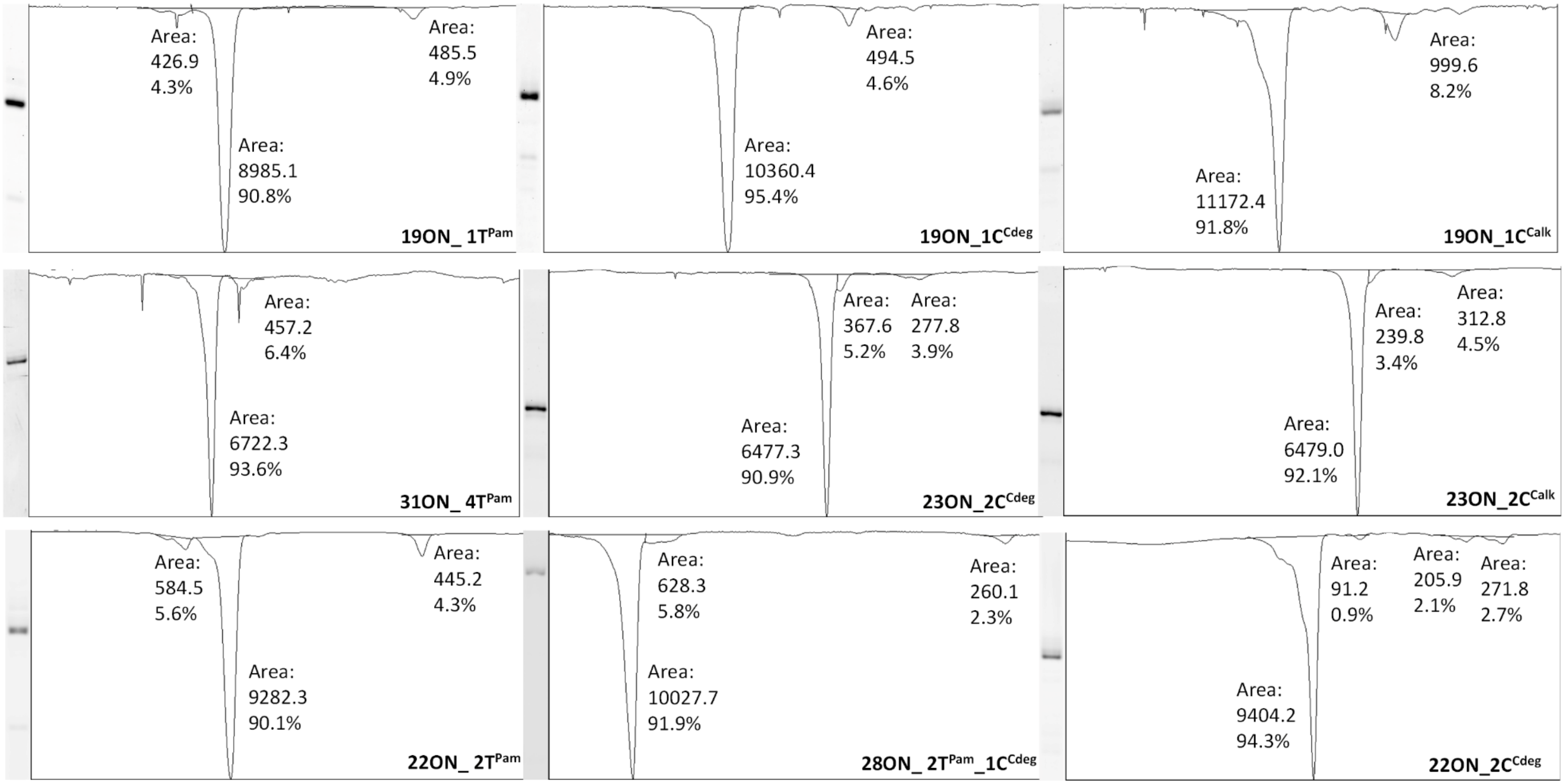

Fig. S11 PAGE analysis of PEX products with subsequent quantification using ImageJ software. The image analysis suggests PEX product purity ≥90%

2.5 Ester bond stability assessment

2.5.1 Incubation of dC^{Cest}TP

Two parallel reaction mixtures (50 µL) contained modified **dC^{Cest}TP** (0.5 mM, 5 µL) either with or without KOD XL DNA polymerase (2.5 U) in reaction buffer (10×, 5 µL) as supplied by the manufacturer. The reaction mixture was incubated for 2 h at 60 °C. The reaction was stopped by cooling to 4 °C. The reaction mixture (10 µL) was injected on UPLC-MS Agilent 1290 Infinity II bio system with DAD and LC/MSD XT MS (operating in negative mode) detectors equipped with Acquity Premier BEH C8 1.7 µm 100×2.1 mm column (linear gradient of 12 mM Et_3N and 300 mM HFIP in H_2O to 12 mM Et_3N and 300 mM HFIP in MeOH in 15 min to 10 mM ammonium formate in MeOH with subsequent reequlibration). **Fig. S12**

2.5.2 Incubation of dA^{DPPE}TP

Two parallel reaction mixtures (50 µL) contained modified dNTP **dA^{DPPE}TP** (0.5 mM, 5 µL) either with or without KOD XL DNA polymerase (2.5 U) in reaction buffer (10×, 5 µL) as supplied by the manufacturer. The reaction mixture was incubated for 2 h at 60 °C. The reaction was stopped by cooling to 4 °C. The reaction mixture (10 µL) was injected on UPLC-MS Agilent 1290 Infinity II bio system with DAD and LC/MSD XT MS (operating in negative mode) detectors equipped with Acquity Premier BEH C8 1.7 µm 100×2.1 mm column (linear gradient of 12 mM Et_3N and 300 mM HFIP in H_2O to 12 mM Et_3N and 300 mM HFIP in MeOH in 15 min to 10 mM ammonium formate in MeOH with subsequent reequlibration). **Fig. S13**

Ester bond was cleaved in pure water upon heating for 2 h at 60 °C. This was not intensified by presence of the DNA polymerase. Notably, heating of both dNTPs in buffered solution resulted into a more significant decomposition of triphosphate to diphosphate and/or monophosphate.

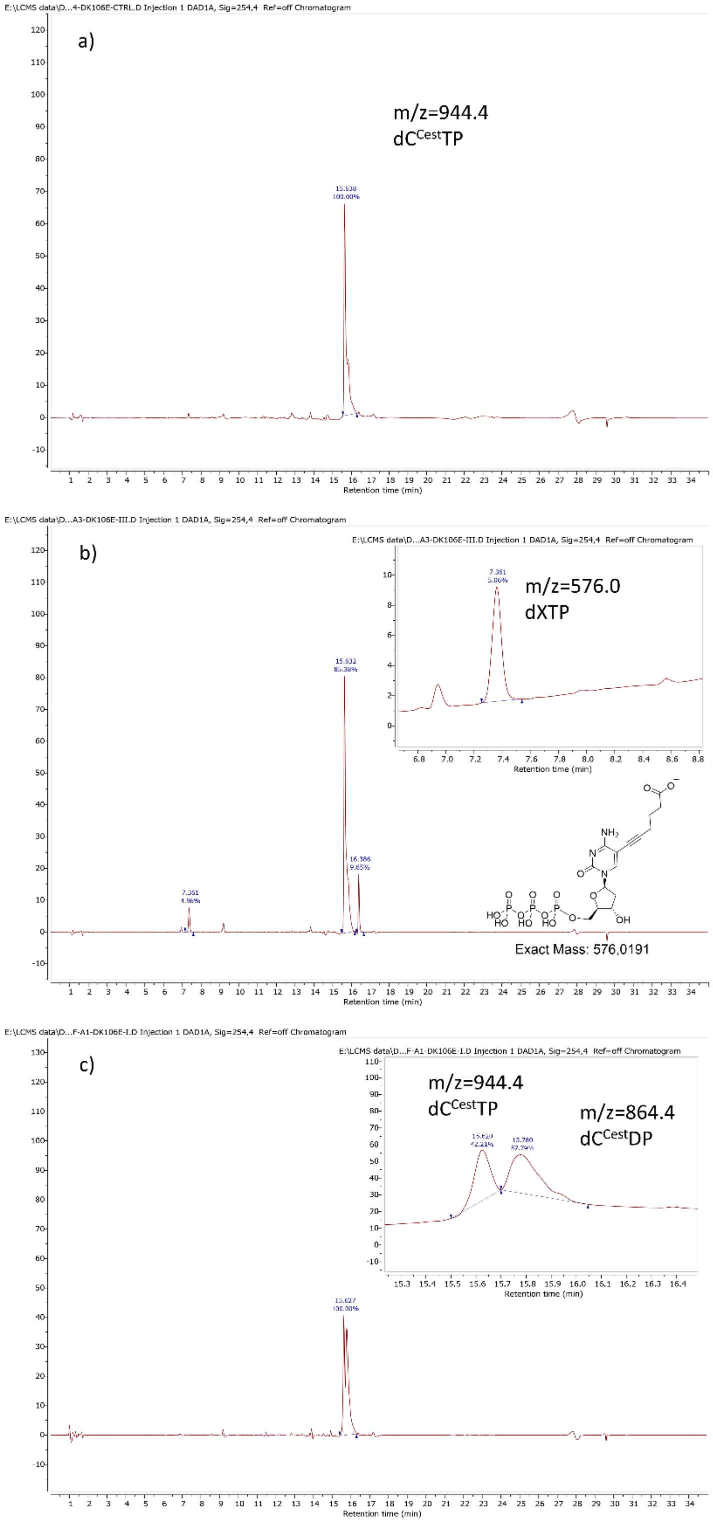

Fig. S12 dC^{Cest}TP ester bond stability assessment (**a**) control: **dC^{Cest}TP**; (**b**) **dC^{Cest}TP** incubated for 2 h at 60 °C in H_2O (**c**) **dC^{Cest}TP** incubated for 2 h at 60 °C with enzyme in the enzyme reaction buffer; appearance of the peak (m/z=576.0) at 7.4 min upon heating in H_2O (**b**) suggests low stability in aqueous environment, which is not intensified by DNA polymerase presence (**c**)

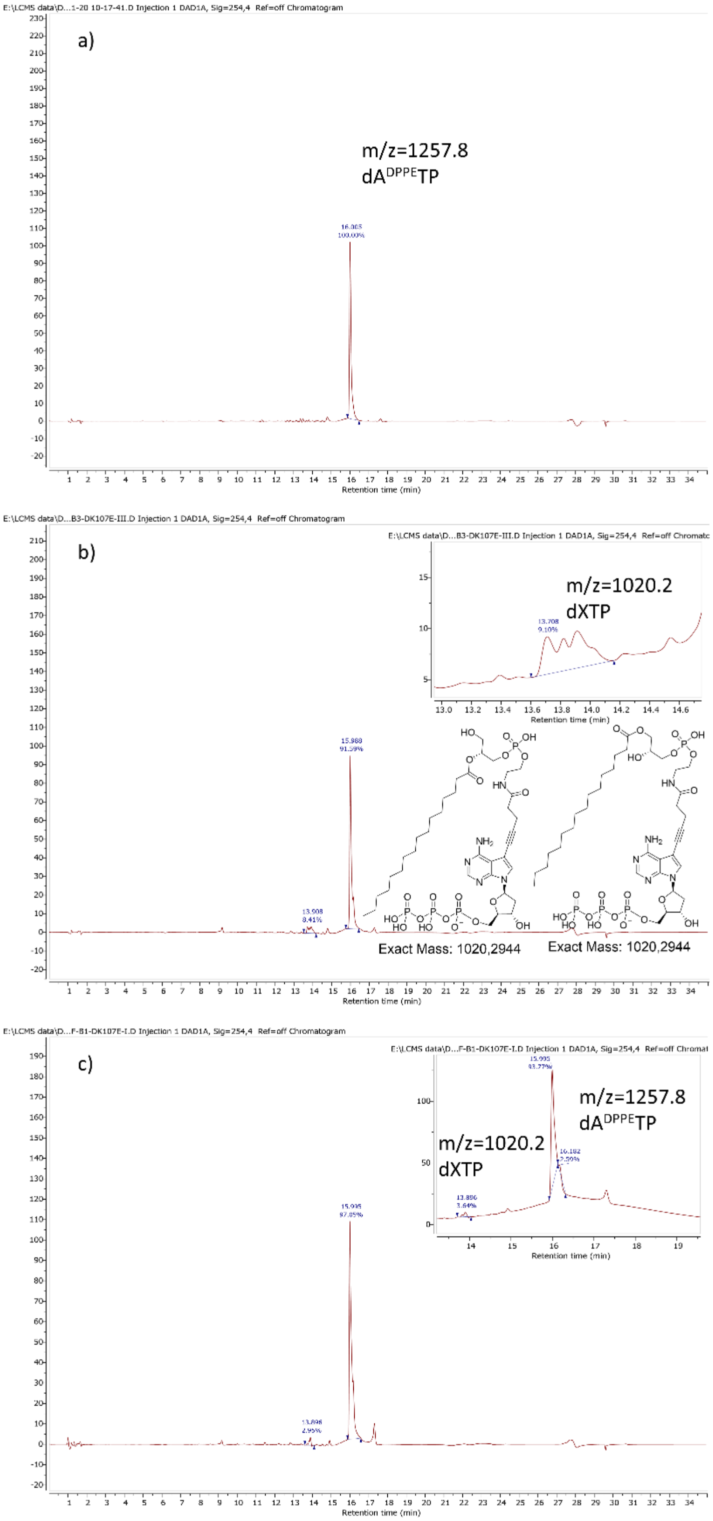

Fig. S13 dA^{DPPE}TP ester bond stability assessment (**a**) control: **dA^{DPPE}TP**; (**b**) **dA^{DPPE}TP** incubated for 2 h at 60 °C in H_2O (**c**) **dA^{DPPE}TP** incubated for 2 h at 60 °C with enzyme in the enzyme reaction buffer; appearance of the peak (m/z=1020.2) at 13.8 min upon heating in H_2O (**b**) suggests low stability in aqueous environment, which is not intensified by DNA polymerase presence (**c**)

2.6 Magnetoseparation procedure

50 µL of Streptavidin magnetic beads (SMB) were washed three times with 200 µL of Binding buffer TEN100 (10 mᴍ Tris, 1 mᴍ EDTA, 100 mᴍ NaCl, pH 7.5). The PEX reaction mixture was added to 100 µL of pre-washed SMB in TEN100 (1:2) and incubated for 30 minutes (15 °C, 1200 rpm). SMB were captured on magnet (DynaMag-2, Invitrogen), washed successively three times with 200 µL of wash buffer TEN500 (10 mM Tris, 1 mM EDTA, 500 mM NaCl, pH 7.5) and three times with 200 µL of water. Modified strand was then released by denaturation in 50 µL of hot water (incubation for 4 minutes, 72 °C, 900 rpm), SMB bearing template strand were immediately captured on magnet and solution containing modified strand was taken. Sample was evaporated prior MALDI-TOF measurement. 5 uL of 10× PBS were added For *in vitro* cell treatment, product stored at 8 °C and used within 48 h.

2.7 MALDI-TOF measurements

The MALDI-TOF spectra were measured on a MALDI-TOF/TOF mass spectrometer with a 1 kHz smartbeam II laser. The measurements were done in reflectron mode by a droplet technique, with the mass range up to 30 kDa. The matrix consisted of 3-hydroxypicolinic acid (HPA)/picolinic acid (PA)/ammonium tartrate in ratio 9:1:1. The matrix (1 µL) was applied on the target (ground steel) and dried down at 22 °C. The sample (1 µL) and matrix (1 µL) were mixed and added on top of the dried matrix preparation spot and dried down at 22 °C. Copies of all MS spectra can be seen in section 3.

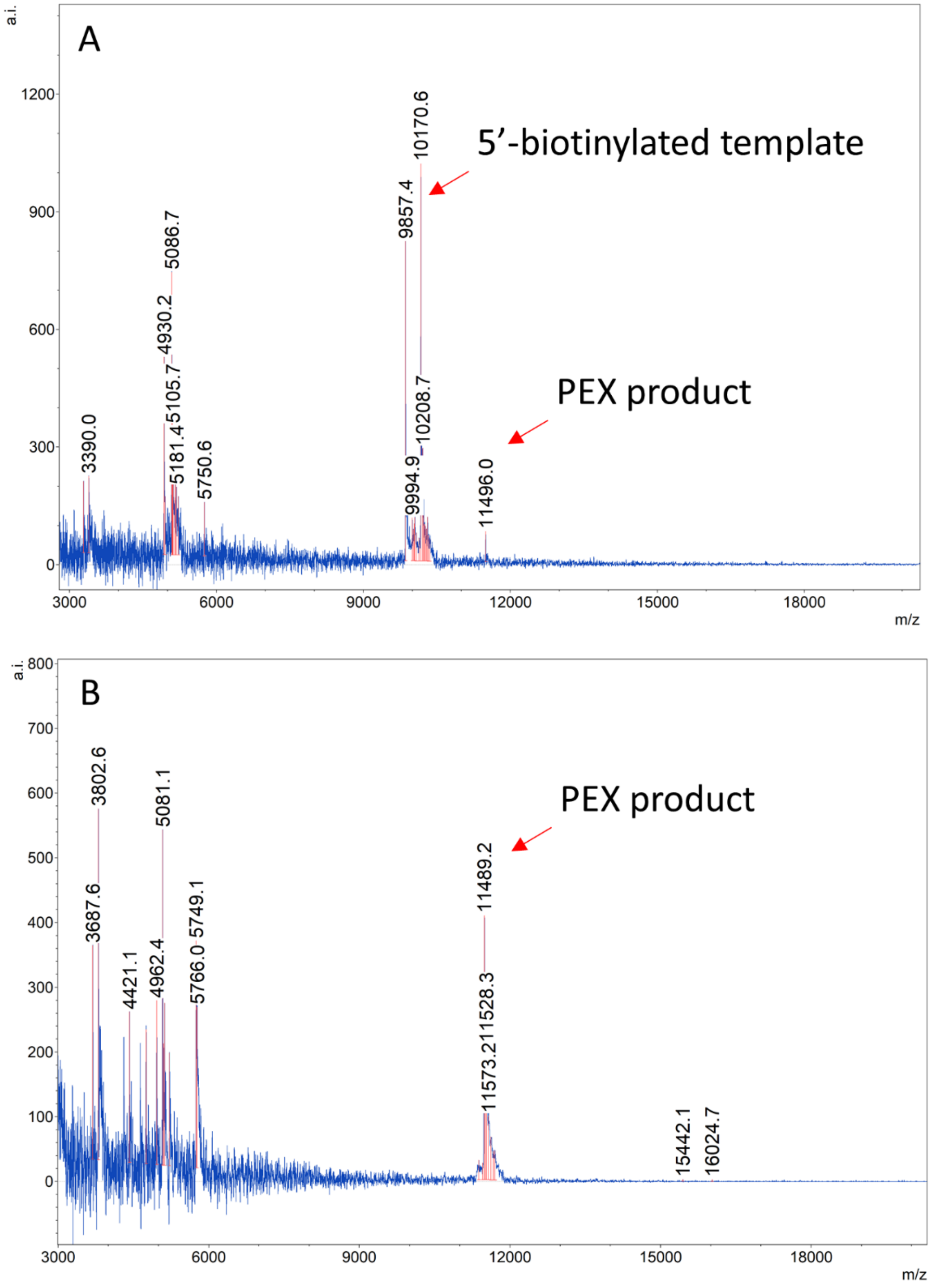

Fig. S14 Comparison of MALDI spectra of 31ON_4T^{Pam} purified using either (**A**) 5'-biotin or (**B**) 5'-dualbiotin (bottom) template.

2.8 Cell cultures and cultivation conditions

For cell-based experiments two suspension and two adherent cell lines were used. Suspension CCRF-CEM cell line (cat. no. ATCC-CCL-119, ATCC, USA) and HL-60 (cat. no. ATCC-CCL-240, ATCC, USA) were cultured in RPMI-1640 media (cat. no. R8758, Sigma-Aldrich, USA). Adherent U-2 OS cell line (cat. no. ACC-785, DSMZ) was cultured in DMEM high glucose 4.5g/L media (cat. no. R8758, Sigma-Aldrich, USA) and HeLa S3 cell line (cat. no. ATCC-CLL-2.2, ATCC, USA) was cultured in RPMI-1640 media (cat. no. R7638, Sigma-Aldrich, USA). The media were supplemented with 10 % Fetal bovine serum (cat. no. F7524, Sigma-Aldrich, USA; inactivated at 56 °C for 30 minutes), 1.0 % of L glutamine (cat. no. G7513, Sigma-Aldrich, USA) and 1.0 % of Penicillin-Streptomycin (cat. no. P0781, Sigma-Aldrich, USA). Cells were cultured in an incubator (37°C, 5% CO_2). For dissociation of the adherent cells Trypsin-EDTA solution (cat. no. T4049-500 mL, Sigma-Aldrich, USA) was used.

2.9 Live-cell imaging of the anchoring of LONs into cells by confocal microscopy

The cells were cultured under conditions specified above. 24 hours prior to experiments, the cells were seeded into 96 Well Glass Bottom Plate (Cellvis, P96-1.5H-N) in this manner: i) suspension cells (HL-60, CCRF-CEM) were transferred to the complete Leibowitz L-15 medium (10% FCS) and seeded into wells, in which the bottom glass had been pre-coated (2 hours at 37°C) with poly-D-lysine hydrobromide (cat. no. P6407, Sigma-Aldrich), at the density of 50k per well in 100 µL of complete L15 medium; ii) adherent cells (U-2 OS, HeLa S3) were transferred to the complete Leibowitz L-15 medium (10% FCS) and plated to glass bottom wells at densities of 40k and 60k of U-2 OS and HeLa S3 cells, respectively, per well.

On the day of the experiment, the cells were placed into an incubator (37°C, no additional CO_2) of the confocal microscope. The cells were gently washed with PBS and treated with 35 µL of 1 µM LON in PBS and incubated for 10 min at 37 °C. After this period, the LON solution was removed and the cells were placed in pure in L-15 media prior to confocal microscopy imaging was performed at specified time intervals (0 min, 4 h, 24 h). The samples were irradiated with a laser beam being set at 639 nm, the detector (GaAsP) range was set to 640 - 694 nm. For the

two-color experiment were the samples additionally irradiated with a laser beam being set at 493 nm, the GaAsP range was set to 490 – 579 nm. Negative control experiment was carried out with untreated cells to check for autofluorescence under the same settings. The images were taken and exported in Zeiss Imaging Software ZEN 3.2 (blue edition). Images were further processed using ImageJ v1.53t Software for the purposes of two-color quantitative image analysis using the following approach:

The image was split into two channels (green channel – **CMG** image and red channel **LON** image using FIJI routine procedure and converted into 8 bit format.

Processing procedure for **CMG** image:

1. Noise filtering (Gaussian blurr, rad = 1 pixel)
2. Subtract background (sliding paraboloid, d = 10)
3. Autotrashold (mean)
4. Divide 255 = **MASK 1**

Processing procedure for **LON** image:

1. Noise filtering (Gaussian blurr, Sigma = 1 pixel)
2. Subtract background (constant value based on mean value of background noise) **PREPROCESED LON 1**
3. Treshold 0-254
4. BINARY – CONVERT TO MASK (inverts intensities ≥ 254 to 0)
5. Divide 255 = **MASK 2** (Overexposed pixels)
6. **MASK 2** MULTIPLY **PREPROCESED LON 1 = PREPROCESED LON 2** (removes overexposed pixels)

Selection and integration of fluorescence in the intracellular space

1. **MASK 1** , apply „Analyse Particles“ (size 20 µm to infinity, circularity 0-1, Show bare outlines, Exclude on edges; produces new image with outlines of intracellular spaces)
2. apply „File Holes“
3. apply „Binary / Convert to mask“ (produces new image with intracellular spaces complementary to membranes)
4. apply „Invert“
5. apply „Math / DIVIDE 255“ (produces **MASK 4**)
6. apply „Image Calculator“ : **PREPROCESED LON 2** „Multiply“ **MASK 4** = **INTERNALIZED LON** (returns an image of intracellular spaces with fluorescence intensities of internalized LON)
7. apply „Measure“ **INTERNALIZED LON** (raw integrated fluorescence intensities of all pixels)

Selection and integration of fluorescence in the plasma membrane

1. apply „Image Calculator" : **MASK 1** „multiply" **PREPROCESED LON 2** = **MEMBRANES** (returns an image of plasma membrane area with fluorescence intensities of **LON**)
2. Merge **MEMBRANES** and **INTERNALIZED LON:** apply „Merge channels" **MEMBRANES 1** and **INTERNALIZED LON**; visually inspect for unpaired (unfilled) membranes and delete them from **MEMBRANES** image = **MEMBRANES PAIRED**; (this procedure produces image in which each membrane has its own counterpart in **INTERNALIZED LON** image)
3. apply „Measure" **MEMBRANES PAIRED**

Table S2 Acquisition parameters for confocal microscopy imaging

		Laser power (%)	Detector gain (V)	Digital offset	Dig. gain	Line / fr. acc.
Table 1, A)	natural ON	1.20	750	0	1	-
Table 1, A)	**19ON_1T^{Pam}**	0.80	820	0	1	-
Table 1, A)	**16ON_1T^{Pam}**	0.20	860	0	1	-
Table 1, A)	**19ON_1C^{Cdeg}**	0.90	820	0	1	-
Table 1, A)	**16ON_1C^{Cdeg}**	0.70	850	0	1	-
Table 1, A)	**19ON_1C^{Calk}**	0.90	810	0	1	-
Table 1, A)	**16ON_1C^{Calk}**	0.80	850	0	1	-
Table 1, A)	**23ON_2C^{Cdeg}**	0.70	750	0	1	-
Table 1, A)	**31ON_4T^{Pam}**	0.70	750	0	1	-
Table 1, B)	**18ON_2T^{Pam}**	2.80	900	0	1	-
Table 1, B)	**20ON_2T^{Pam}**	2.80	900	0	1	-
Table 1, B)	**22ON_2T^{Pam}**	0.40	800	0	1	-
Table 1, B)	**22ON_2T^{Pam}1C^{Cdeg}**	3.00	780	0	1	-
Table 1, C)	**24ON_2T^{Pam}1C^{Cdeg}**	0.70	750	0	1	-
Table 1, C)	**28ON_2T^{Pam}1C^{Cdeg}**	0.70	750	0	1	-
Table 1, C)	**22ON_2C^{Cdeg}**	0.70	750	0	1	-
Fig. 3	all images	5.00	750	0	1	-
Fig. 4, S16, S17	all images	1.00 (488 nm)<br>2.40 (639 nm)	750	487<br>1956/16-bit	1	-
Fig. 6, S18	U2OS all images	0.20	750	0	1	-
Fig. 6, S18	HeLa S3 all images	0.20	750	0	1	-
Fig. 6, S18	CEM incubation	0.20	750	0	1	-
Fig. 6, S18	CEM resuspended	3.50	750	0	1	-

2.10 Cell membrane persistence study of LONs using flow cytometry

For flow cytometry experiments HL-60 cell line was used. The cells were counted using cell counter (Luna-FL™ Dual Fluorescent Cell Counter, Logos Biosystems, South Korea), washed with PBS and aliquoted (6 x 10^6 cells per 1.5 mL Eppendorf tube). The pellets were treated with 75 µL of 1 µM LON in PBS for 10 minutes at 37 °C, washed 3x in PBS, resuspended in L-15 media and cultivated in an Eppendorf thermoblock at 37 °C. The fluorescence intensities were measured at several time points (0, 15, 30 min, 1, 2, 4, and 24 h). Sample flowrate was 60 µL/min and approximately 200 events/sec. We collected a minimum of 5,000 cells out of life cells gated population (FSC-A vs. SSC-A) and single cells gated population (FSC-A vs. FSC-H). A histogram was created for each measurement using the red laser 638 nm with detection filters 660/10-nm band pass (channel R660-APC-A). Obtained data were analyzed using the FlowJo™ v10.6.1 Software (Becton, Dickinson & Company) and exported data evaluated in GraphPad Prism v.8.4.3. (686) Software (La Jolla, USA).

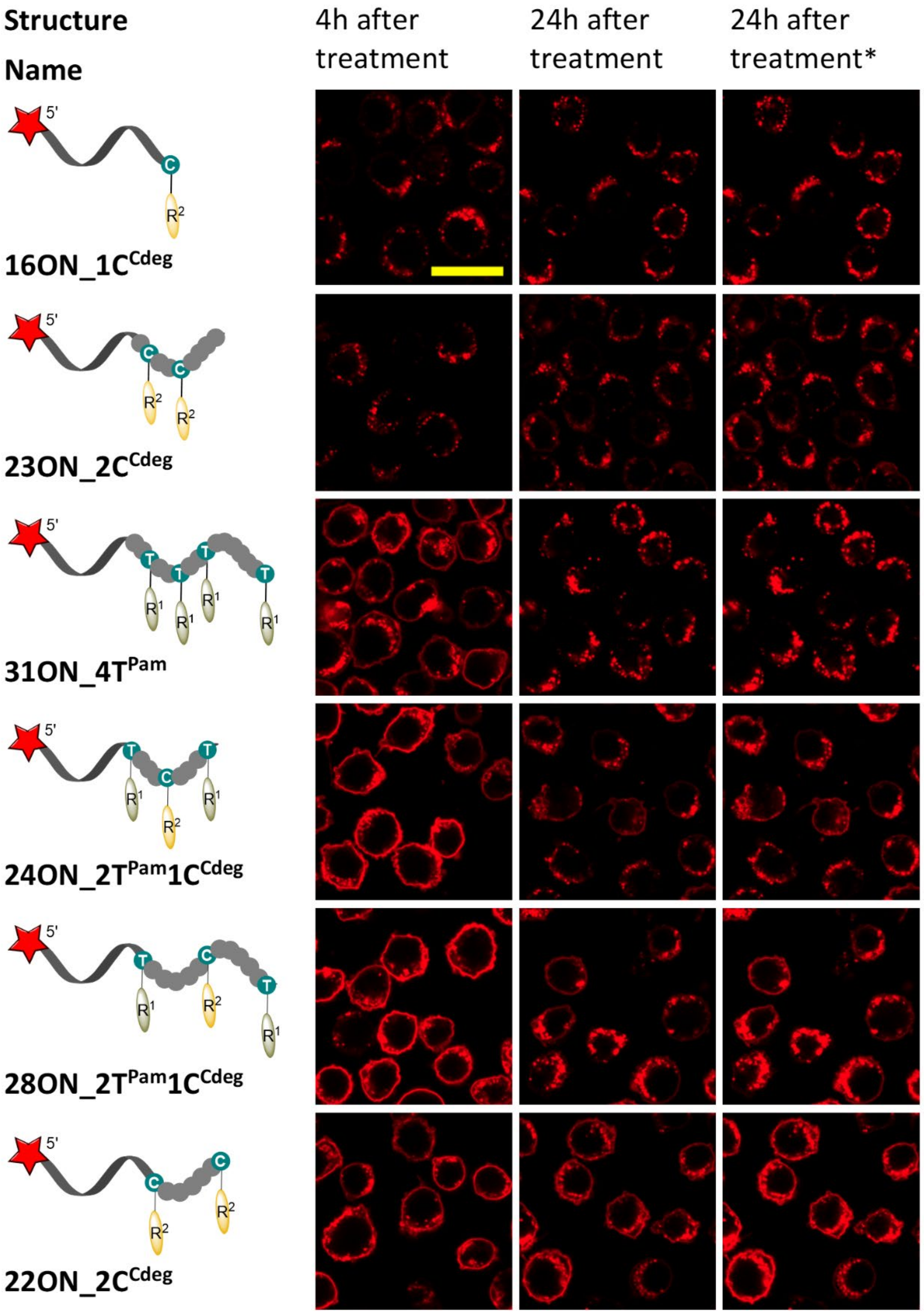

Fig. S15 Fluorescence microscopy of HL-60 cells within 4 h and 24 h time-points after 10 min incubation with 1 µM of given LON followed by incubation at 37 °C pure in L-15 media. Microscopy images were taken under identical settings to illustrate different LON behavior. The yellow marker size is 20 µm. *equally overexposed images

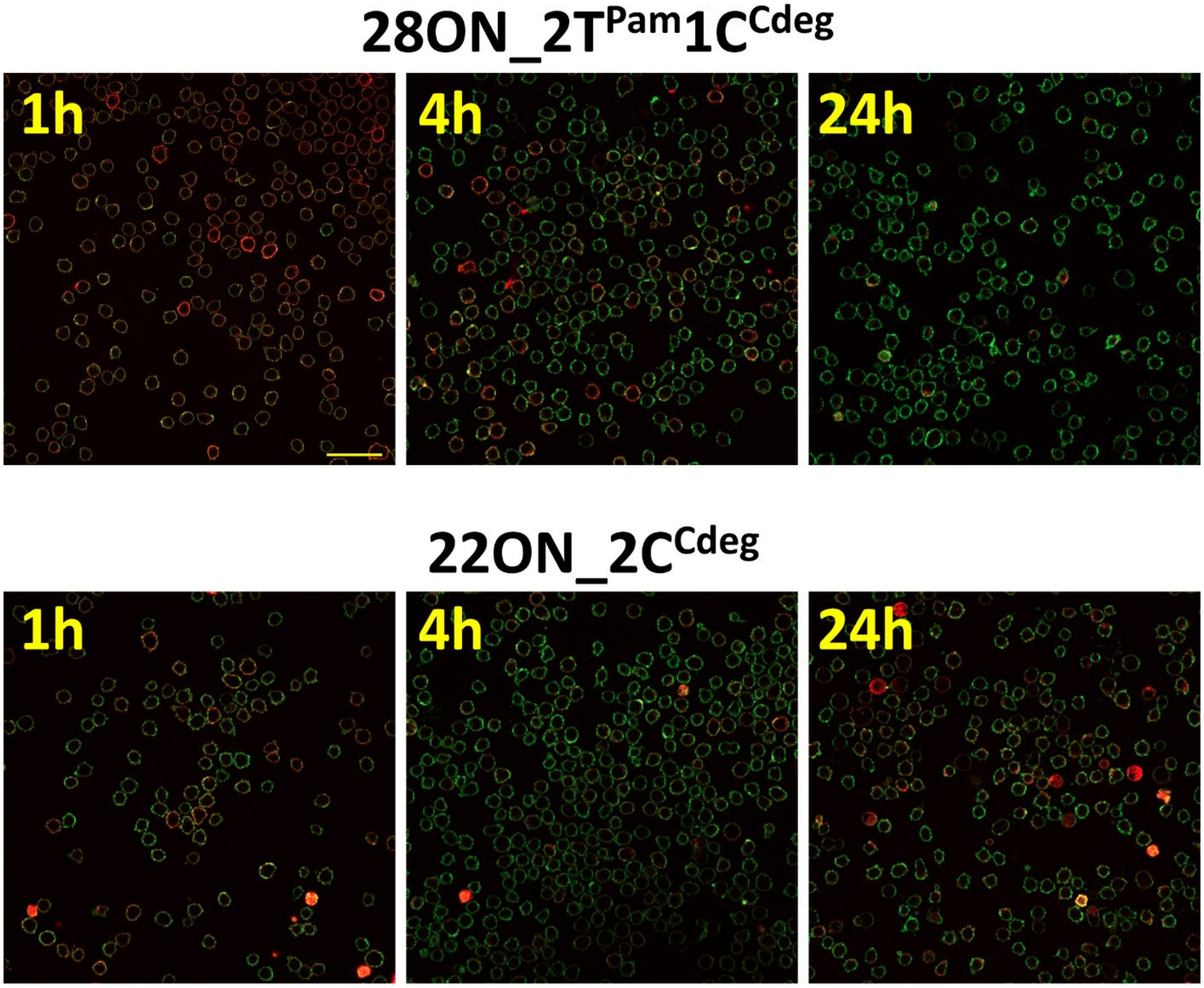

Fig. S16 Confocal microscopy images of the samples of HL-60 cells treated with CellMask™ Green at 1 h, 4 h and 24 h time-points from the LON probe solution removal (**28ON_2T^{Pam}1C^{Cdeg}** (top) and **22ON_2C^{Cdeg}** (bottom)). Images used for the quantitative image analysis.

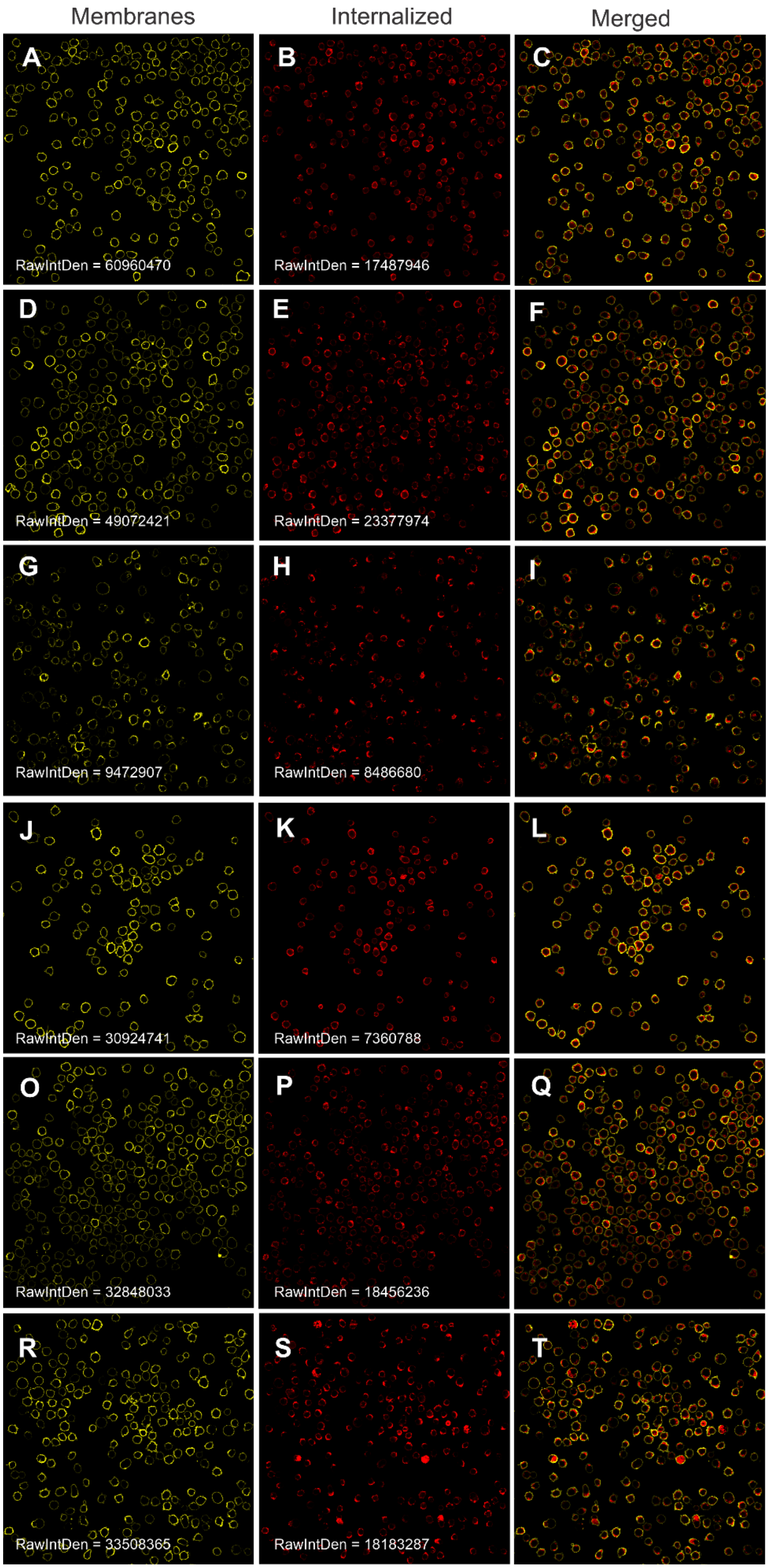

Fig. S17 Quantitative image analysis of the two-color experiment with indicated raw integration densities as calculated with FIJI. Images were generated after processing the data as described in ESI paragraph 2.9.

28ON_2T$^{\mathbf{Pam}}$1C$^{\mathbf{Cdeg}}$, 1 hour (A-C);
28ON_2T$^{\mathbf{Pam}}$1C$^{\mathbf{Cdeg}}$, 4 hours (D-F);
28ON_2T$^{\mathbf{Pam}}$1C$^{\mathbf{Cdeg}}$, 24 hour (G-I);

22ON_2C$^{\mathbf{Cdeg}}$, 1 hour, (J-L);
22ON_2C$^{\mathbf{Cdeg}}$, 4 hours, (O-Q);
22ON_2C$^{\mathbf{Cdeg}}$, 24 hours, (R-T).

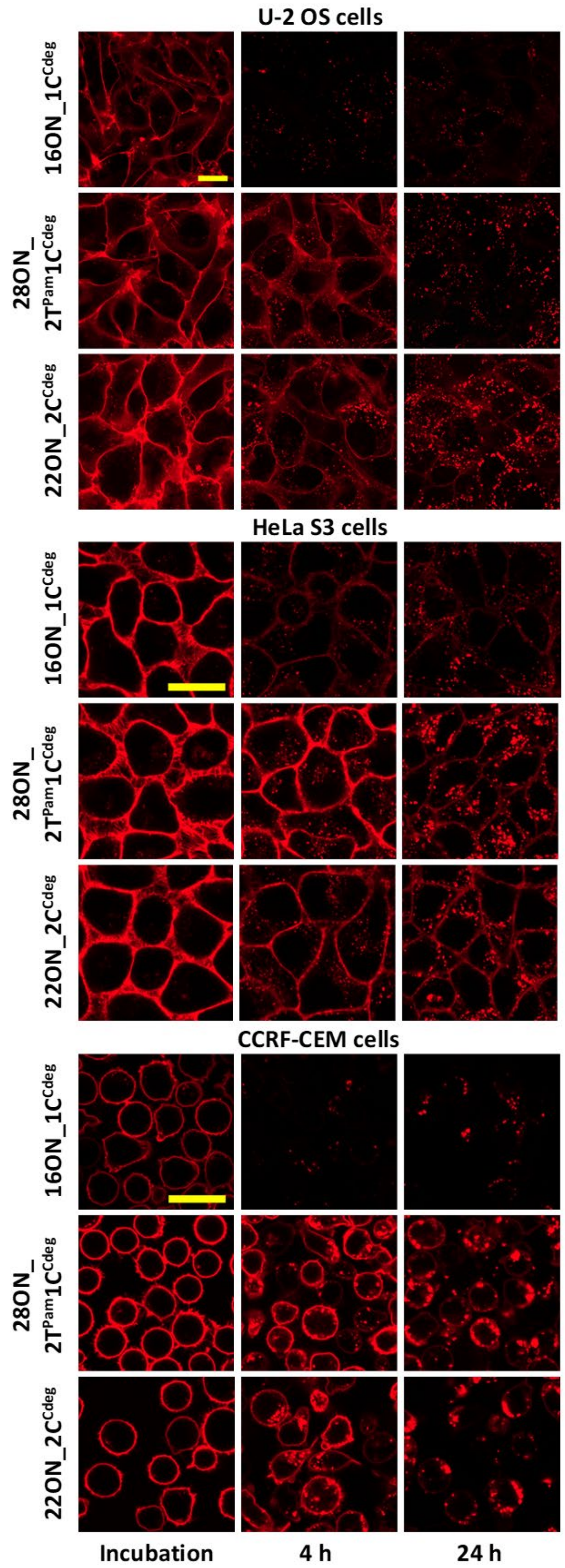

Fig. S18 ON treatment and membrane persistence among various cell lines The yellow marker size is 20 µm. The microscopic images were taken under the same settings and processed under the identical conditions within each cell line.

2.11 DNase I protection assay

To the 4×10 µL of a solution of **natural ON**, **16ON_1C^{Cdeg}**, **22ON_2C^{Cdeg}**, **23ON_2C^{Cdeg}**, **31ON_4T^{Pam}**, **24ON_2T^{Pam}1C^{Cdeg}** and **28ON_2T^{Pam}1C^{Cdeg}** was added DNase I (0.1 U, 1 µL) in the enzyme reaction buffer (10×, 1 µL) as supplied by the manufacturer. The reaction mixtures were incubated for various time periods (0, 10, 30, 60, 120 min) at 37 °C stopped by an addition of EDTA (50 mM, 1 µL) and thermal denaturation for 2 minutes at 95 °C. Samples were mixed with 2× PAGE stop (1:1) solution, analyzed by denaturing PAGE and visualized using fluorescence imaging (**Figure S19**).

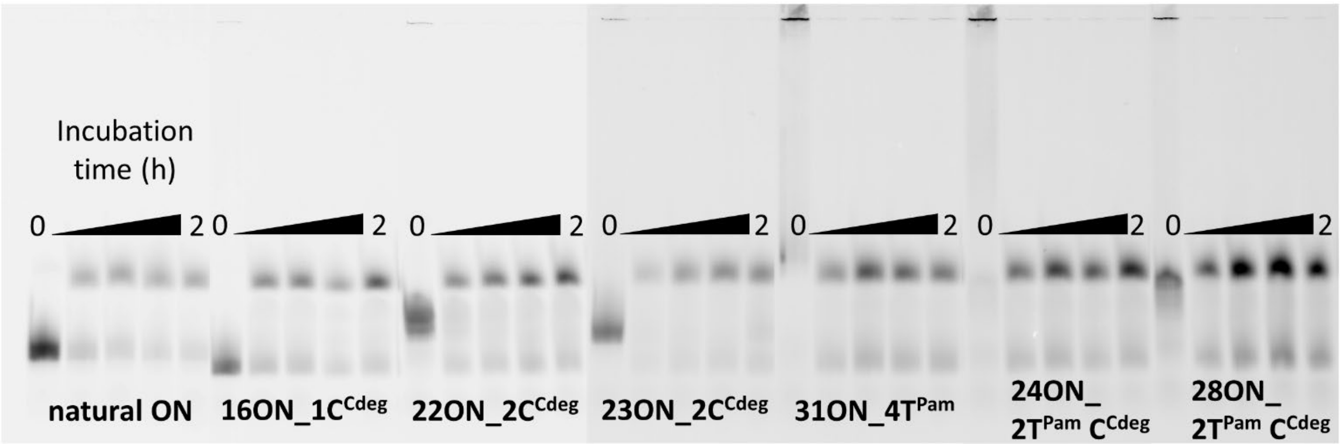

Fig. S19 PAGE analysis of ON samples incubated with DNase I at 37 °C at various time-points.

3. Copies of MALDI-TOF MS spectra

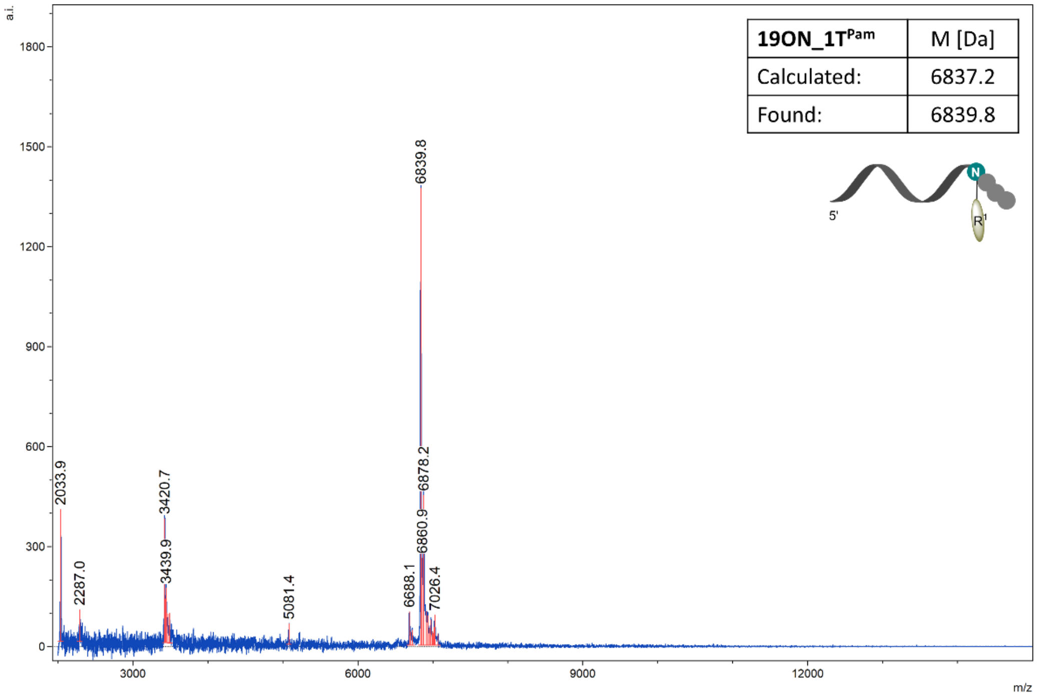

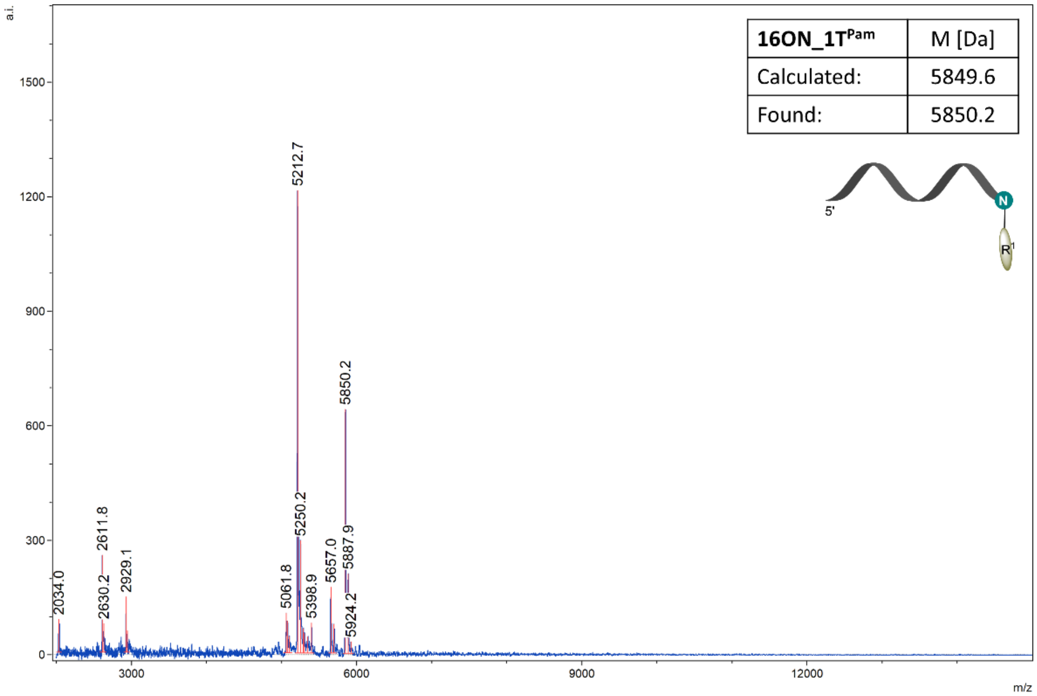

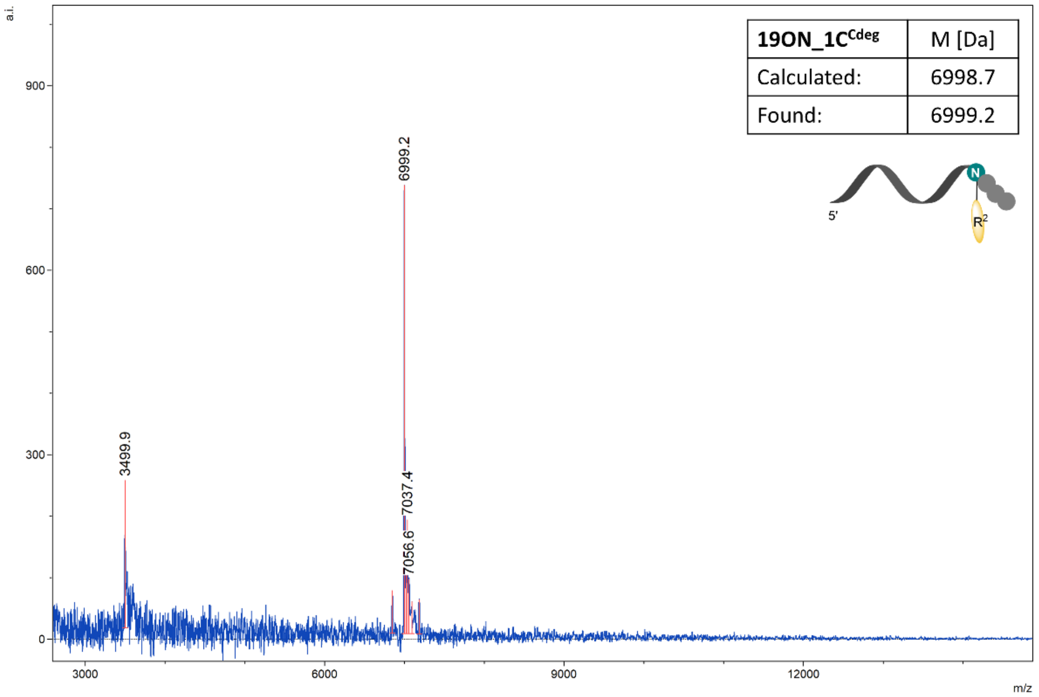

19ON_1C^Cdeg | M [Da]
Calculated: | 6998.7
Found: | 6999.2
5'
N
R2
a.i.
900
600
300
0
3000
6000
9000
12000
m/z
6999.2
3499.9
7037.4
7056.6

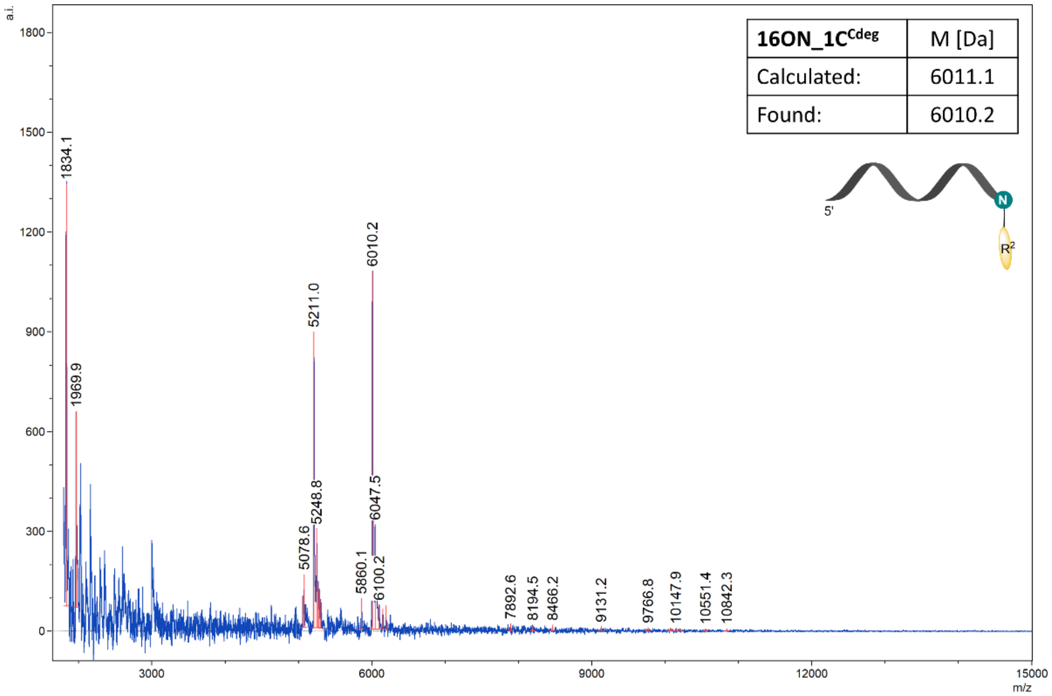

16ON_1C^Cdeg | M [Da]
Calculated: | 6011.1
Found: | 6010.2
5'
N
R2
a.i.
1800
1500
1200
900
600
300
0
3000
6000
9000
12000
15000
m/z
1834.1
1969.9
5211.0
6010.2
5248.8
6047.5
5078.6
5860.1
6100.2
7892.6
8194.5
8466.2
9131.2
9766.8
10147.9
10551.4
10842.3

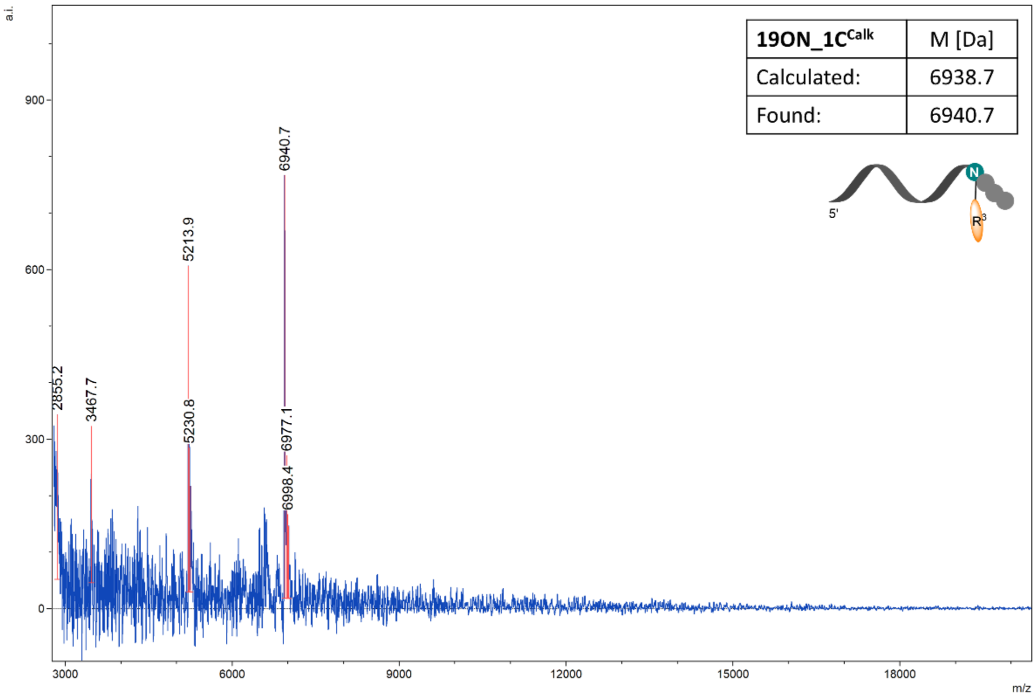

19ON_1C^{Calk}	M [Da]
Calculated:	6938.7
Found:	6940.7

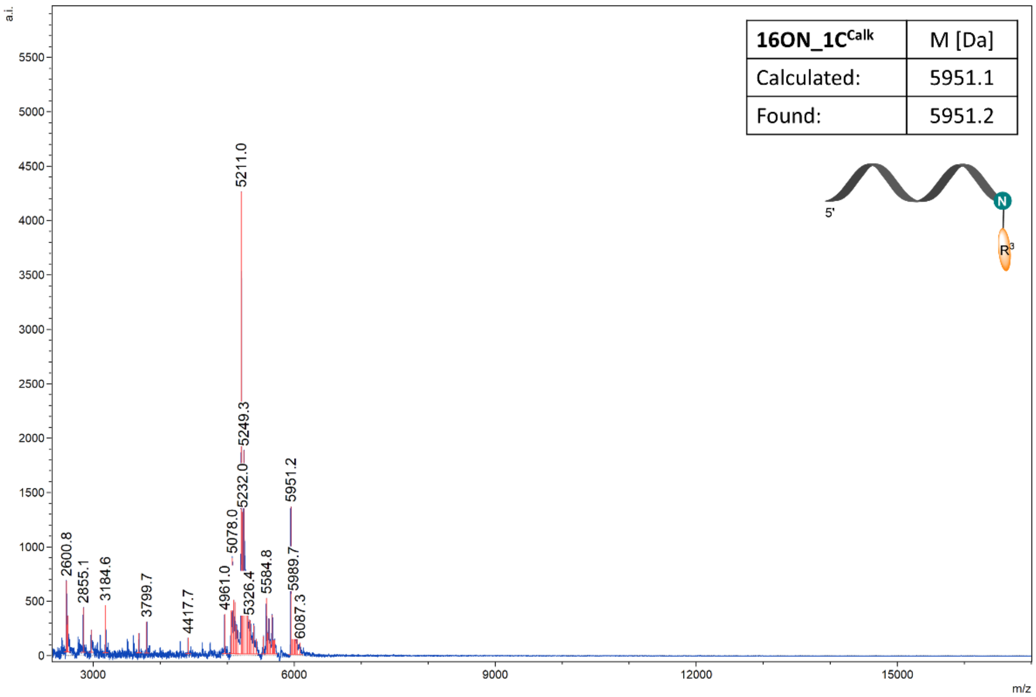

16ON_1C^{Calk}	M [Da]
Calculated:	5951.1
Found:	5951.2

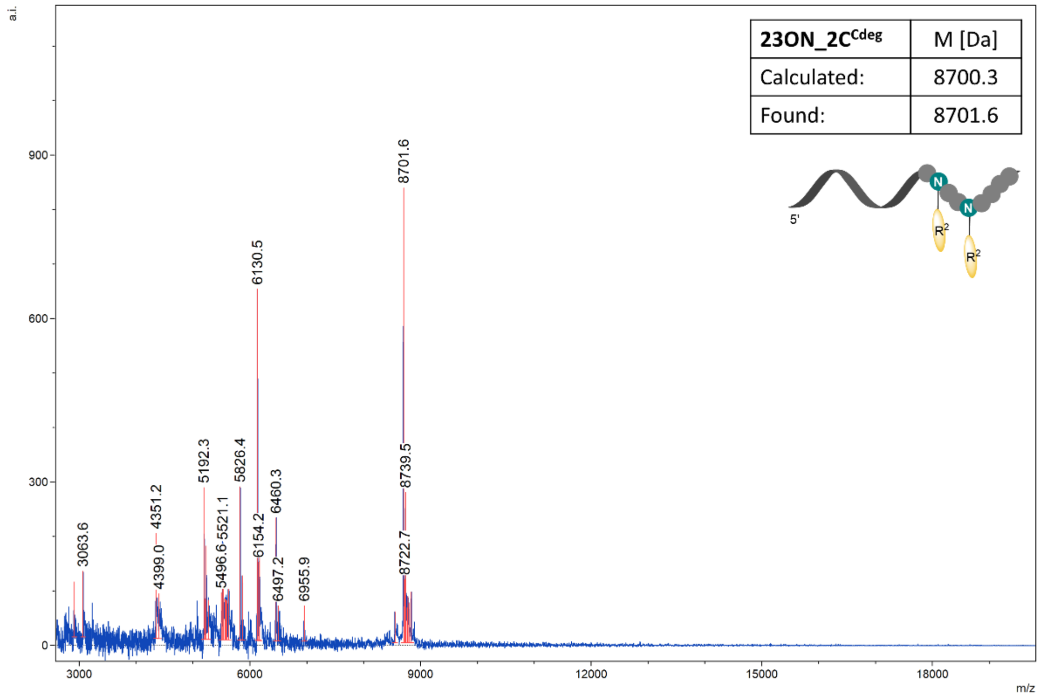

23ON_2C^Cdeg
M [Da]
Calculated:
8700.3
Found:
8701.6
5'
R2
R2
N
N
a.i.
m/z
8701.6
6130.5
5192.3
5826.4
6460.3
8739.5
4351.2
3063.6
5496.6
5521.1
6154.2
4399.0
6497.2
8722.7
6955.9

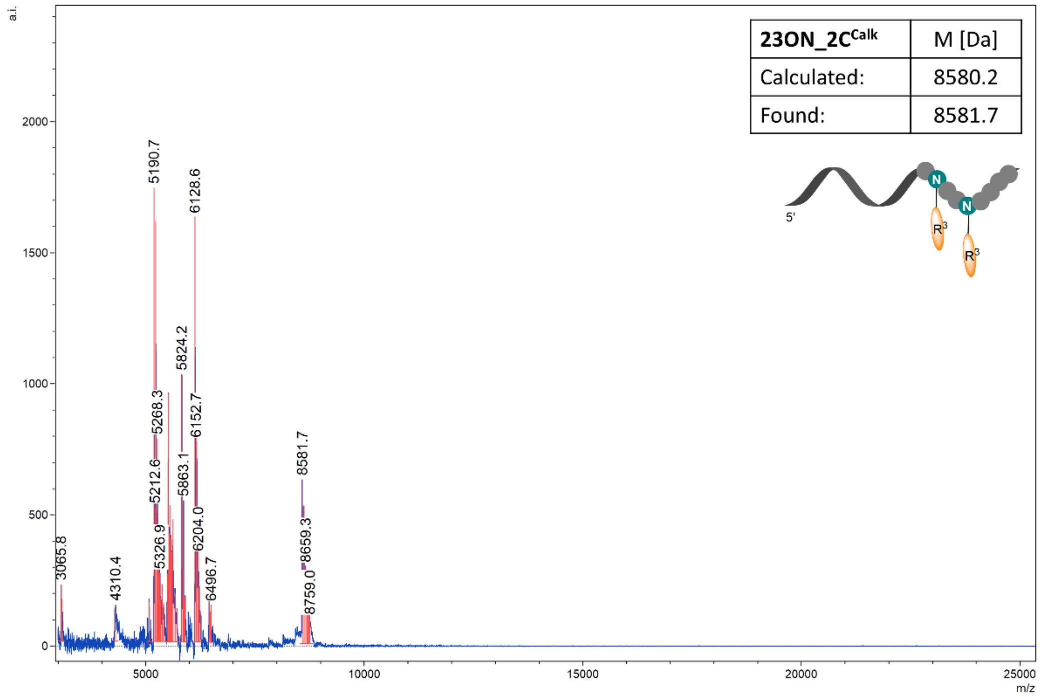

23ON_2C^Calk
M [Da]
Calculated:
8580.2
Found:
8581.7
5'
R3
R3
N
N
a.i.
m/z
5190.7
6128.6
5824.2
5268.3
6152.7
8581.7
5212.6
5863.1
6204.0
8659.3
3065.8
4310.4
5326.9
6496.7
8759.0

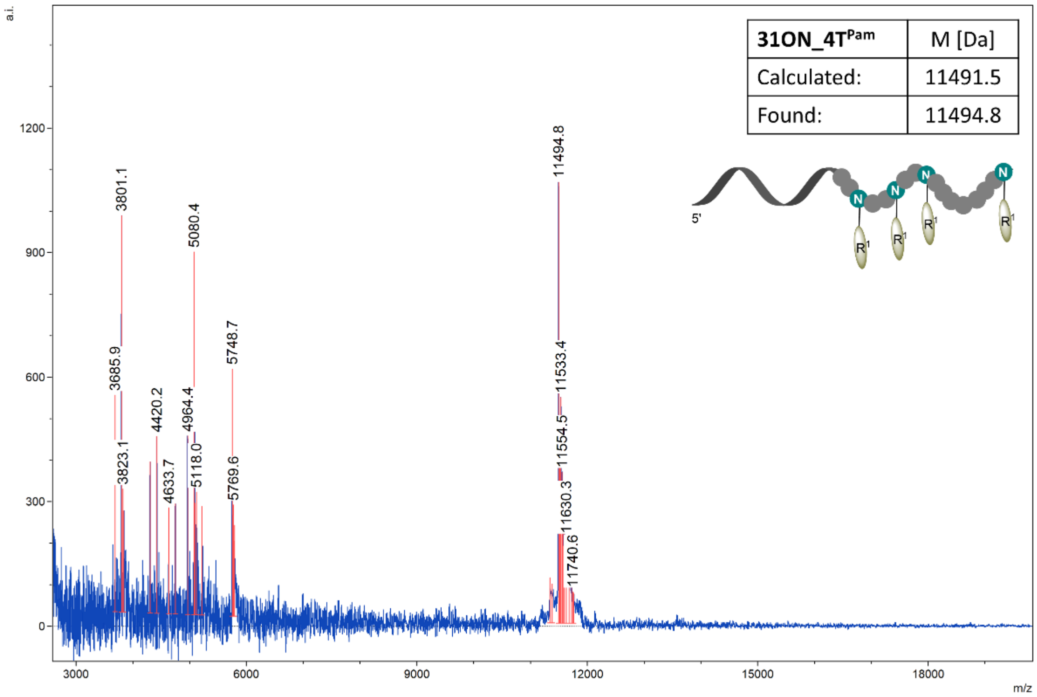
31ON_4T^Pam | M [Da]
Calculated: | 11491.5
Found: | 11494.8
a.i.
m/z
11494.8
3801.1
5080.4
5748.7
3685.9
4420.2
4964.4
11533.4
11554.5
3823.1
4633.7
5118.0
5769.6
11630.3
11740.6
5'
N
R1

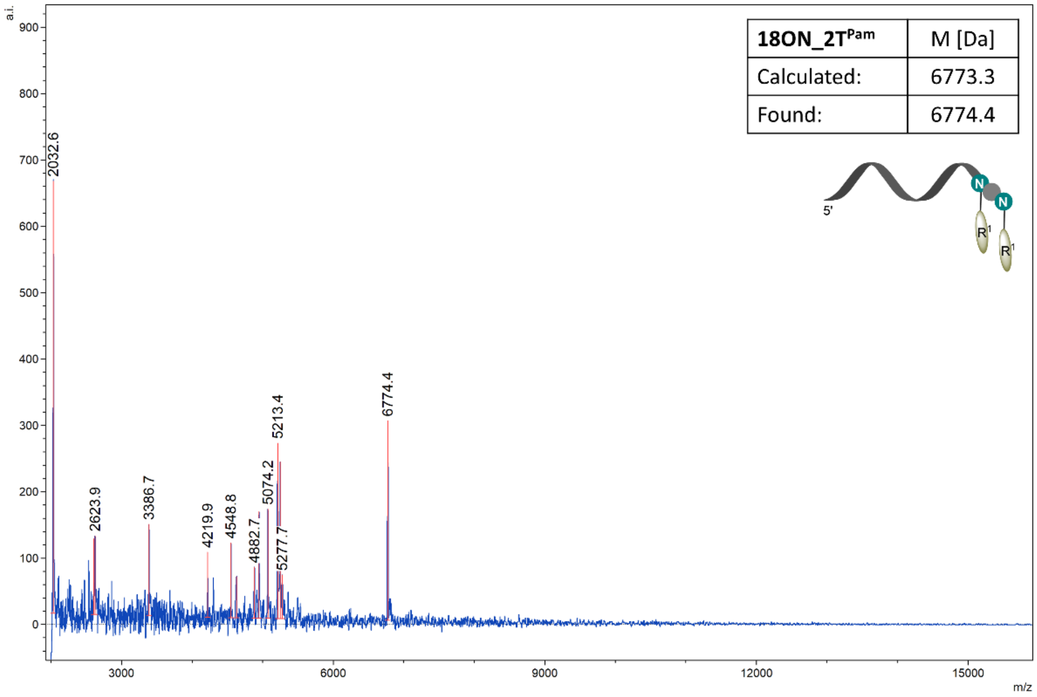
18ON_2T^Pam | M [Da]
Calculated: | 6773.3
Found: | 6774.4
a.i.
m/z
2032.6
6774.4
5213.4
5074.2
3386.7
2623.9
4219.9
4548.8
4882.7
5277.7
5'
N
R1

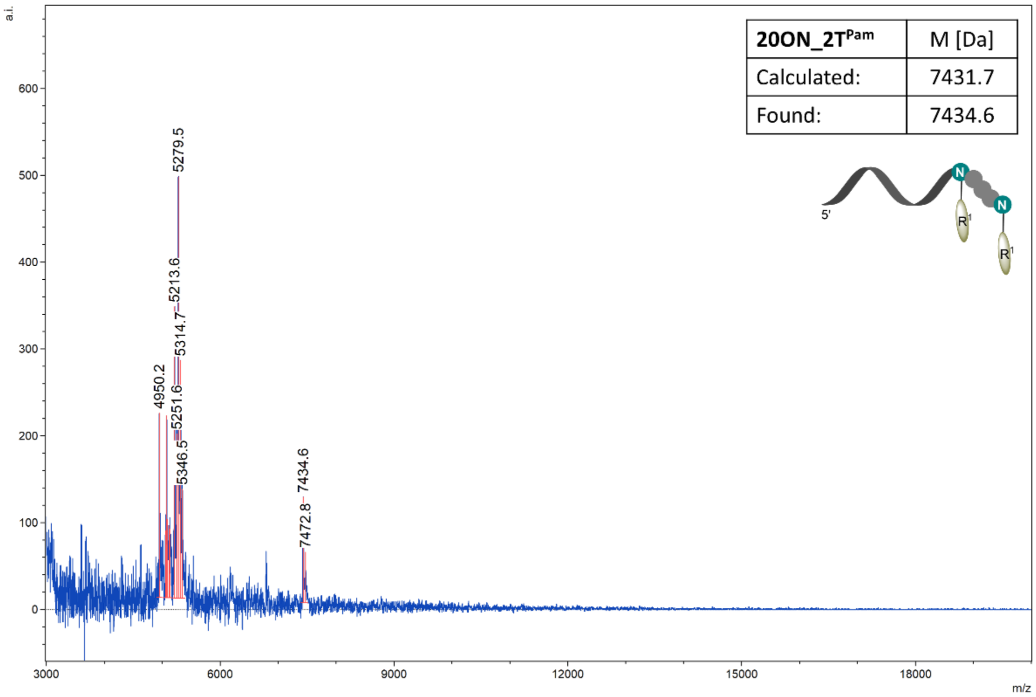

20ON_2T^Pam
M [Da]
Calculated:
7431.7
Found:
7434.6
5279.5
5213.6
5314.7
4950.2
5251.6
5346.5
7434.6
7472.8
5'
N
R1
a.i.
m/z

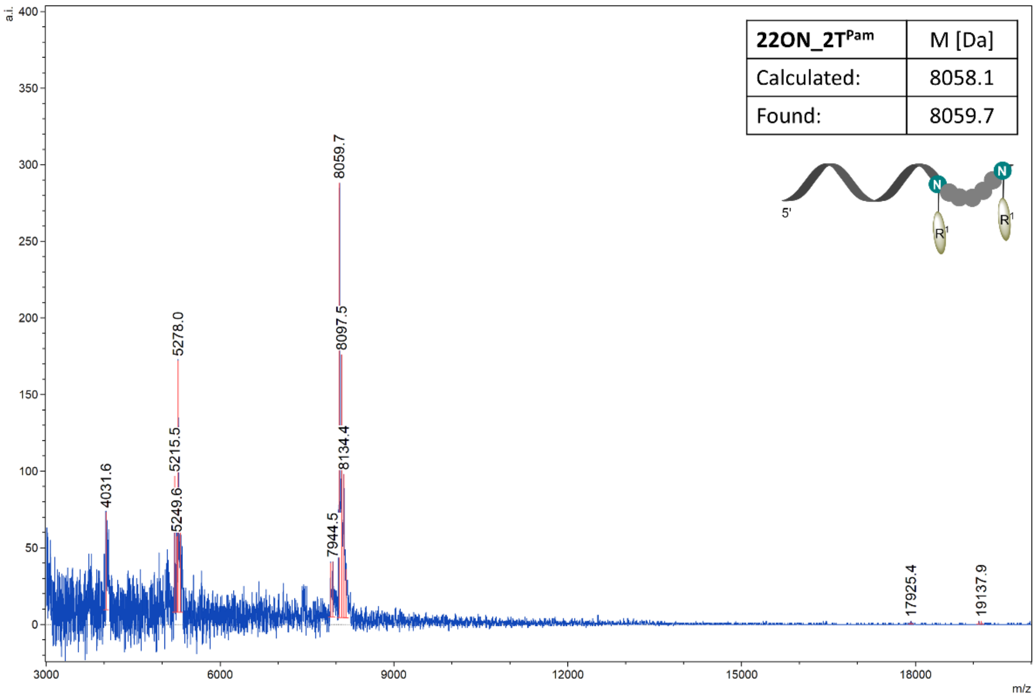

22ON_2T^Pam
M [Da]
Calculated:
8058.1
Found:
8059.7
8059.7
8097.5
8134.4
7944.5
5278.0
5215.5
5249.6
4031.6
17925.4
19137.9
5'
N
R1
a.i.
m/z

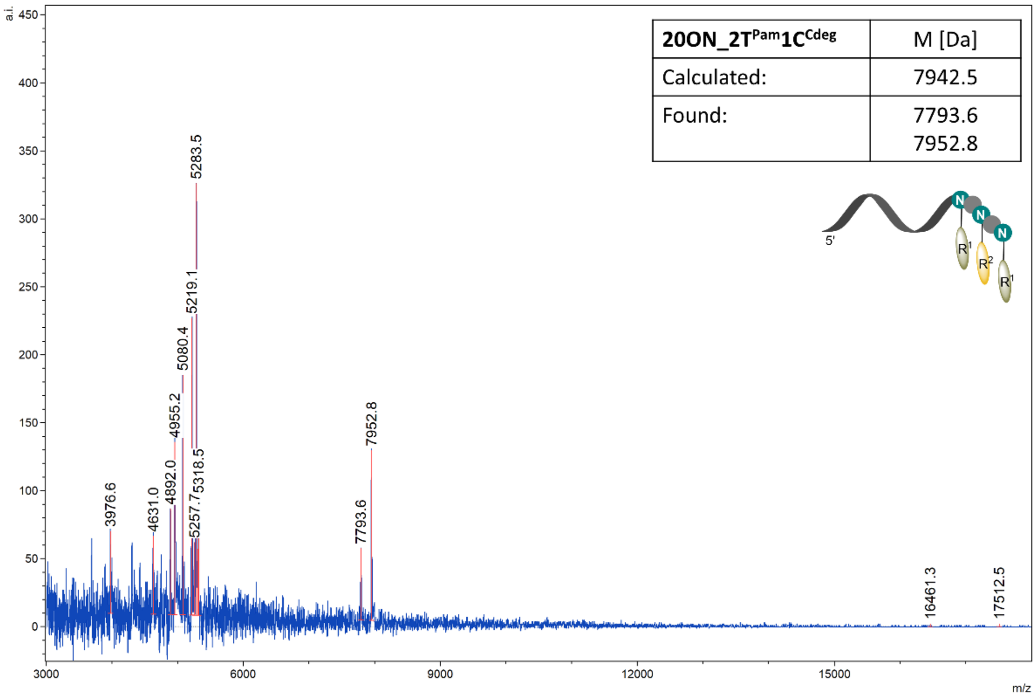
20ON_2T^{Pam}1C^{Cdeg} | M [Da]
Calculated: 7942.5
Found: 7793.6 7952.8
5'
a.i.
m/z
5283.5
5219.1
5080.4
4955.2
7952.8
3976.6
4631.0
4892.0
5257.7
5318.5
7793.6
16461.3
17512.5

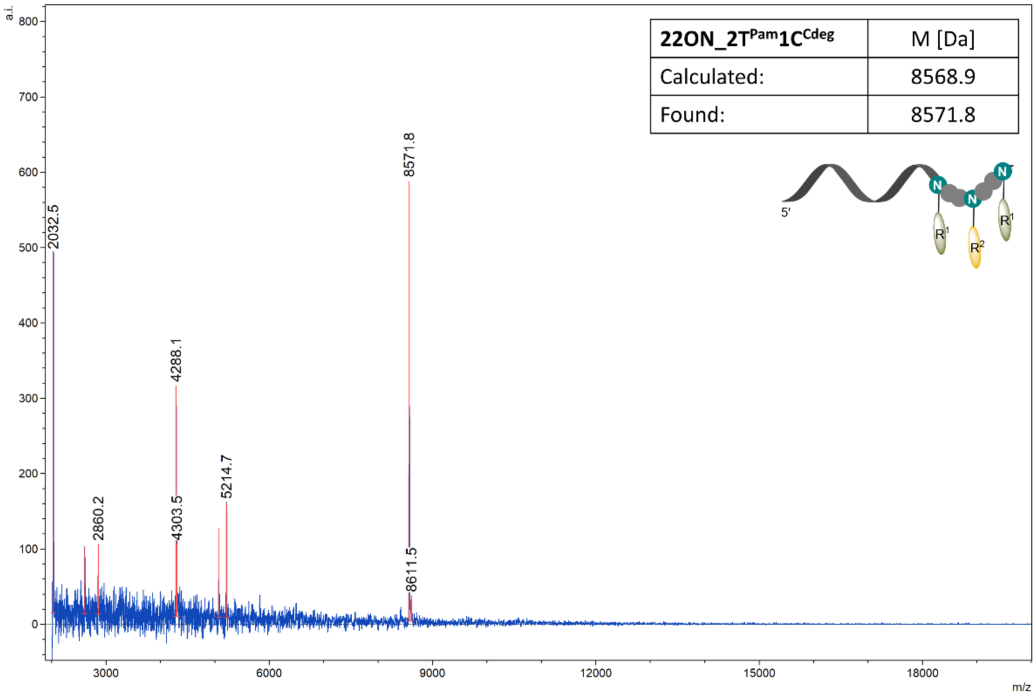
22ON_2T^{Pam}1C^{Cdeg} | M [Da]
Calculated: 8568.9
Found: 8571.8
5'
a.i.
m/z
8571.8
2032.5
4288.1
5214.7
2860.2
4303.5
8611.5

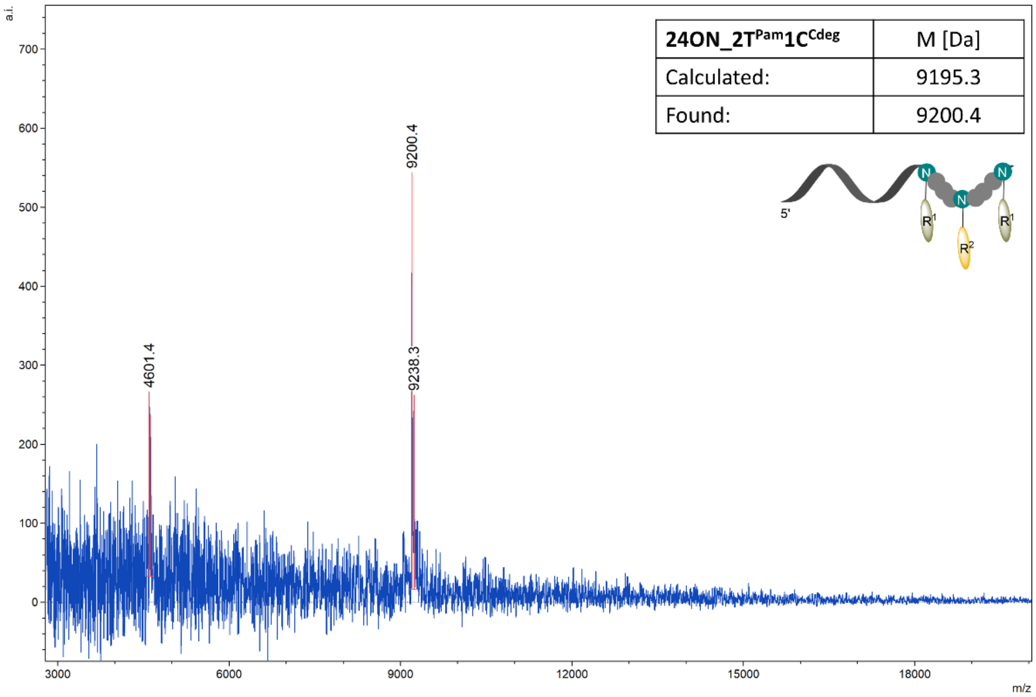
24ON_2T^Pam1C^Cdeg
M [Da]
Calculated: 9195.3
Found: 9200.4
5'
N
R1
R2
9200.4
9238.3
4601.4
a.i.
m/z

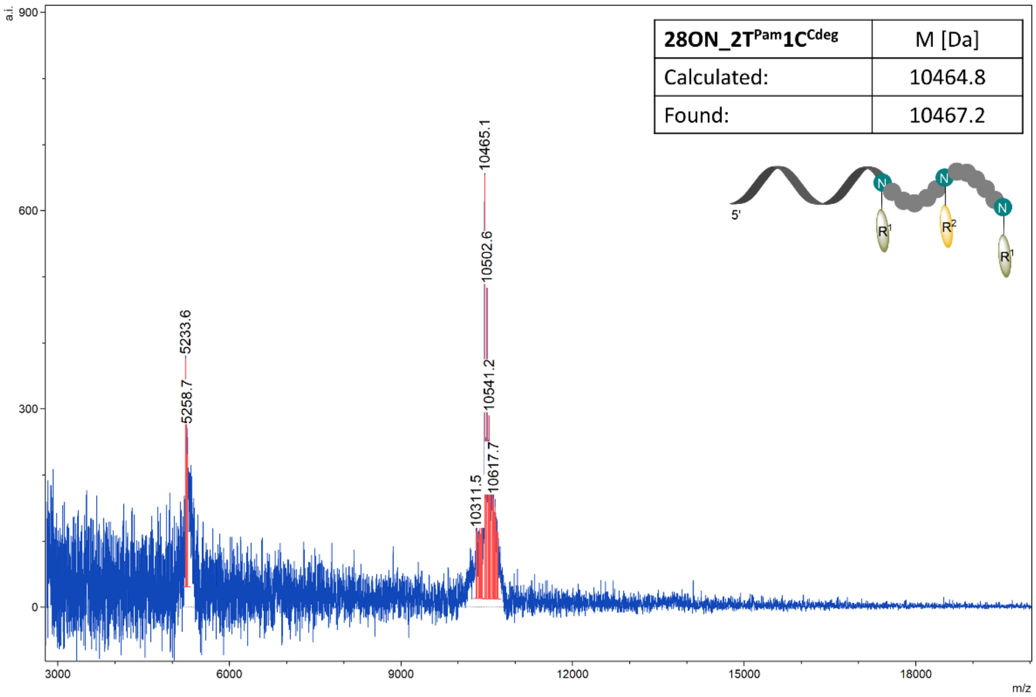
28ON_2T^Pam1C^Cdeg
M [Da]
Calculated: 10464.8
Found: 10467.2
5'
N
R1
R2
10465.1
10502.6
10541.2
10617.7
10311.5
5233.6
5258.7
a.i.
m/z

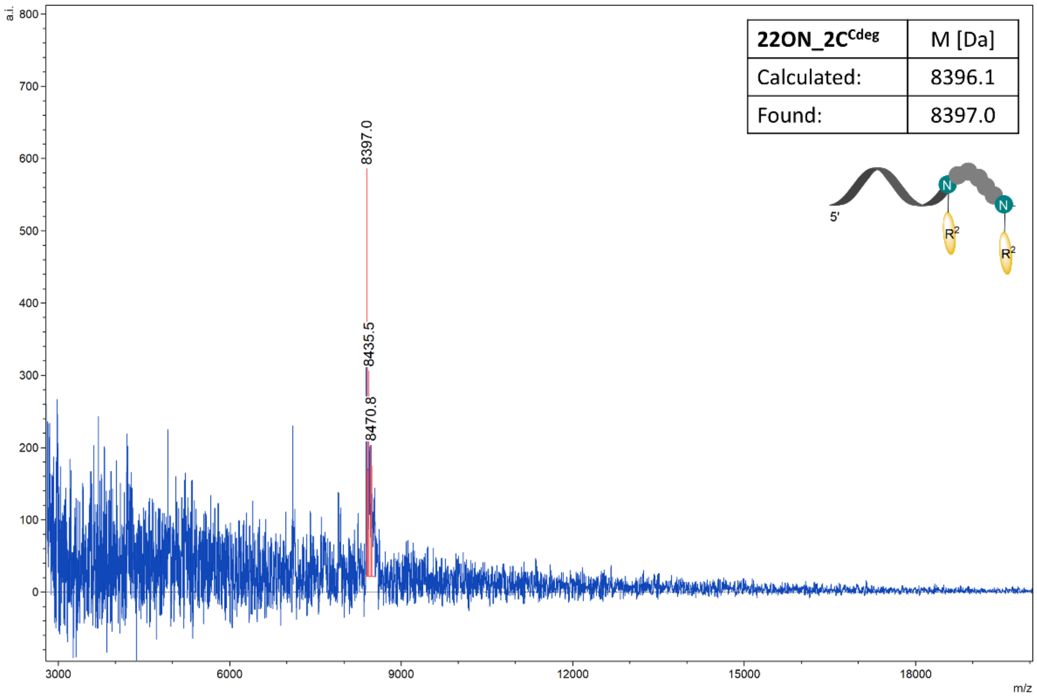
a.i.
800
700
600
500
400
300
200
100
0
3000
6000
9000
12000
15000
18000
m/z
8397.0
8435.5
8470.8
22ON_2CCdeg
M [Da]
Calculated:
8396.1
Found:
8397.0
5'
N
N
R2
R2

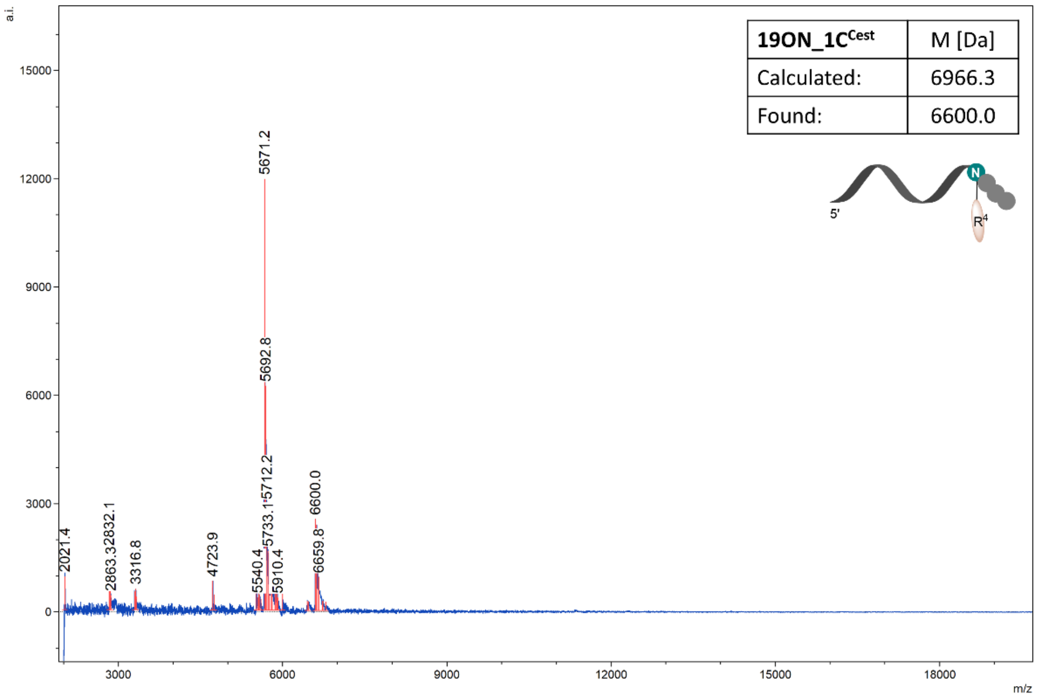
a.i.
15000
12000
9000
6000
3000
0
3000
6000
9000
12000
15000
18000
m/z
5671.2
5692.8
5733.1
5712.2
6600.0
6659.8
5910.4
5540.4
4723.9
3316.8
2863.3
2832.1
2021.4
19ON_1CCest
M [Da]
Calculated:
6966.3
Found:
6600.0
5'
N
R4

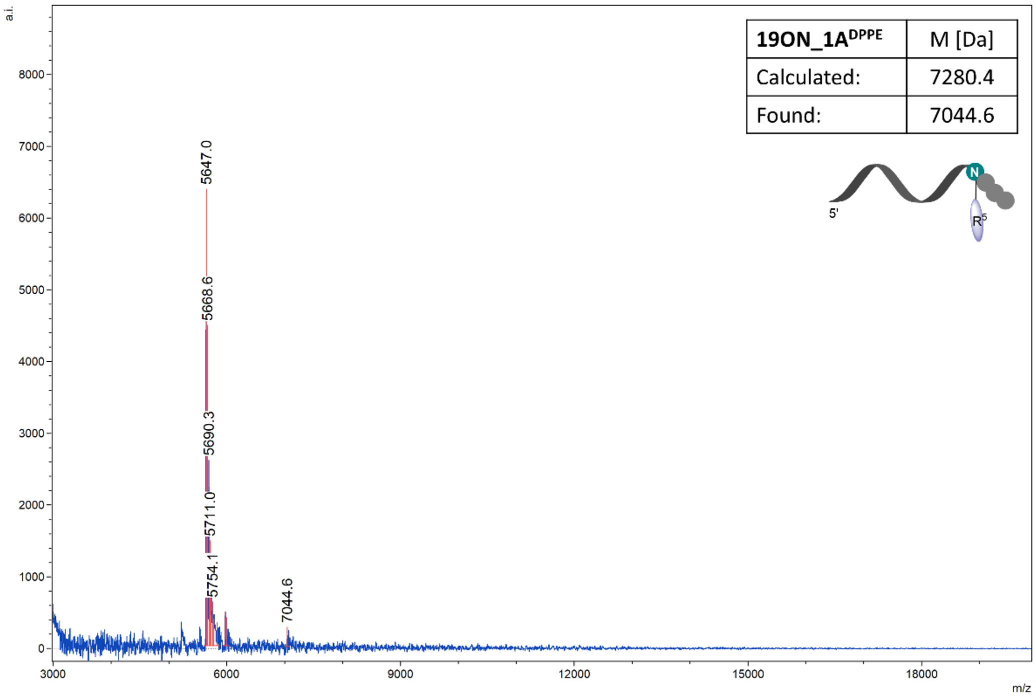
a.i.
19ON_1A^DPPE
M [Da]
Calculated:
7280.4
Found:
7044.6
5'
N
R^5
5647.0
5668.6
5690.3
5711.0
5754.1
7044.6
m/z

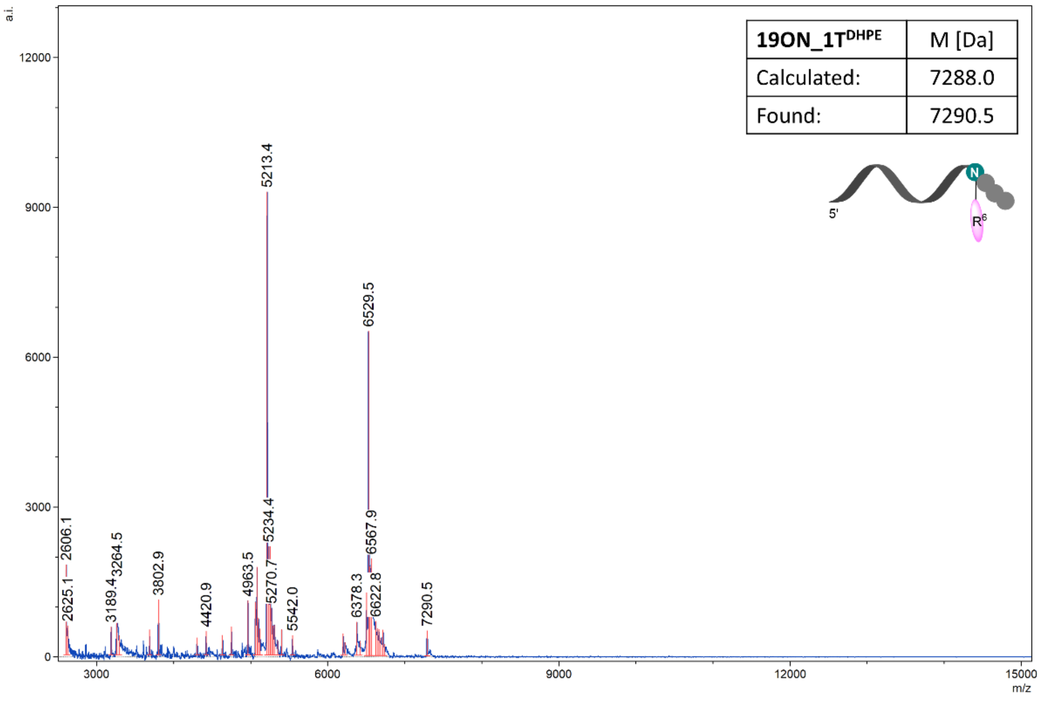
a.i.
19ON_1T^DHPE
M [Da]
Calculated:
7288.0
Found:
7290.5
5'
N
R^6
5213.4
6529.5
2606.1
2625.1
3189.4
3264.5
3802.9
4420.9
4963.5
5234.4
5270.7
5542.0
6378.3
6567.9
6622.8
7290.5
m/z

4. Copies of NMR spectra

^{1}H, ^{13}C, NMR spectra of compound 2, dT^{Pam}:

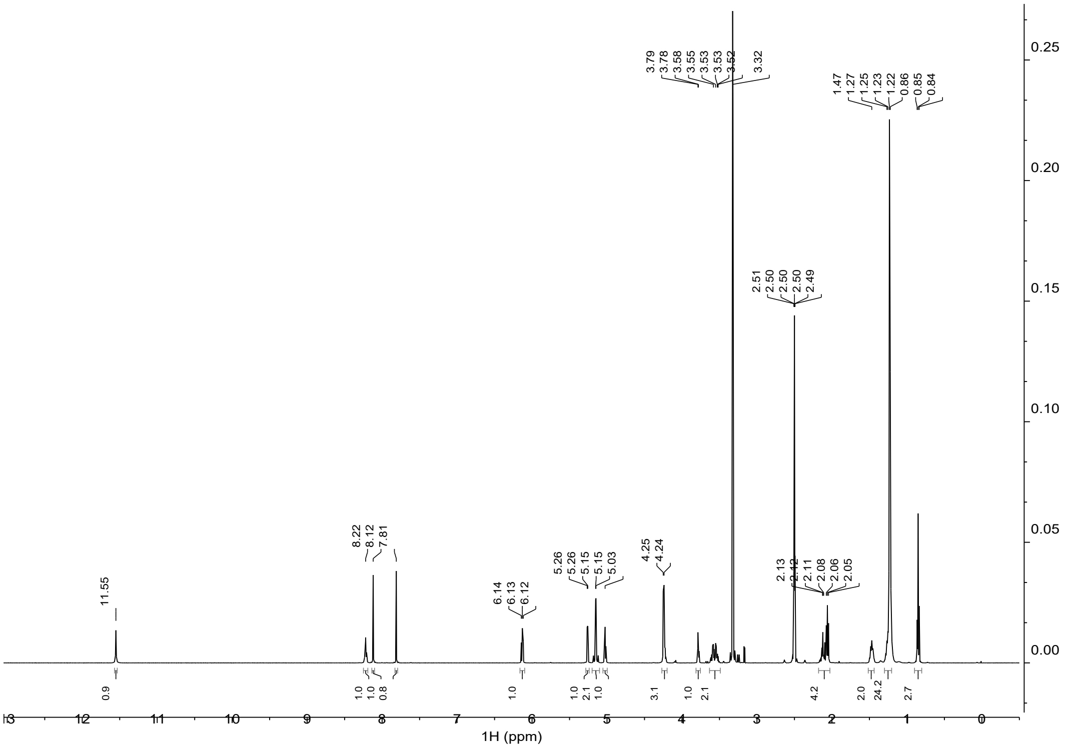

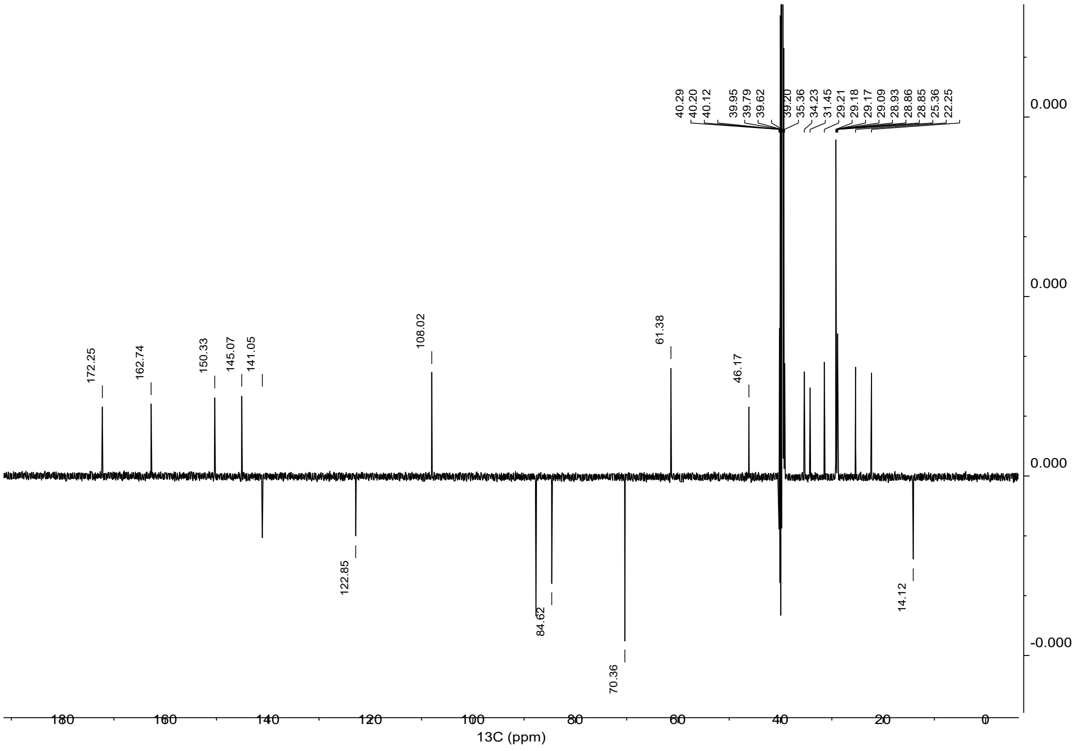

^{1}H, ^{13}C, ^{31}P, NMR spectra of compound 3, dT[Pam]TP:

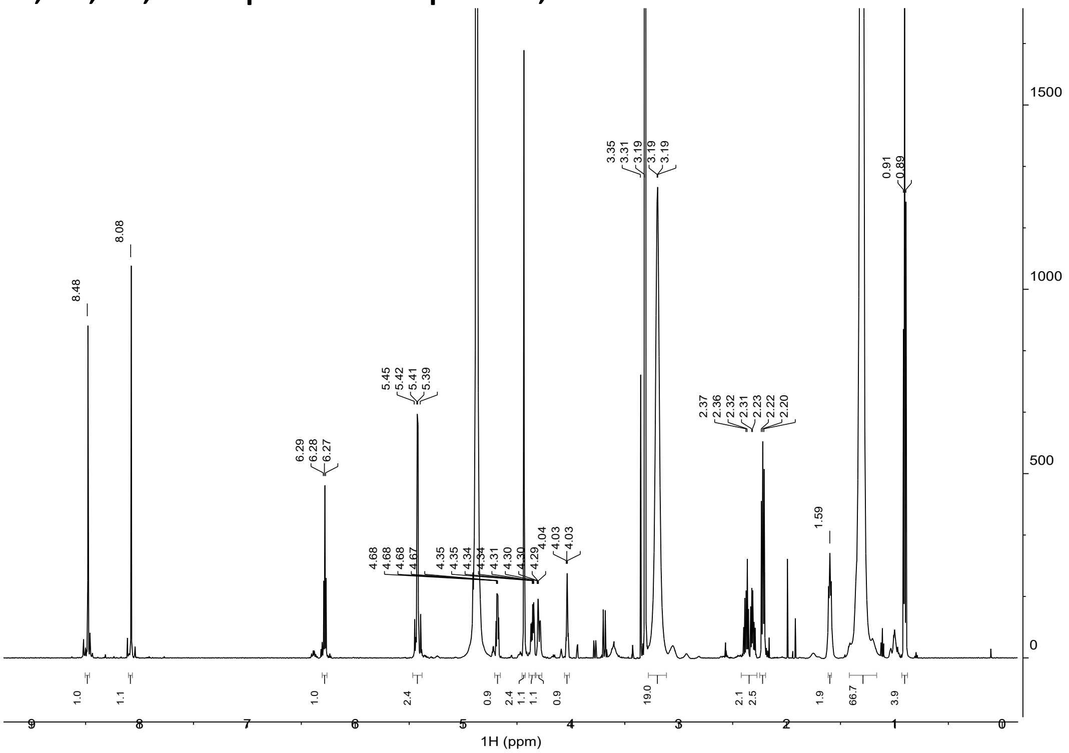

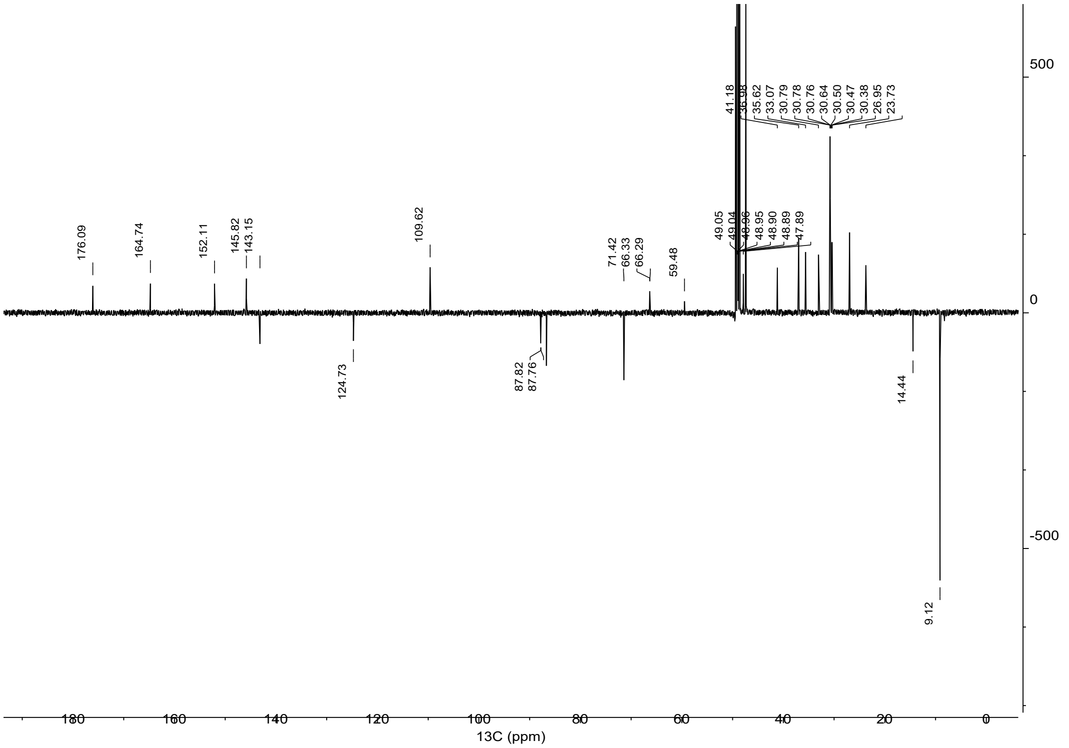

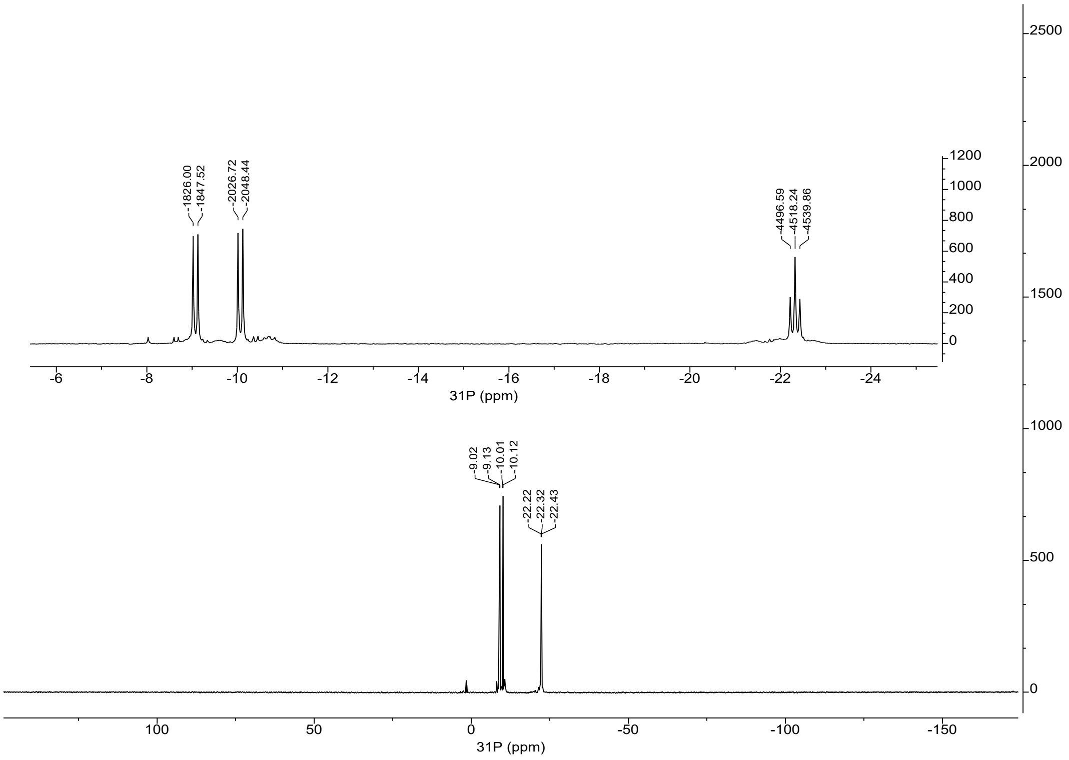

^{1}H, ^{13}C, NMR spectra compound 5, dC^{Cest}:

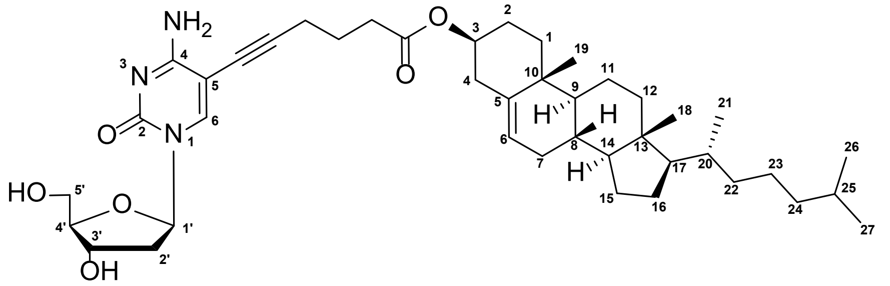

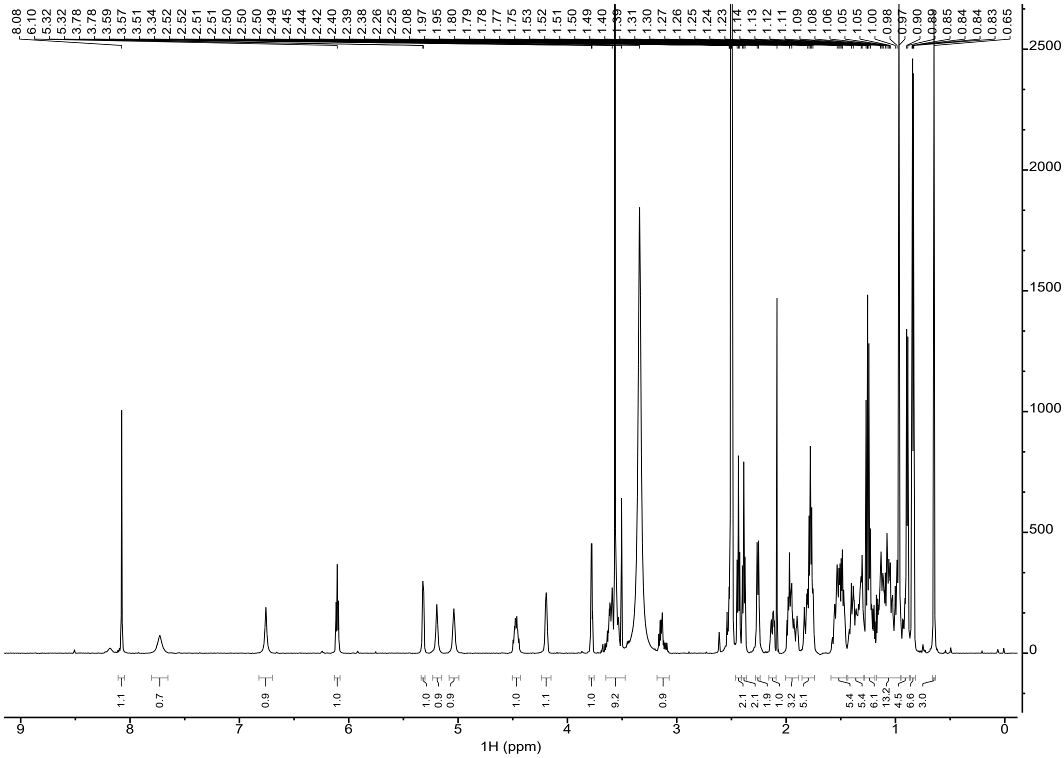
8.08
6.10
5.32
5.32
3.78
3.78
3.59
3.57
3.51
3.34
2.52
2.52
2.51
2.51
2.50
2.50
2.49
2.45
2.44
2.42
2.40
2.39
2.38
2.26
2.25
2.08
1.97
1.95
1.80
1.79
1.78
1.77
1.75
1.53
1.52
1.51
1.50
1.49
1.40
1.39
1.31
1.30
1.27
1.26
1.25
1.24
1.23
1.14
1.13
1.12
1.11
1.09
1.08
1.06
1.05
1.05
1.00
0.98
0.97
0.90
0.89
0.85
0.84
0.84
0.83
0.65
1H (ppm)

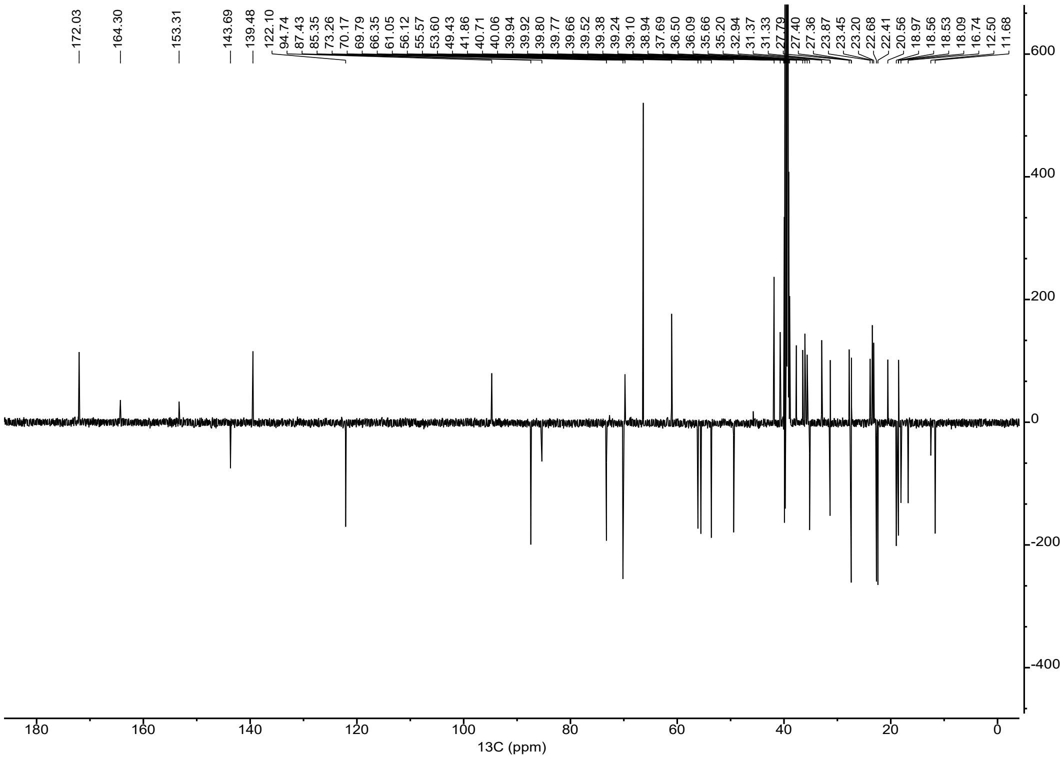
172.03
164.30
153.31
143.69
139.48
122.10
94.74
87.43
85.35
73.26
70.17
69.79
66.35
61.05
56.12
55.57
53.60
49.43
41.86
40.71
40.06
39.94
39.92
39.80
39.77
39.66
39.52
39.38
39.24
39.10
38.94
37.69
36.50
36.09
35.66
35.20
32.94
31.37
31.33
27.79
27.40
27.36
23.87
23.45
23.20
22.68
22.41
20.56
18.97
18.56
18.53
18.09
16.74
12.50
11.68
13C (ppm)

^{1}H, ^{13}C, ^{31}P, NMR spectra of compound 6, dC^{Cest}TP:

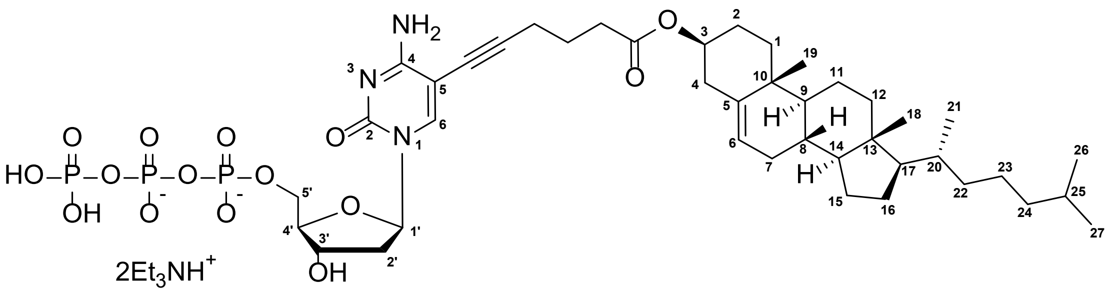

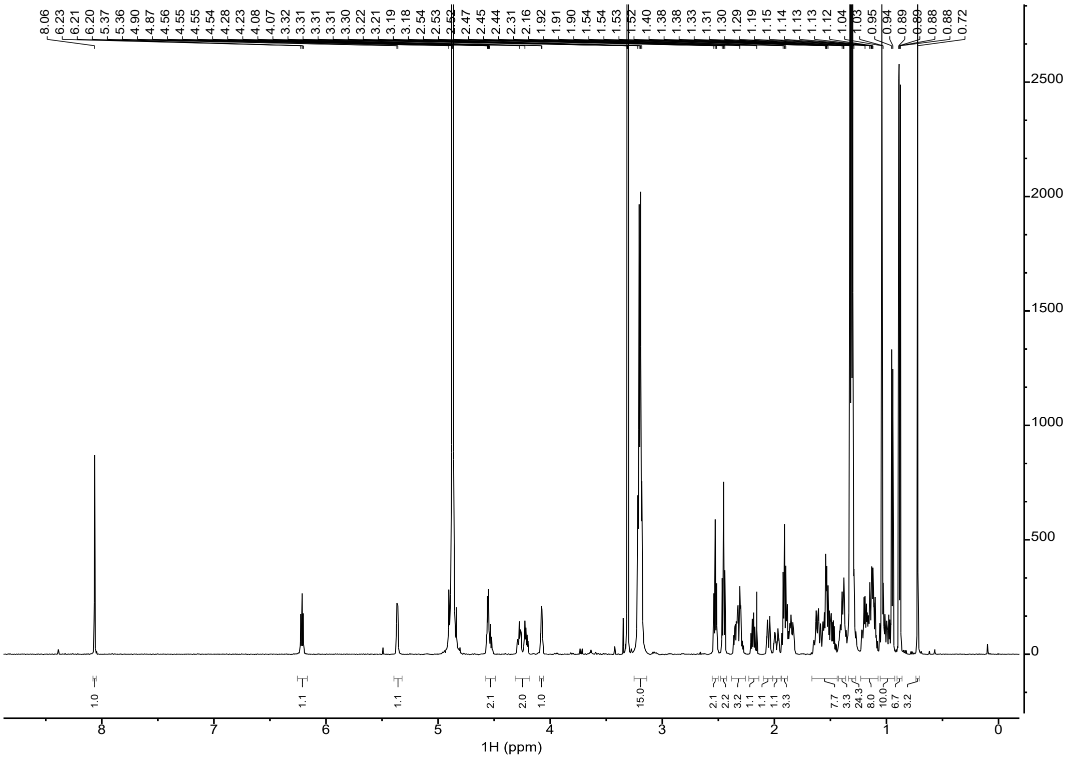

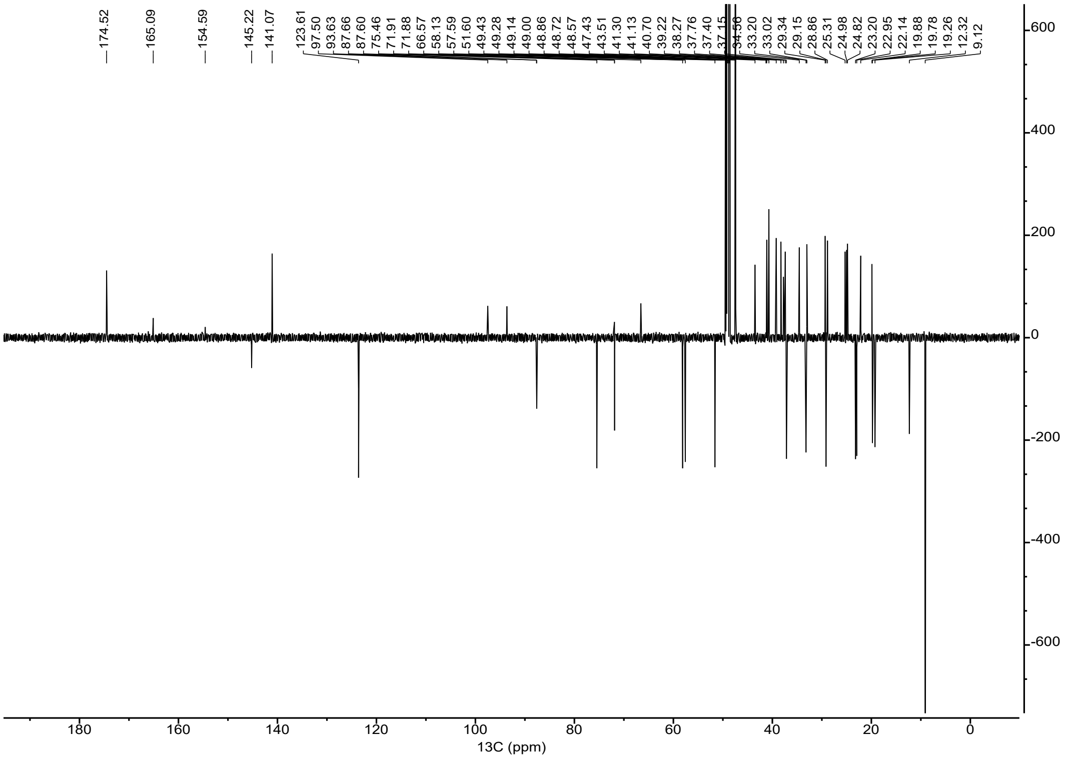
174.52
165.09
154.59
145.22
141.07
123.61
97.50
93.63
87.66
87.60
75.46
71.91
71.88
66.57
58.13
57.59
51.60
49.43
49.28
49.14
49.00
48.86
48.72
48.57
47.43
43.51
41.30
41.13
40.70
39.22
38.27
37.76
37.40
37.15
34.50
33.20
33.02
29.34
29.15
28.86
25.31
24.98
24.82
23.20
22.95
22.14
19.88
19.78
19.26
12.32
9.12
13C (ppm)

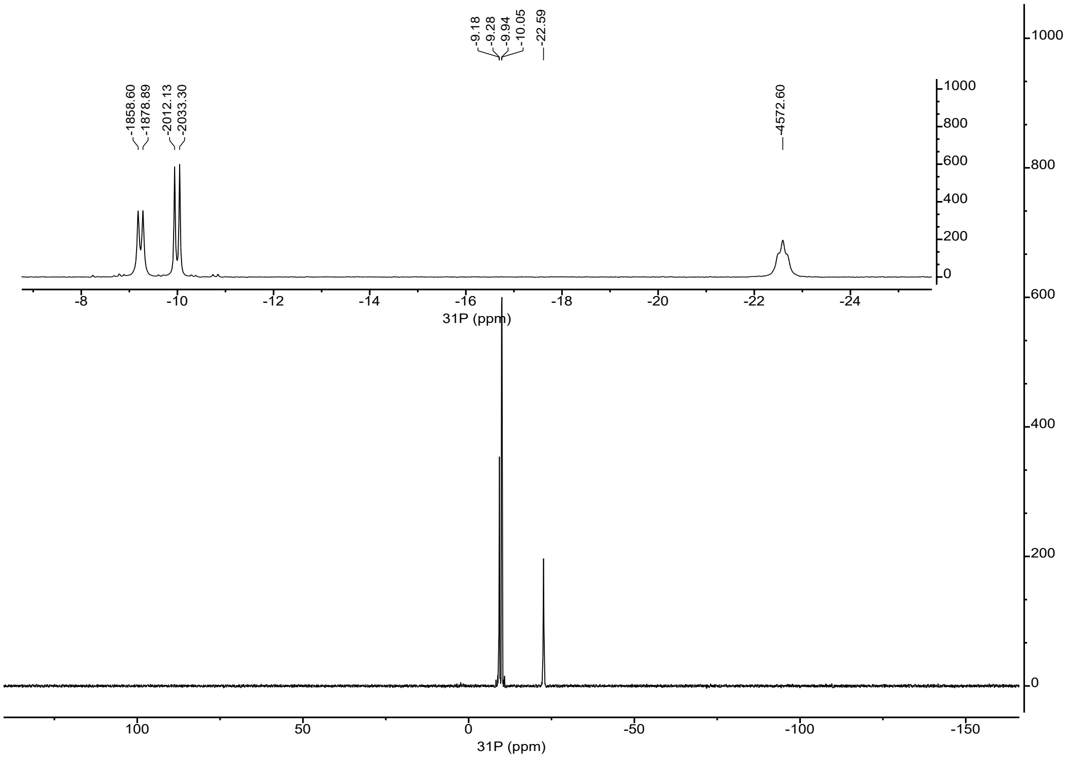
-9.18
-9.28
-9.94
-10.05
-22.59
-1858.60
-1878.89
-2012.13
-2033.30
-4572.60
31P (ppm)
31P (ppm)

^{1}H, ^{13}C, NMR spectra of compound 7:

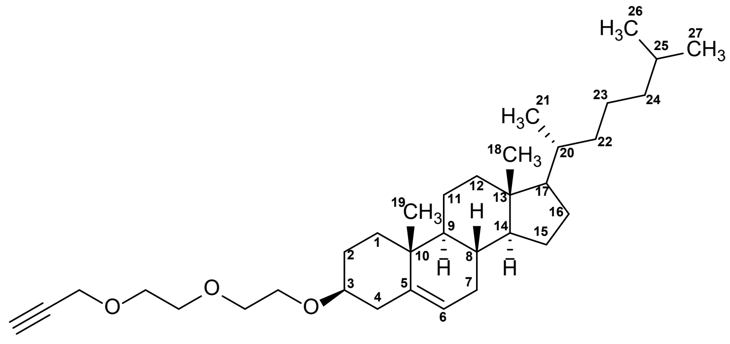

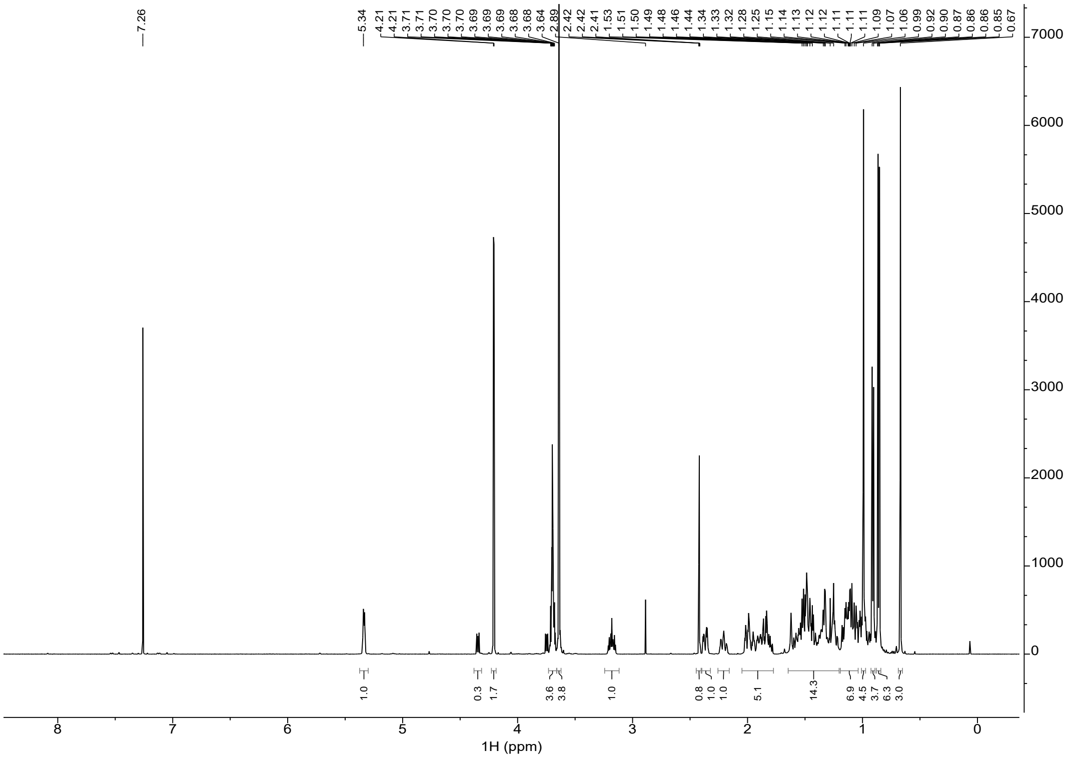

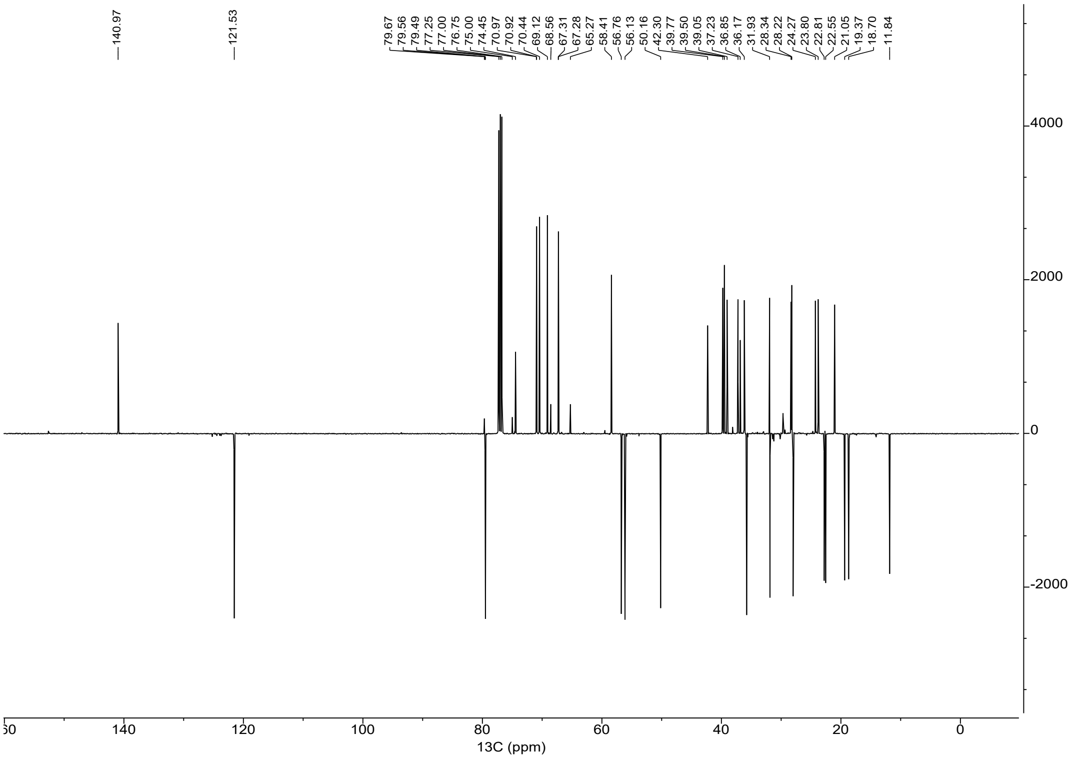

^{1}H, ^{13}C, NMR spectra of compound 8, dC^{Cdeg}:

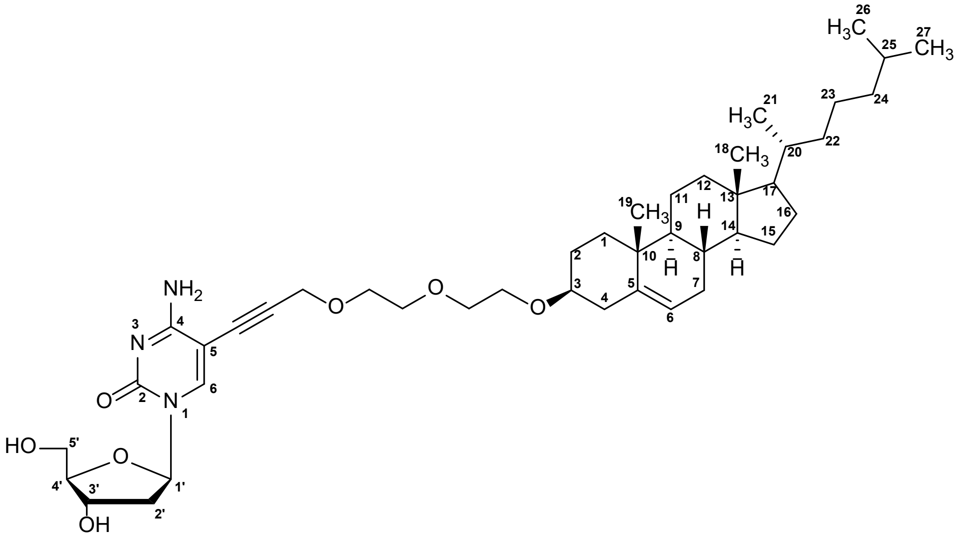

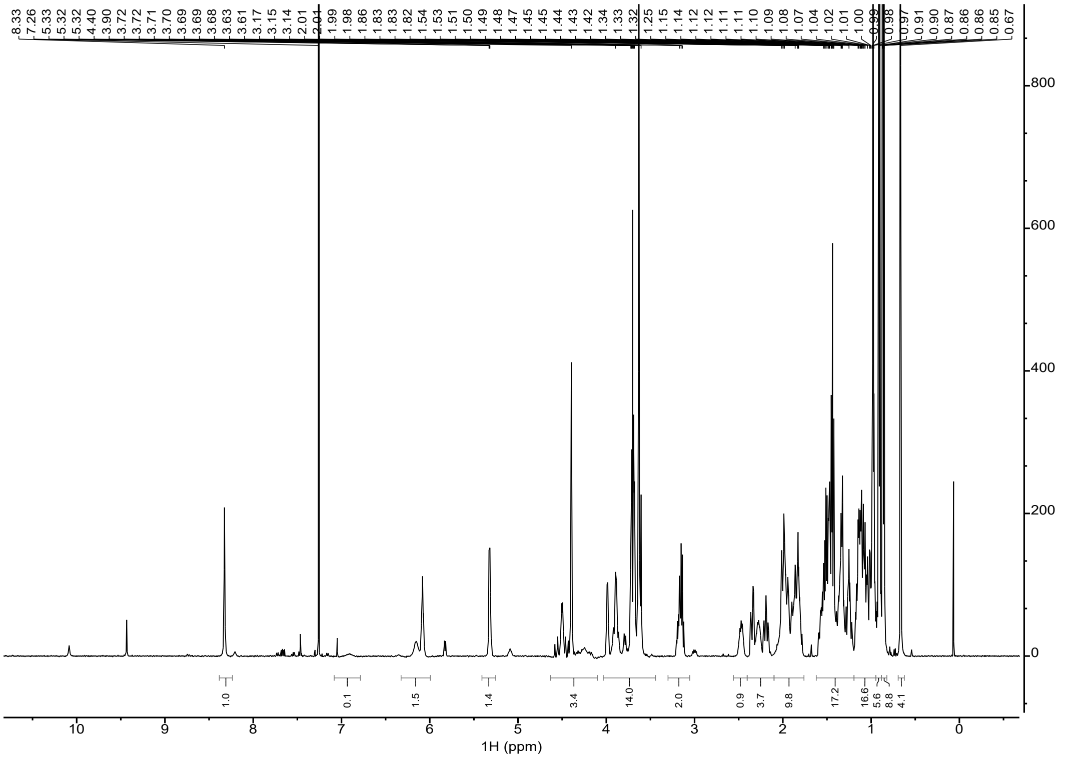
8.33
7.26
5.33
5.32
5.32
4.40
3.90
3.72
3.72
3.71
3.70
3.69
3.69
3.68
3.63
3.61
3.17
3.15
3.14
2.01
2.01
1.99
1.98
1.86
1.83
1.83
1.82
1.54
1.53
1.51
1.50
1.49
1.48
1.47
1.45
1.45
1.44
1.43
1.42
1.34
1.33
1.32
1.25
1.15
1.14
1.12
1.12
1.11
1.11
1.10
1.09
1.08
1.07
1.04
1.02
1.01
1.00
0.98
0.97
0.91
0.90
0.87
0.86
0.85
0.67
1.0
0.1
1.5
1.4
3.4
14.0
2.0
0.9
3.7
9.8
17.2
16.6
5.6
8.8
4.1
1H (ppm)

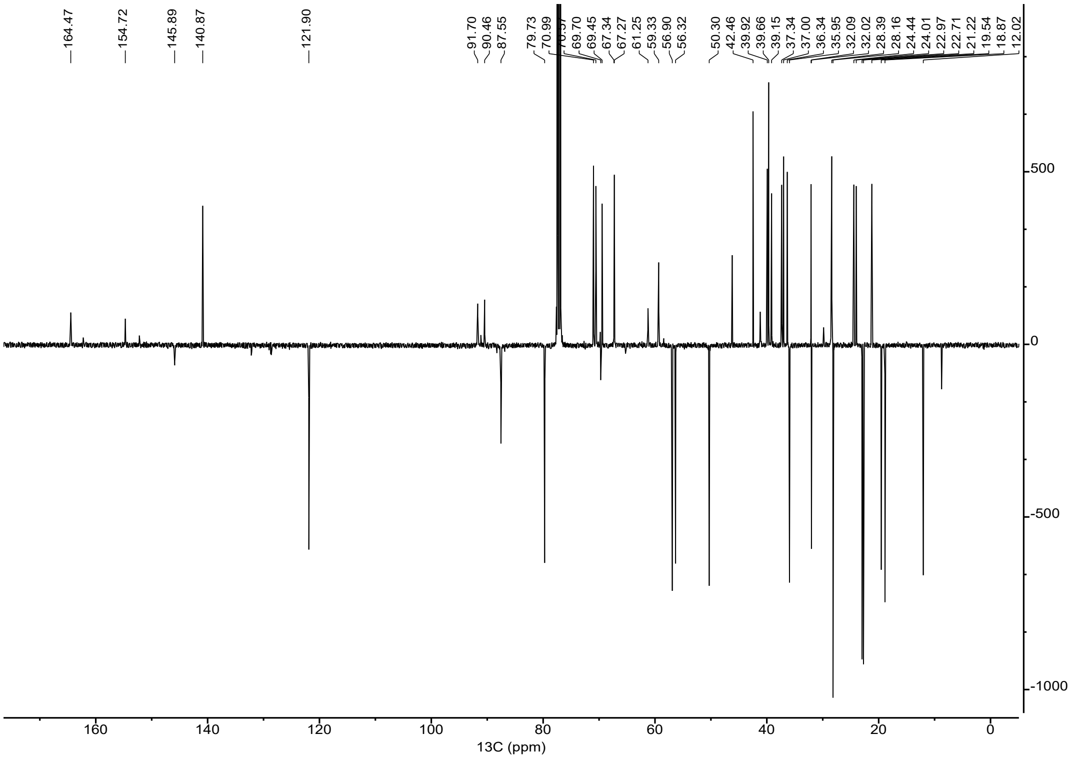
164.47
154.72
145.89
140.87
121.90
91.70
90.46
87.55
79.73
70.99
69.70
69.45
67.34
67.27
61.25
59.33
56.90
56.32
50.30
42.46
39.92
39.66
39.15
37.34
37.00
36.34
35.95
32.09
32.02
28.39
28.16
24.44
24.01
22.97
22.71
21.22
19.54
18.87
12.02
13C (ppm)

^{1}H, ^{13}C, ^{31}P, NMR spectra of compound 9, dC^{Cdeg}TP:

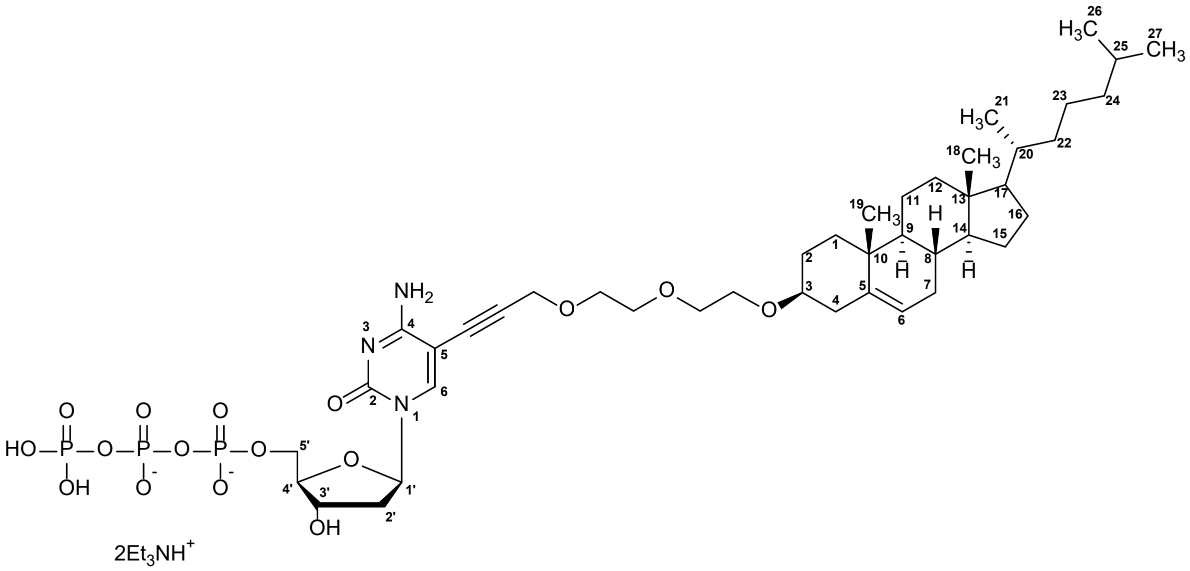

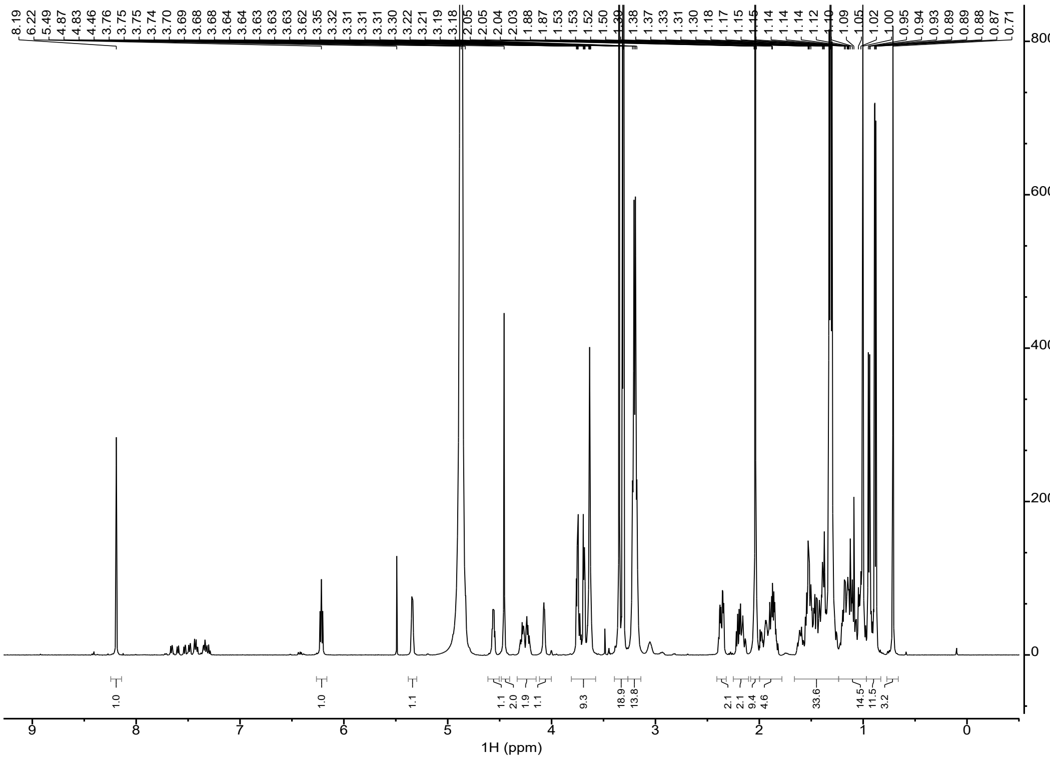

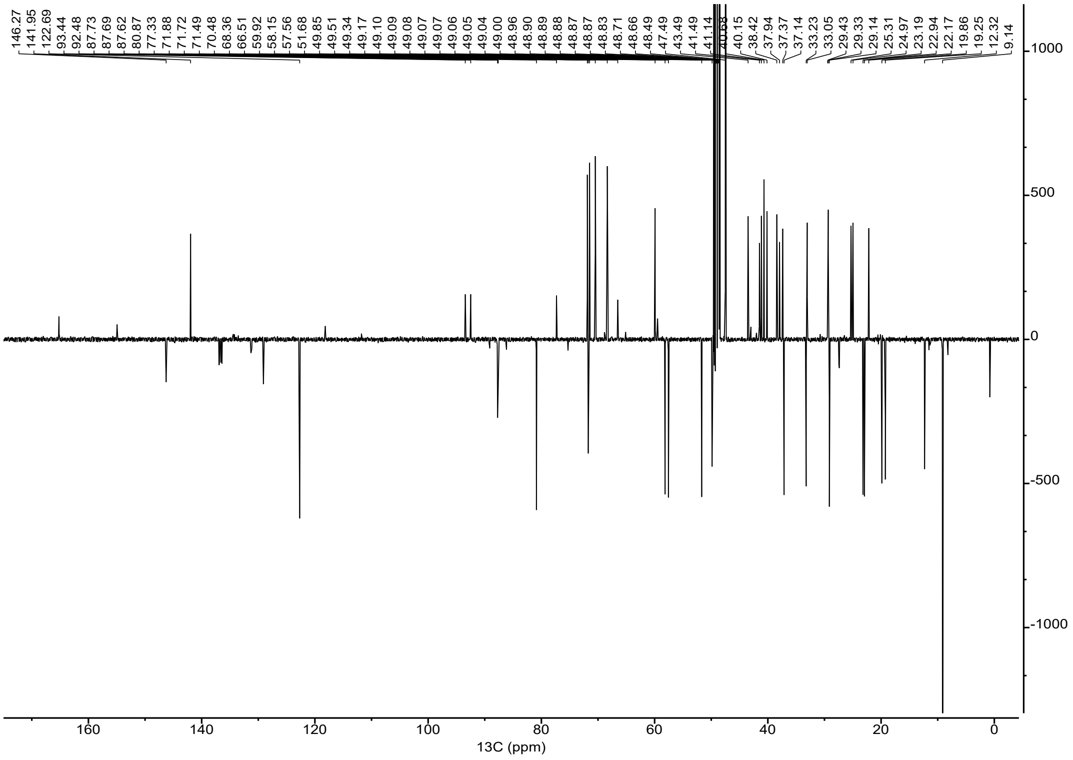
13C (ppm)

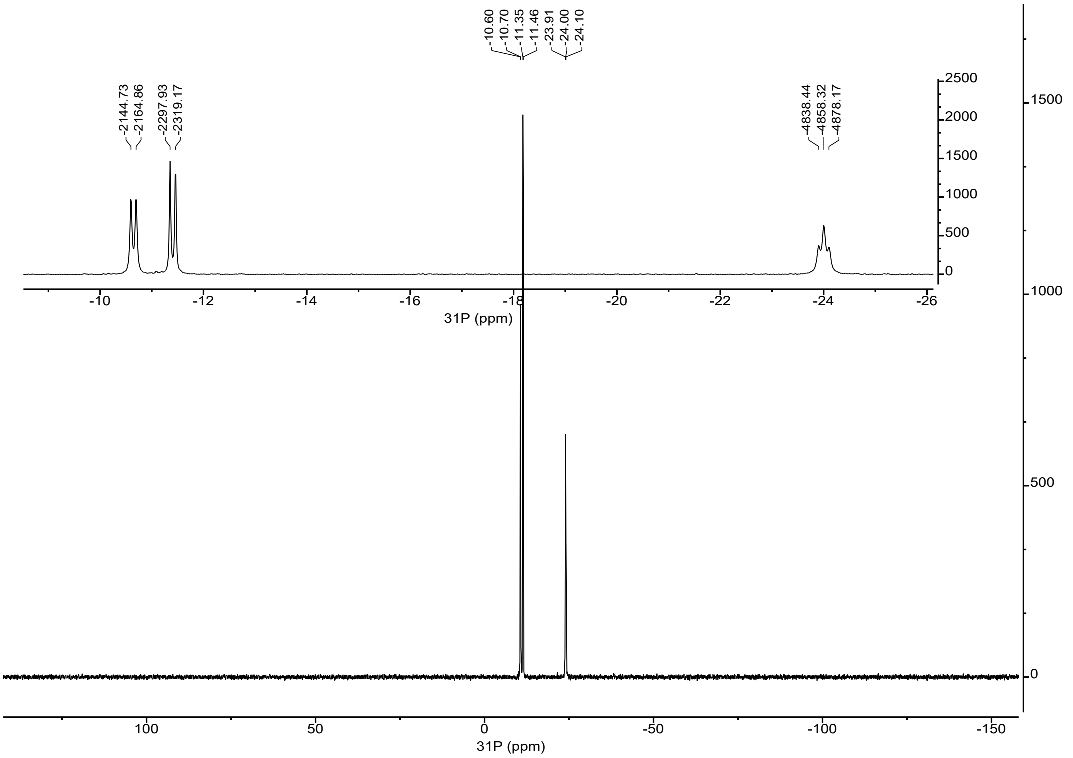
31P (ppm)
31P (ppm)

^{1}H, ^{13}C NMR spectra of compound 11, dC[Calk]:

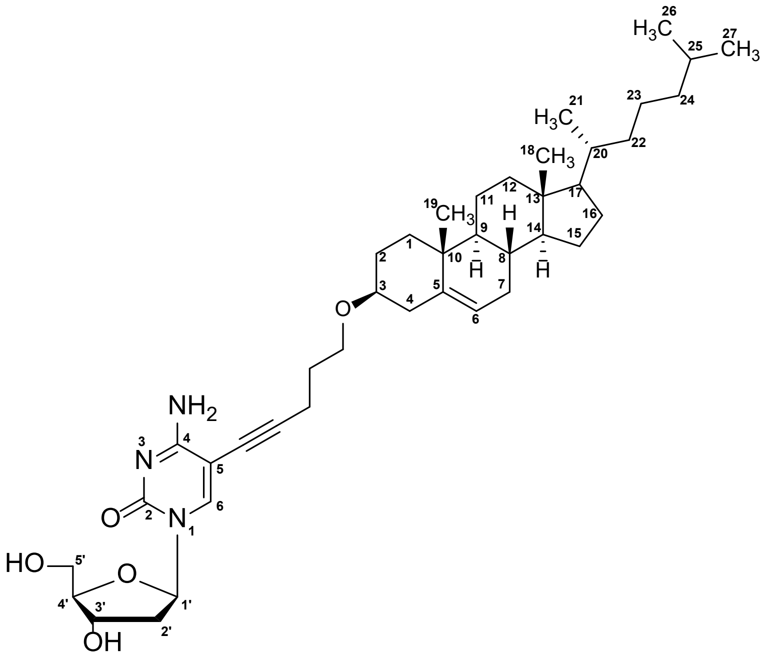

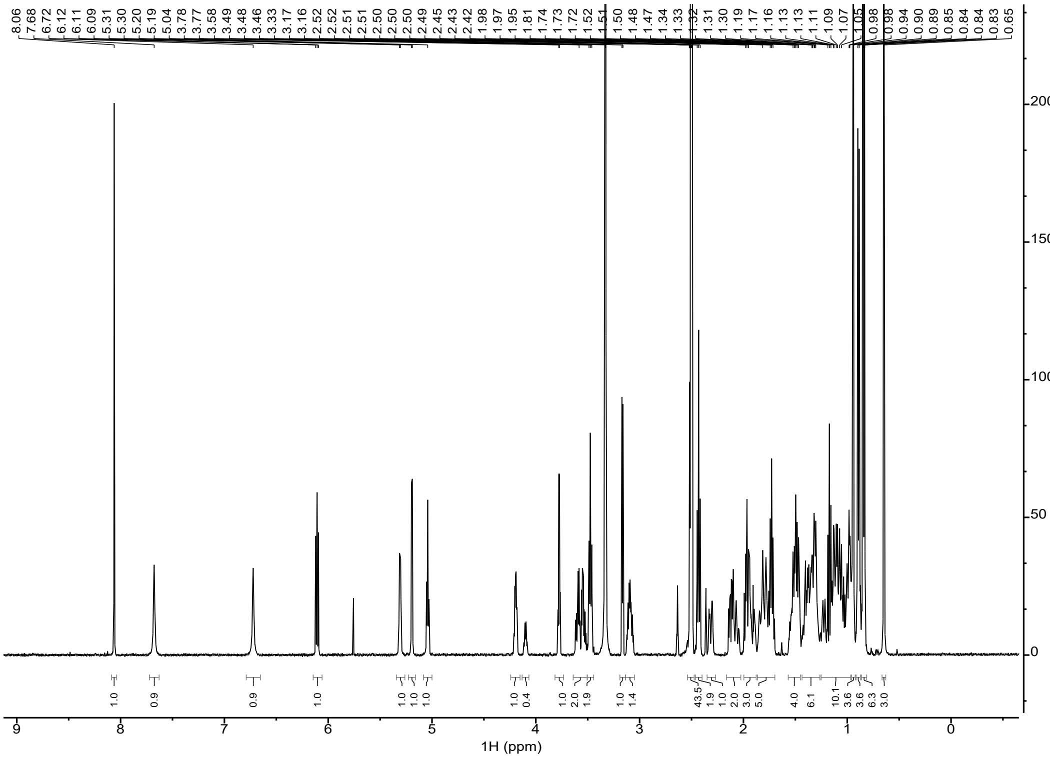

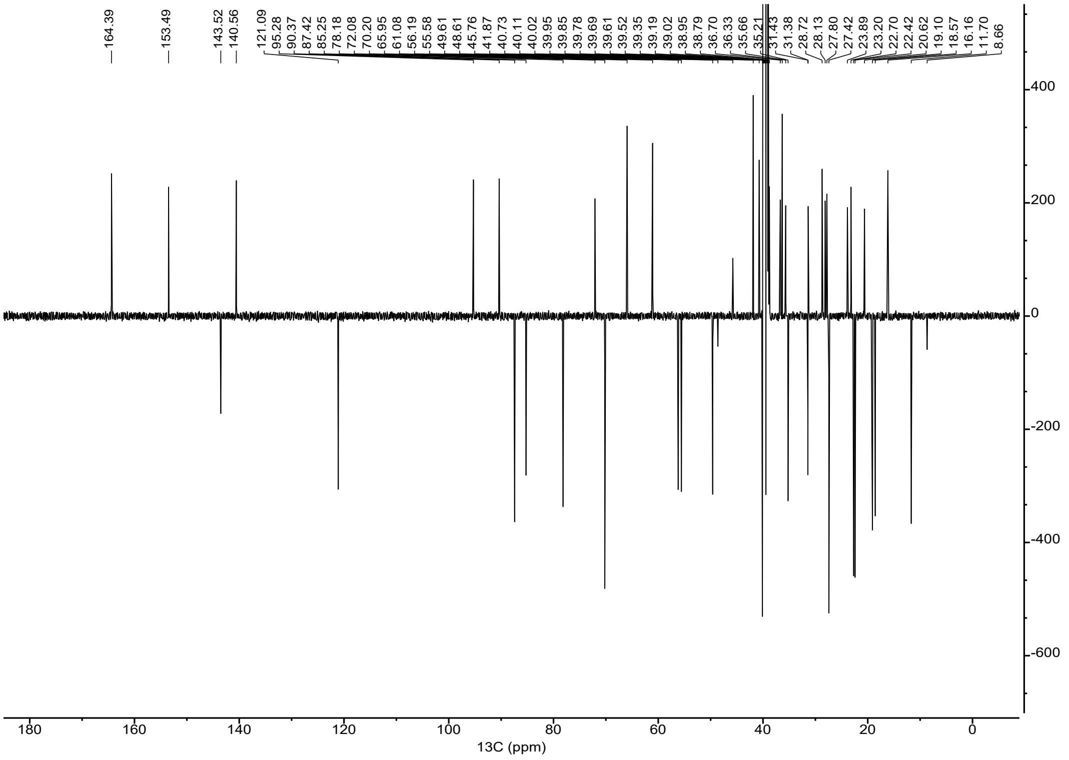

^{1}H, ^{13}C, ^{31}P, NMR spectra of compound 12, dC^{Calk}TP:

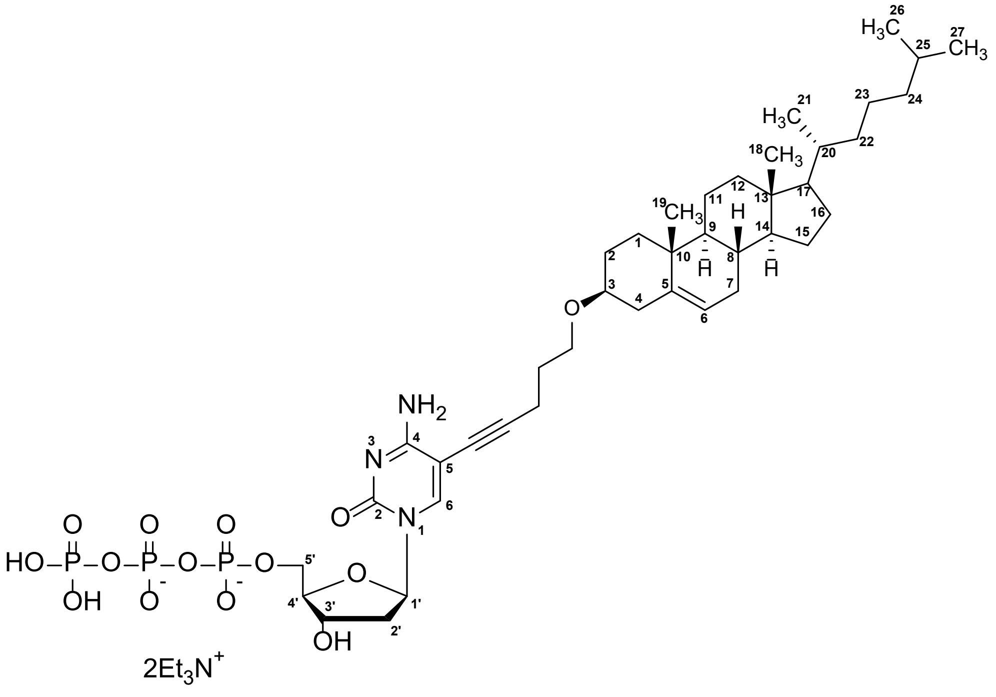

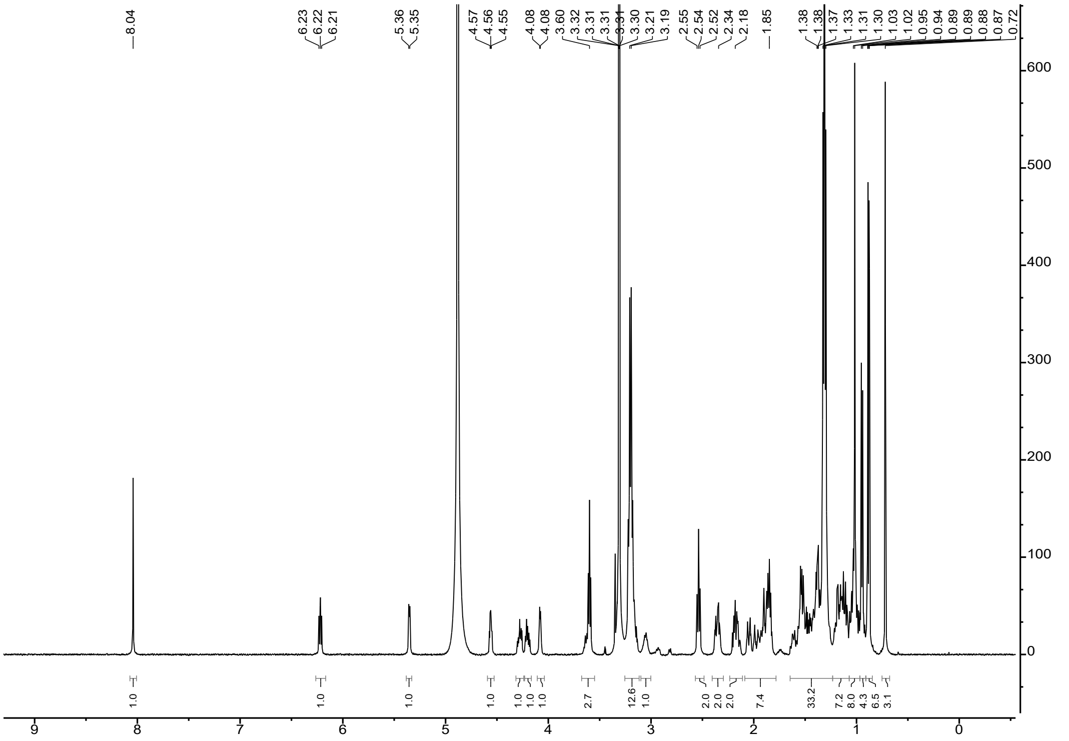
1H (ppm)

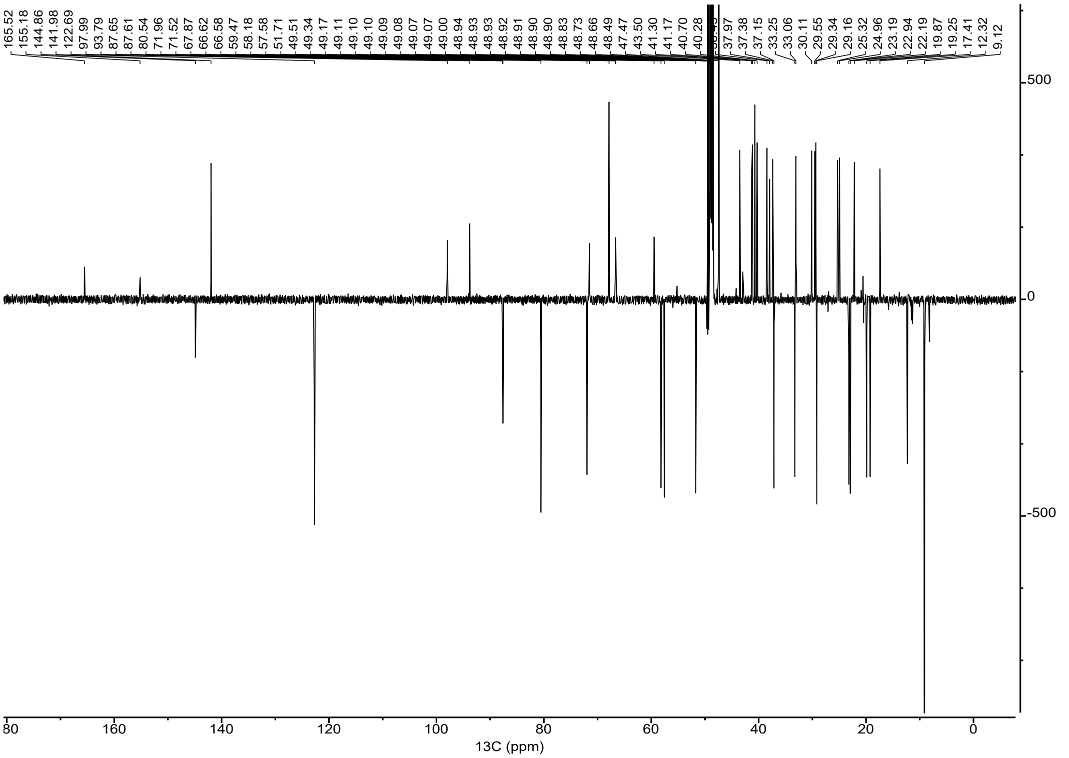
13C (ppm)

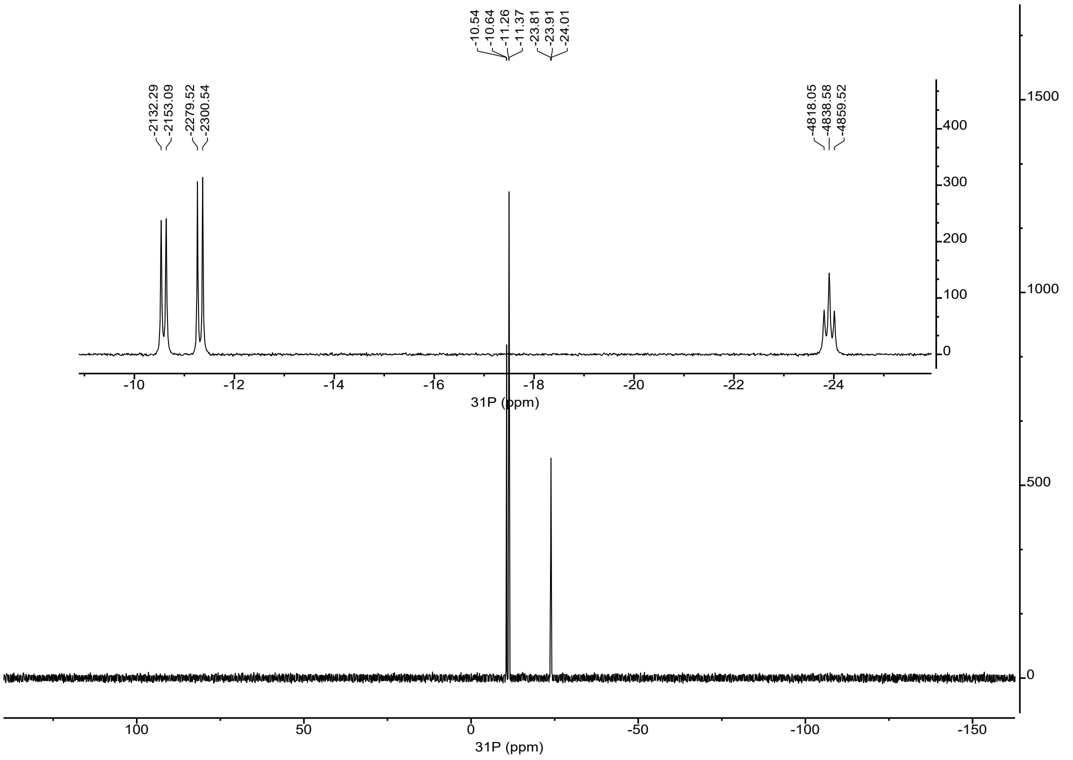

^{1}H, ^{13}C, ^{31}P NMR spectra of compound 13:

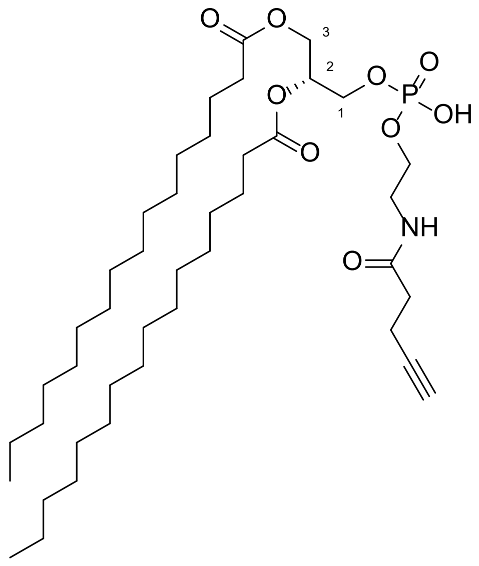

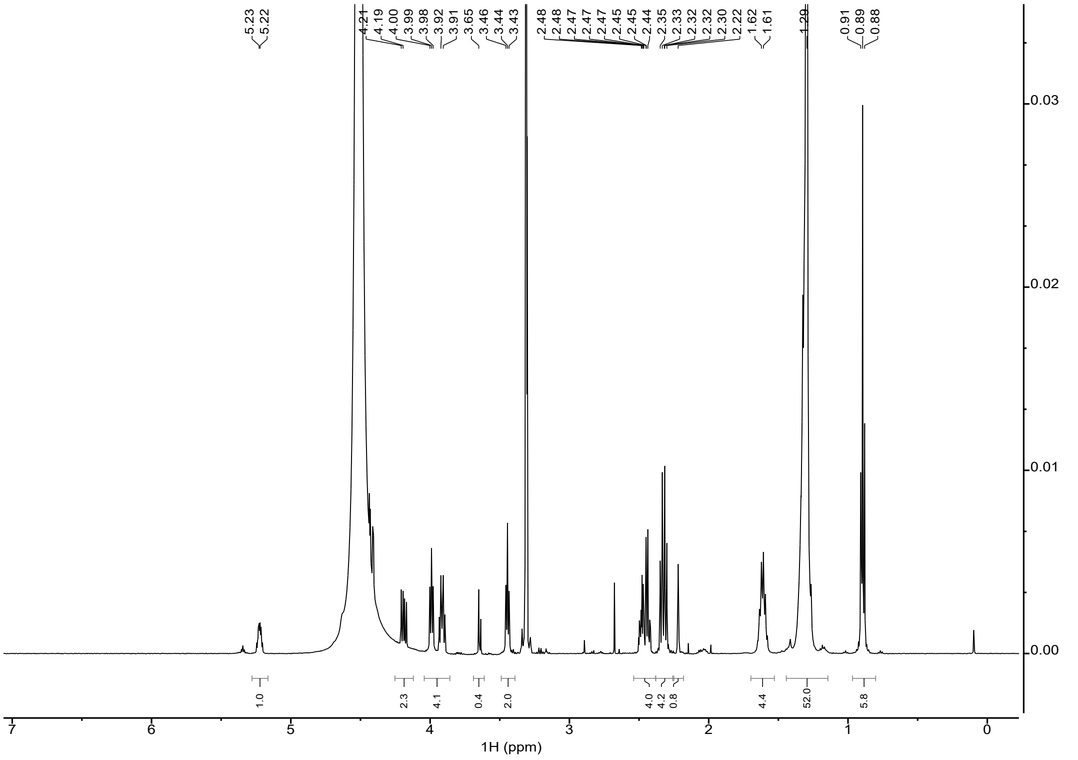
5.23
5.22
4.21
4.19
4.00
3.99
3.98
3.92
3.91
3.65
3.46
3.44
3.43
2.48
2.48
2.47
2.47
2.47
2.45
2.45
2.44
2.35
2.33
2.32
2.32
2.30
2.22
1.62
1.61
1.29
0.91
0.89
0.88
0.03
0.02
0.01
0.00
1.0
2.3
4.1
0.4
2.0
4.0
4.2
0.8
4.4
52.0
5.8
7
6
5
4
3
2
1
0
1H (ppm)

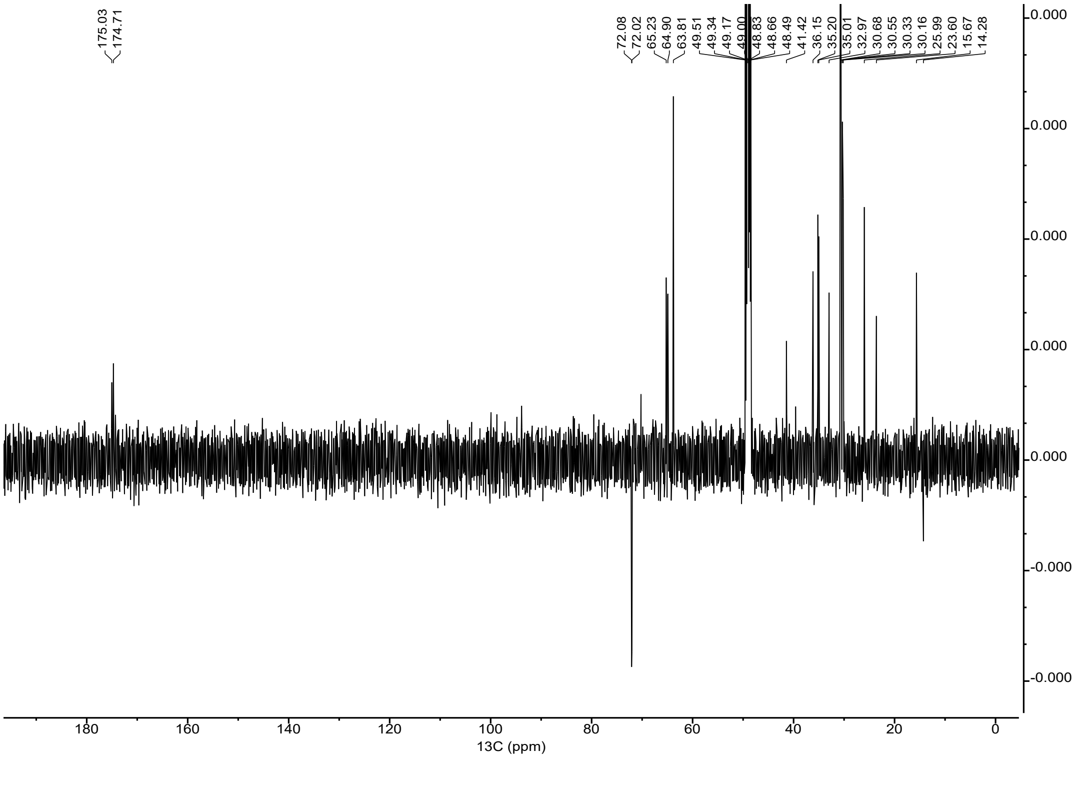
175.03
174.71
72.08
72.02
65.23
64.90
63.81
49.51
49.34
49.17
49.00
48.83
48.66
48.49
41.42
36.15
35.20
35.01
32.97
30.68
30.55
30.33
30.16
25.99
23.60
15.67
14.28
0.000
0.000
0.000
0.000
0.000
-0.000
-0.000
180
160
140
120
100
80
60
40
20
0
13C (ppm)

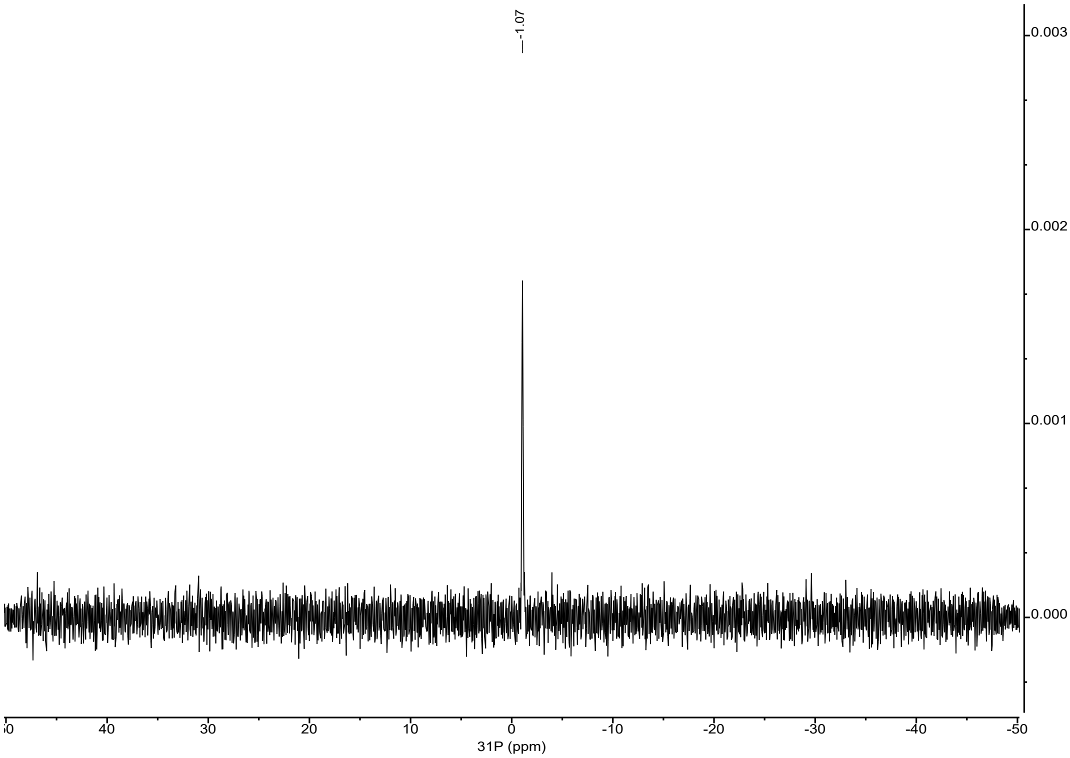

^{1}H, ^{13}C, ^{31}P NMR spectra of compound 14, dA^{DPPE}TP:

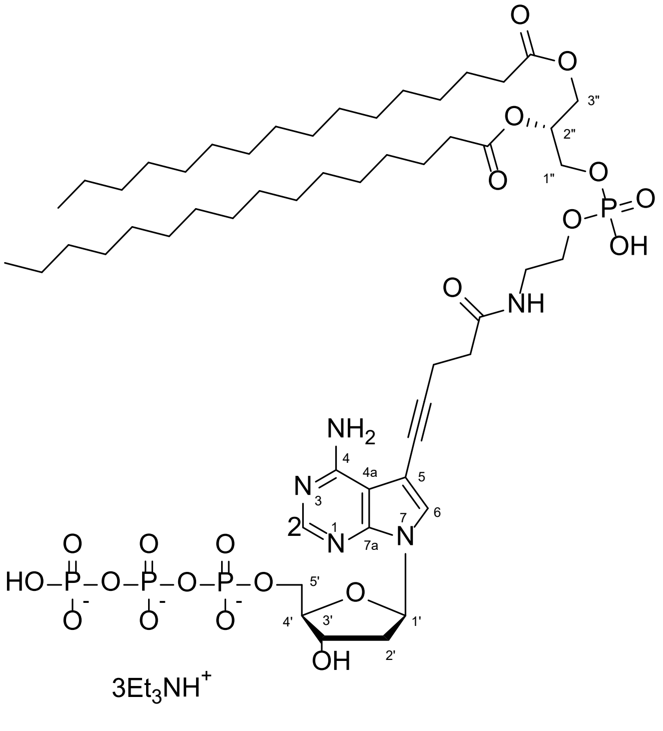

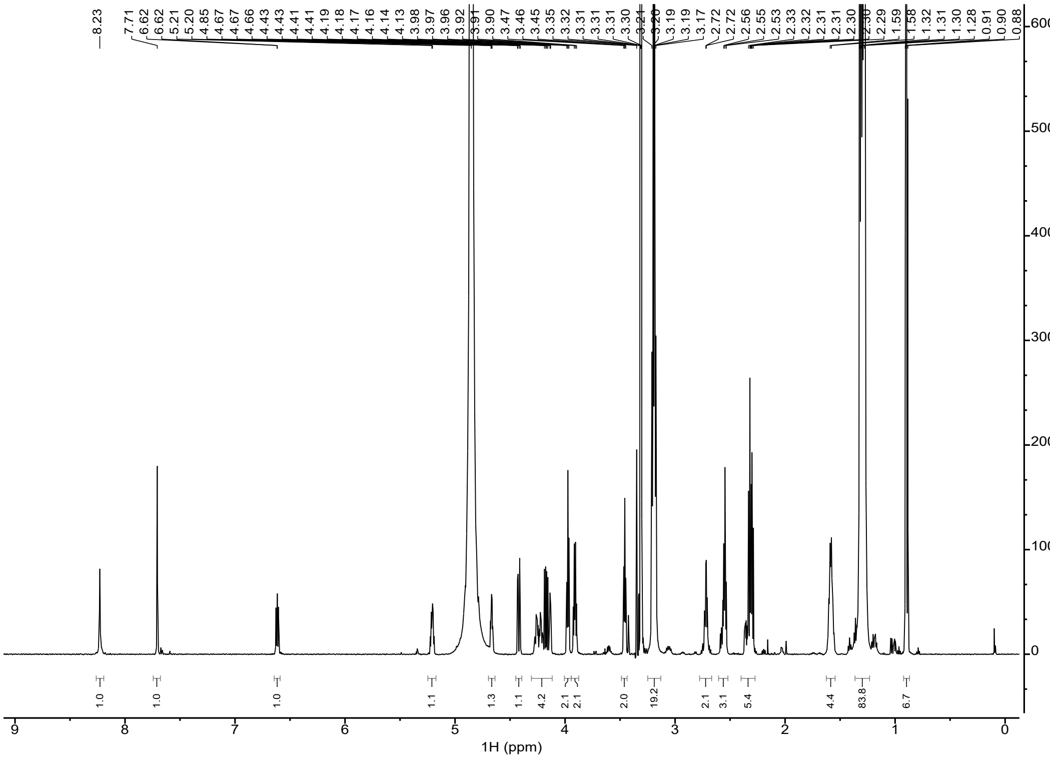

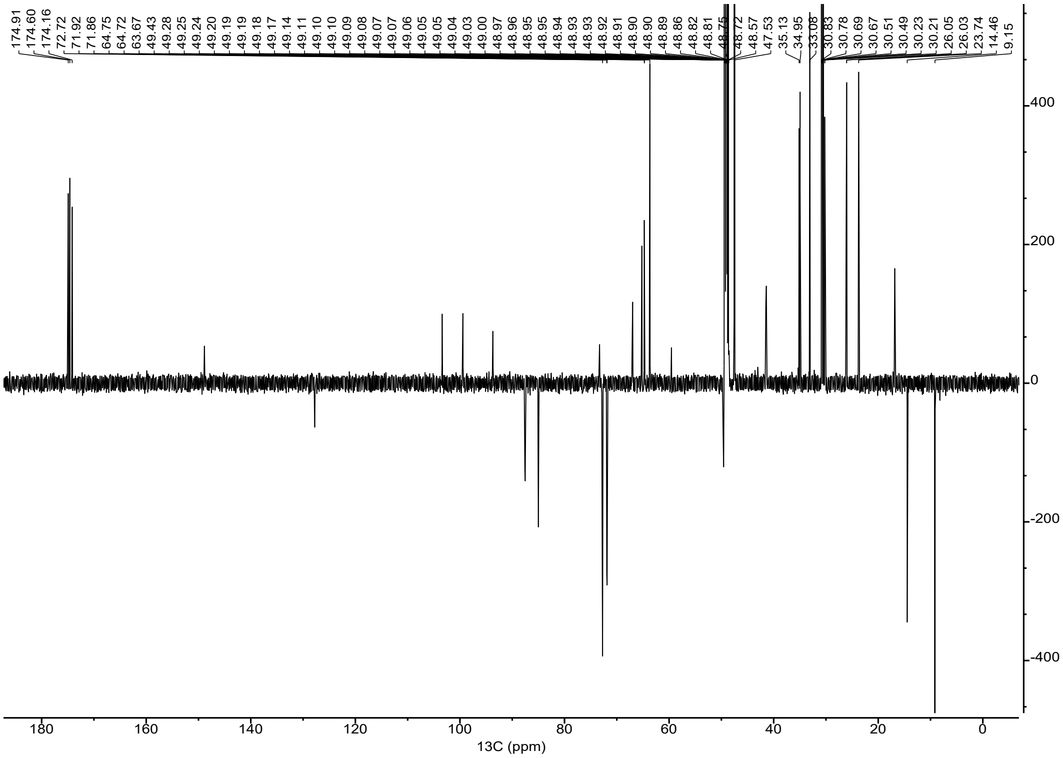

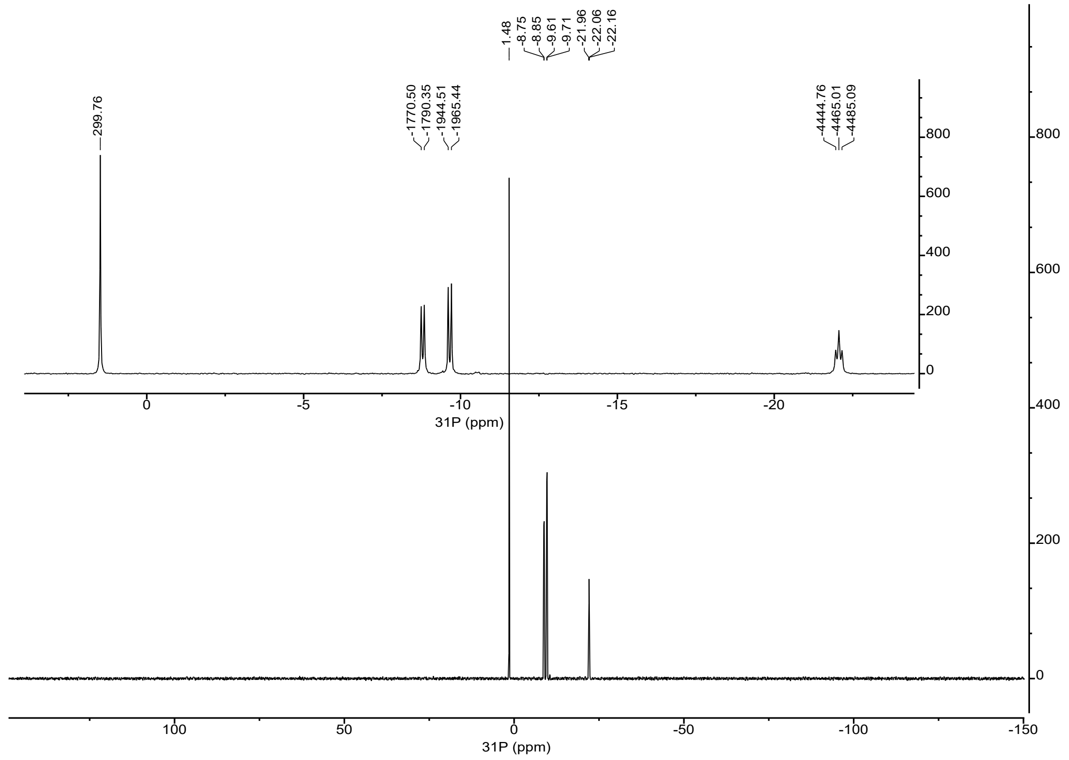

^{1}H, ^{13}C, ^{31}P NMR spectra of compound 15:

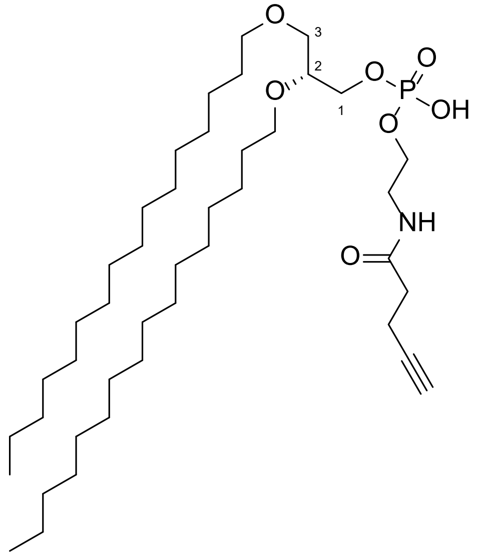

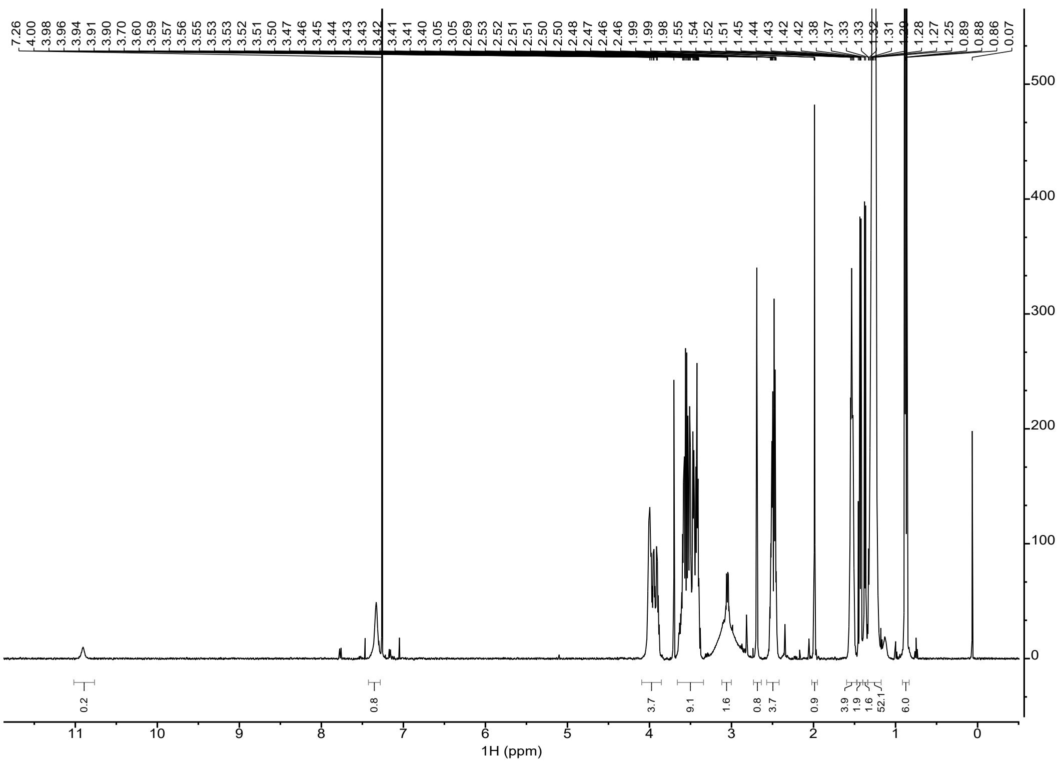

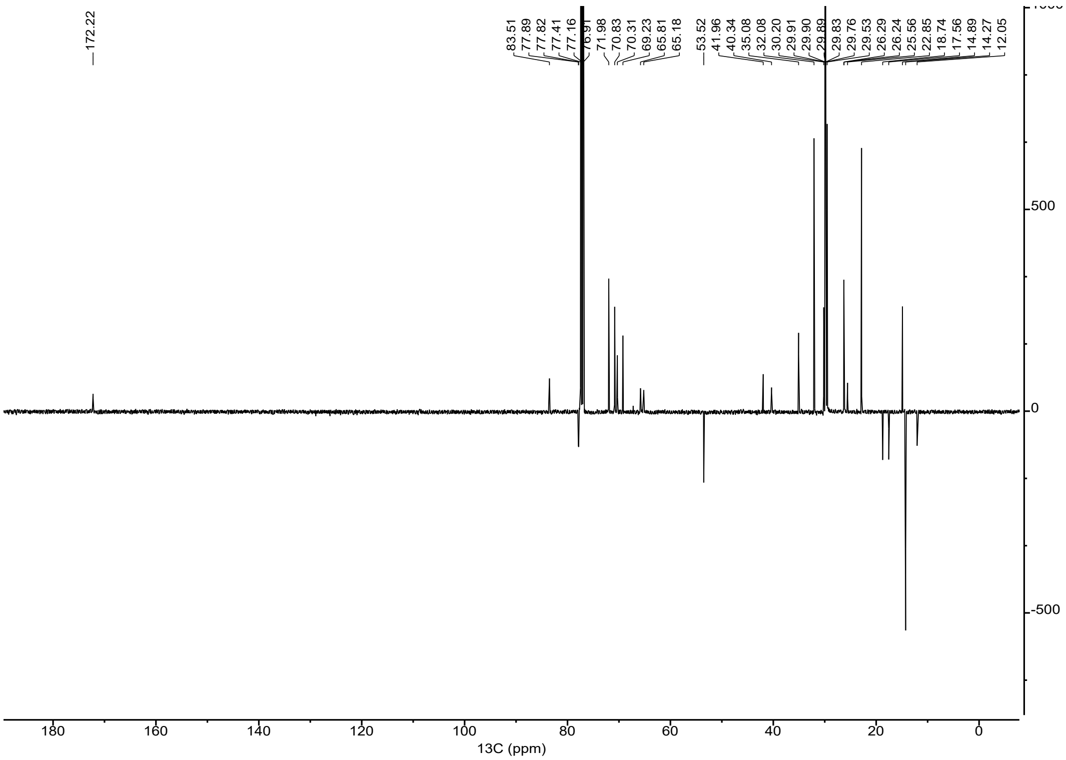

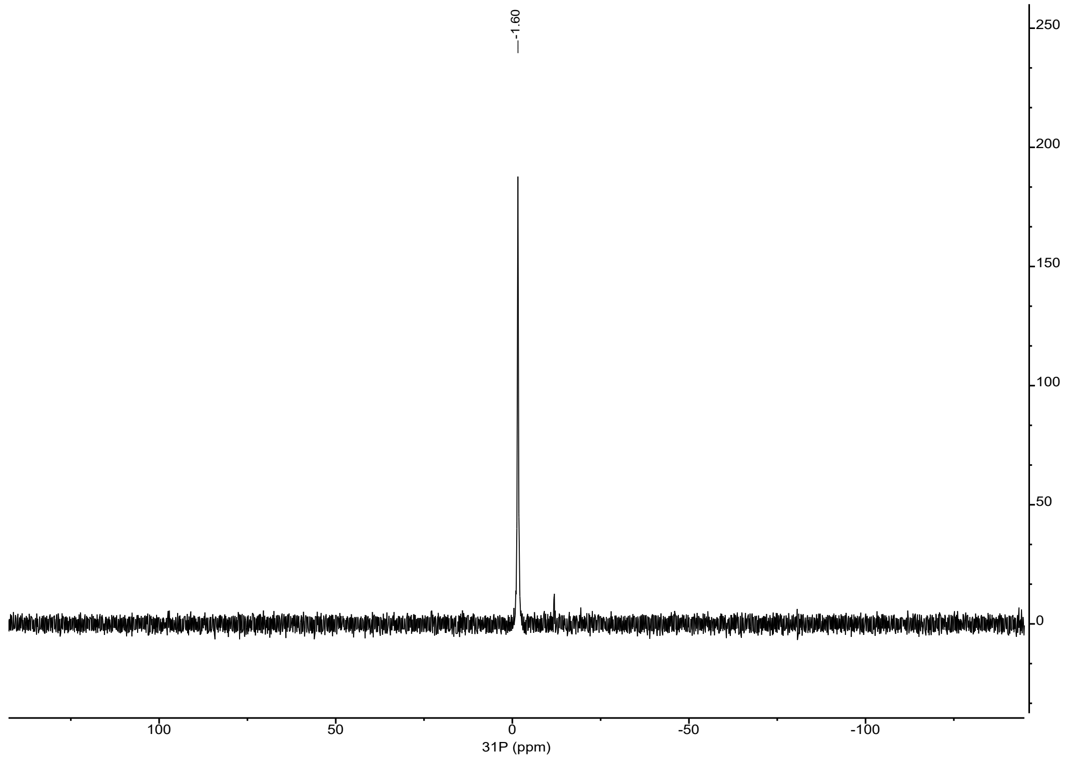

^{1}H, ^{13}C, ^{31}P NMR spectra of compound 16, dT^{DHPE}TP:

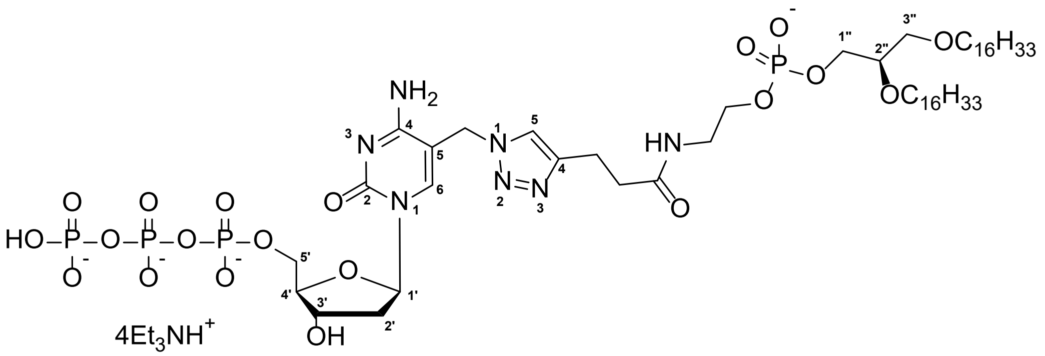

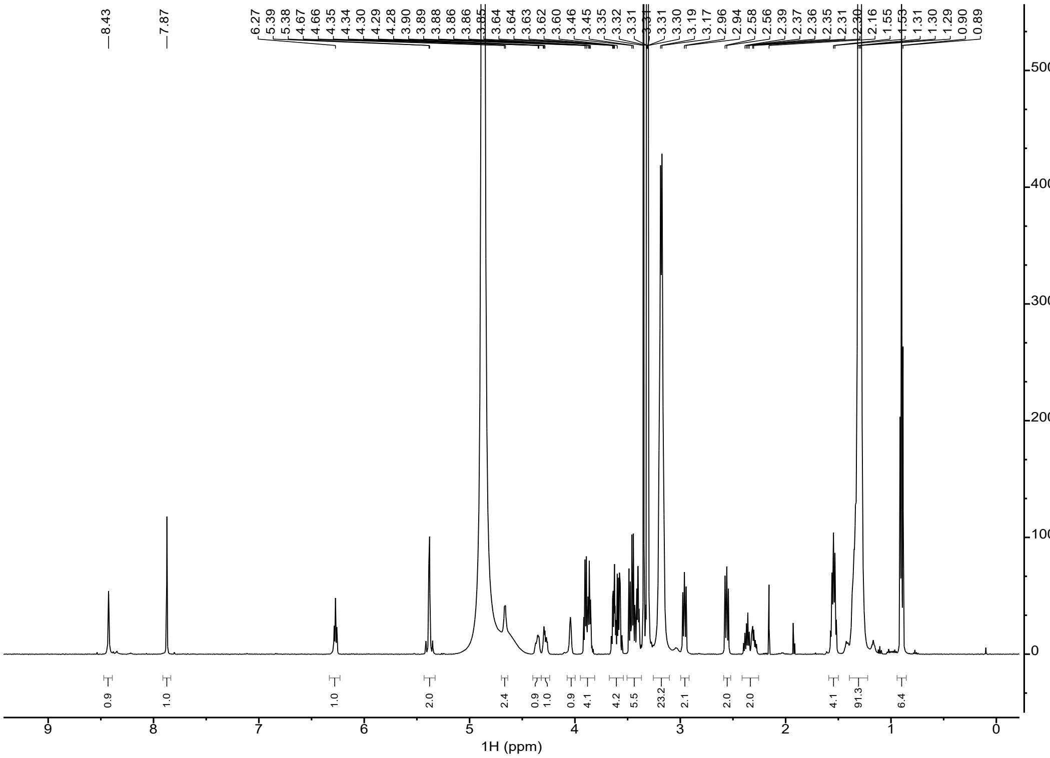
1H (ppm)

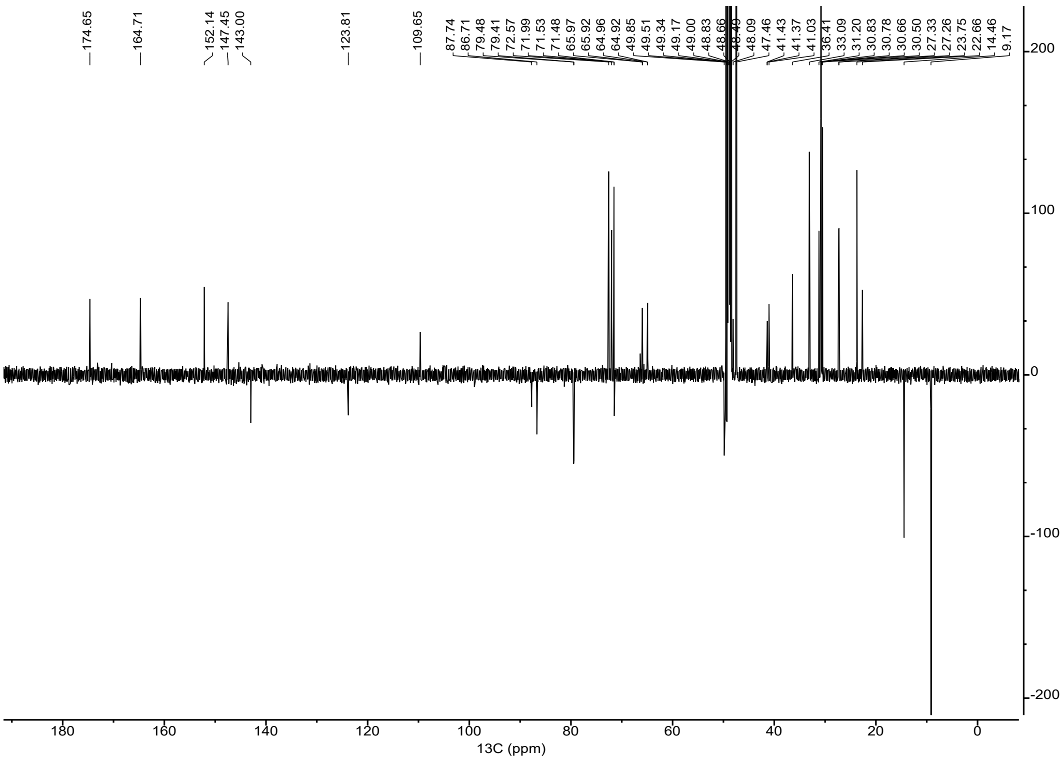
13C (ppm)

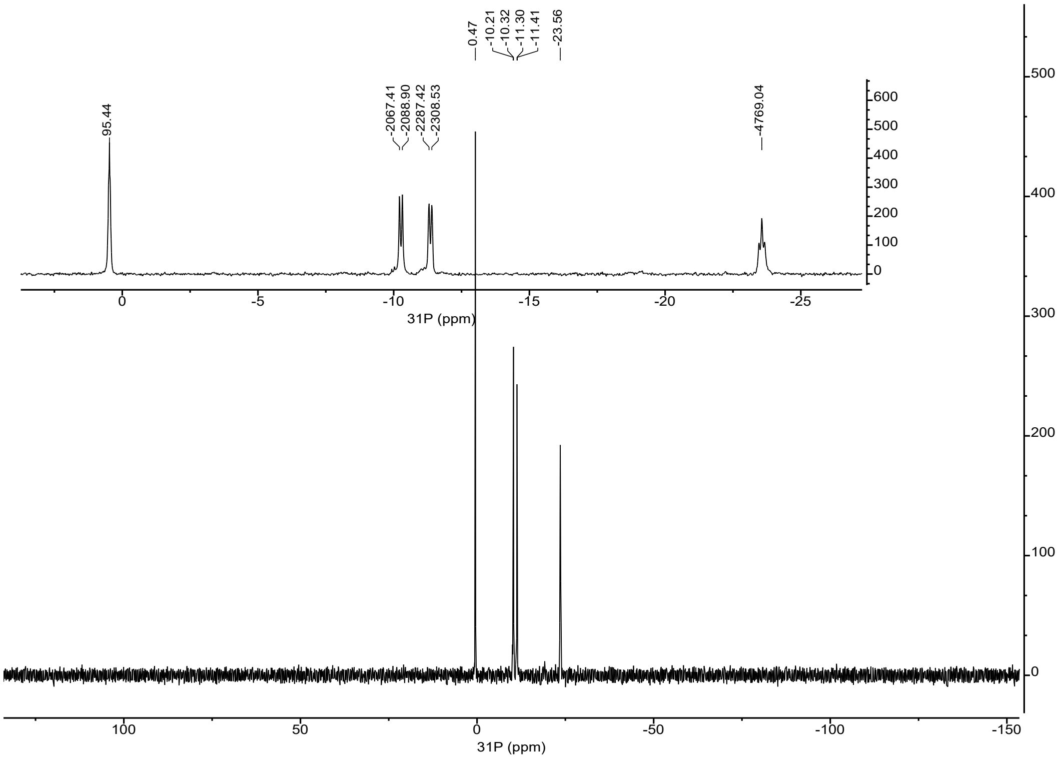
0.47
-10.21
-10.32
-11.30
-11.41
-23.56
95.44
-2067.41
-2088.90
-2287.42
-2308.53
-4769.04
31P (ppm)
31P (ppm)

5. References

1. M. J. Clemente, J. Fitremann, M. Mauzac, J. L. Serrano and L. Oriol, *Langmuir*, 2011, **27**, 15236-15247.
2. F. Boeck, T. Kribber, L. Xiao and L. Hintermann, *J. Am. Chem. Soc.*, 2011, **133**, 8138-8141.
3. S. Liao, H. Xu, B. Yang, J. Wang, X. Zhou, X. Lin and Y. Liu, *Org. Biomol. Chem.*, 2021, **19**, 8735-8739.

Full reference 45:

Bentley, D. R.; Balasubramanian, S.; Swerdlow, H. P.; Smith, G. P.; Milton, J.; Brown, C. G.; Hall, K. P.; Evers, D. J.; Barnes, C. L.; Bignell, H. R.; Boutell, J. M.; Bryant, J.; Carter, R. J.; Keira Cheetham, R.; Cox, A. J.; Ellis, D. J.; Flatbush, M. R.; Gormley, N. A.; Humphray, S. J.; Irving, L. J.; Karbelashvili, M. S.; Kirk, S. M.; Li, H.; Liu, X.; Maisinger, K. S.; Murray, L. J.; Obradovic, B.; Ost, T.; Parkinson, M. L.; Pratt, M. R.; Rasolonjatovo, I. M. J.; Reed, M. T.; Rigatti, R.; Rodighiero, C.; Ross, M. T.; Sabot, A.; Sankar, S. V.; Scally, A.; Schroth, G. P.; Smith, M. E.; Smith, V. P.; Spiridou, A.; Torrance, P. E.; Tzonev, S. S.; Vermaas, E. H.; Walter, K.; Wu, X.; Zhang, L.; Alam, M. D.; Anastasi, C.; Aniebo, I. C.; Bailey, D. M. D.; Bancarz, I. R.; Banerjee, S.; Barbour, S. G.; Baybayan, P. A.; Benoit, V. A.; Benson, K. F.; Bevis, C.; Black, P. J.; Boodhun, A.; Brennan, J. S.; Bridgham, J. A.; Brown, R. C.; Brown, A. A.; Buermann, D. H.; Bundu, A. A.; Burrows, J. C.; Carter, N. P.; Castillo, N.; Chiara E Catenazzi, M.; Chang, S.; Neil Cooley, R.; Crake, N. R.; Dada, O. O.; Diakoumakos, K. D.; Dominguez-Fernandez, B.; Earnshaw, D. J.; Egbujor, U. C.; Elmore, D. W.; Etchin, S. S.; Ewan, M. R.; Fedurco, M.; Fraser, L. J.; Fuentes Fajardo, K. V.; Scott Furey, W.; George, D.; Gietzen, K. J.; Goddard, C. P.; Golda, G. S.; Granieri, P. A.; Green, D. E.; Gustafson, D. L.; Hansen, N. F.; Harnish, K.; Haudenschild, C. D.; Heyer, N. I.; Hims, M. M.; Ho, J. T.; Horgan, A. M.; Hoschler, K.; Hurwitz, S.; Ivanov, D. V.; Johnson, M. Q.; James, T.; Huw Jones, T. A.; Kang, G.-D.; Kerelska, T. H.; Kersey, A. D.; Khrebtukova, I.; Kindwall, A. P.; Kingsbury, Z.; Kokko-Gonzales, P. I.; Kumar, A.; Laurent, M. A.; Lawley, C. T.; Lee, S. E.; Lee, X.; Liao, A. K.; Loch, J. A.; Lok, M.; Luo, S.; Mammen, R. M.; Martin, J. W.; McCauley, P. G.; McNitt, P.; Mehta, P.; Moon, K. W.; Mullens, J. W.; Newington, T.; Ning, Z.; Ling Ng, B.; Novo, S. M.; O'Neill, M. J.; Osborne, M. A.; Osnowski, A.; Ostadan, O.; Paraschos, L. L.; Pickering, L.; Pike, A. C.; Pike, A. C.; Chris Pinkard, D.; Pliskin, D. P.; Podhasky, J.; Quijano, V. J.; Raczy, C.; Rae, V. H.; Rawlings, S. R.; Chiva Rodriguez, A.; Roe, P. M.; Rogers, J.; Rogert Bacigalupo, M. C.; Romanov, N.; Romieu, A.; Roth, R. K.; Rourke, N. J.; Ruediger, S. T.; Rusman, E.; Sanches-Kuiper, R. M.; Schenker, M. R.; Seoane, J. M.; Shaw, R. J.; Shiver, M. K.; Short, S. W.; Sizto, N. L.; Sluis, J. P.; Smith, M. A.; Ernest Sohna Sohna, J.; Spence, E. J.; Stevens, K.; Sutton, N.; Szajkowski, L.; Tregidgo, C. L.; Turcatti, G.; Vandevondele, S.; Verhovsky, Y.; Virk, S. M.; Wakelin, S.; Walcott, G. C.; Wang, J.; Worsley, G. J.; Yan, J.; Yau, L.; Zuerlein, M.; Rogers, J.; Mullikin, J. C.; Hurles, M. E.; McCooke, N. J.; West, J. S.; Oaks, F. L.; Lundberg, P. L.; Klenerman, D.; Durbin, R.; Smith, A. J. Accurate whole human genome sequencing using reversible terminator chemistry. *Nature* **2008**, *456*, 53–59.
